# The role of methylation and structural variants in shaping the recombination landscape of barley

**DOI:** 10.1111/tpj.71171

**Published:** 2026-10-11

**Authors:** Federico Casale, Christopher Arlt, Marius Kühl, Jinquan Li, Julia Engelhorn, Thomas Hartwig, Benjamin Stich

**Affiliations:** ^1^ Institute of Quantitative Genetics and Genomics of Plants Heinrich Heine University Universitätsstraße 1 Düsseldorf 40225 Germany; ^2^ Julius Kühn‐Institut, Federal Research Center for Cultivated Plants Institute for Breeding Research on Agricultural Crops Sanitz 18190 Germany; ^3^ Max Planck Institute for Plant Breeding Research Köln 50829 Germany; ^4^ Institute for Molecular Physiology Heinrich Heine University Universitätsstraße 1 Düsseldorf 40225 Germany; ^5^ Cluster of Excellence on Plant Sciences, From Complex Traits Towards Synthetic Modules Universitätsstraße 1 Düsseldorf 40225 Germany; ^6^ Present address: Strube D&S GmbH Hauptstrasse 1 Söllingen 38387 Germany

**Keywords:** recombination rate, crossover, hotspot, methylation, structural variant

## Abstract

Meiotic recombination is not only a key mechanism for sexual adaptation in eukaryotes but crucial for the accumulation of beneficial alleles in breeding populations. The effective manipulation of recombination requires, however, a better understanding of the mechanisms regulating the rate and distribution of recombination events in genomes. Here, we identified the genomic features that best explain the recombination variation among a diverse set of segregating populations of barley at a resolution of 1 Mbp and investigated how methylation and structural variants (SVs) determine crossover (CO) hotspots and coldspots at a high resolution of 10 kb. Hotspots were found to be in proximity to genes, and the genetic effects not assigned to methylation were found to be the most important factor explaining differences in recombination rates among populations, along with the methylation and the parental sequence divergence. Interestingly, coldspots were associated with the inheritance of a highly methylated genomic fragment from one parent only, but hotspots were identified at genomic segments where both parents were equally low methylated. The parental sequence divergence was shown to have a sigmoidal correlation with recombination, indicating an upper limit of mismatch among homologous chromosomes for CO formation. SVs were shown to be correlated with CO suppression, and their type and size were not found to influence that effect. The joint act of methylation and SVs was associated with the location of coldspots in barley, and the weight of their relative effect depends on the genomic region. Our findings suggest that recombination in barley is highly predictable, occurring mostly in multiple short sections located in proximity to genes and being modulated by local levels of methylation and SV load.

## Significance Statement

Meiotic recombination is essential for generating genetic diversity, yet the genomic factors shaping its distribution remain incompletely understood. By analyzing recombination across diverse barley populations at multiple genomic scales, we show that hotspots occur in multiple short, gene‐proximal regions, while methylation and structural variation jointly shape recombination coldspots, with an asymmetric parental effect of methylation and region‐dependent contributions of these factors, providing new insights into how genetic, epigenetic, and structural variation jointly determine the recombination landscape.

## INTRODUCTION

In sexual reproduction, the recombination between the maternal and paternal homologous chromosomes followed by their random assortment during meiosis generates new combinations of alleles that can be transmitted to the next generation (Barton & Charlesworth, 1998). This mechanism for generating genetic diversity is widely conserved among eukaryotes since it provides a possibility for the adaptation of species: The reshuffling of alleles breaks the linkage between beneficial and deleterious mutations, allowing the accumulation of beneficial mutations into one haplotype and ultimately generating new phenotypes upon which selection can act (Muller, 1932; Peck, 1994).

Meiosis consists of a single round of DNA replication in which the bivalent is generated—a pair of physically linked homologous chromosomes each composed of two replicated sister chromatids—followed by two rounds of chromosome segregation (for a review see Mercier et al., 2015). During the first round, meiotic recombination occurs in prophase I when programmed DNA double‐strand breaks (DSBs) are repaired as either crossovers (COs), the reciprocal exchange of large regions between homologous nonsister chromatids, or noncrossovers (NCOs)—the unidirectional copies of small fragments from any of the intact homologous chromatids (Szostak et al., 1983). In addition, when both CO and NCO occur, mismatches at the site of the strand invasion are produced if sequence polymorphisms exist among homologous chromosomes. Such mismatches are either restored to the original allelic state (i.e., intersister repair) or repaired in favor of the homologous allele resulting in gene conversion (GC): the nonreciprocal exchange of alleles between homologous nonsister chromatids (Burt, 2000; Wijnker et al., 2013).

The rate of recombination events is strongly regulated and has been proposed to be related to an optimal level of recombination for adaptation in eukaryotes (Mercier et al., 2015). The presence of at least one CO per bivalent, termed the obligate CO, is required for the correct segregation of homologous chromosomes (Hall, 1972). In addition, the number of generated DSBs exceeds the number of observed COs, which are rarely more than three per chromosome per meiosis in most species (Baudat & Massy, 2007; Bauer et al., 2013; Martini et al., 2006).

Most of the observed COs, furthermore, prevail in small regions of a few kilobases called recombination hotspots where CO rates are several times greater than the chromosome average (Choi & Henderson, 2015; Mézard, 2006). Remarkably, both the rate and distribution of COs in the genome have been shown to exhibit extensive inter‐ and intraspecific variation (Lawrence et al., 2017; Nachman, 2002; Ritz et al., 2017). The mechanisms behind such variation, however, are not completely understood. Such knowledge is required for the effective manipulation of recombination, for example, for the purpose of breeding. Recombination determines the frequency of breaking undesirable linkages and stacking favorable alleles in the genetic background of breeding populations, and the resolution to which we can accurately map quantitative traits (Bauer et al., 2013; Blary & Jenczewski, 2019).

Earlier studies provided valuable information for deciphering the mechanisms behind recombination variation in plants. Most of the recombination in plants occurs in euchromatic regions where chromatin is accessible while heterochromatin is suppressed for meiotic recombination (Henderson, 2012). For example, earlier studies in *Arabidopsis thaliana* and other species have shown that recombination events tend to occur in genomic regions with hypomethylated DNA (Apuli et al., 2020; Fernandes et al., 2024; Marand et al., 2019; Rodgers‐Melnick et al., 2015; Yelina et al., 2012) and depleted nucleosome density (Choi et al., 2013; Wijnker et al., 2013). Moreover, COs in plants are typically located in close proximity to genes and in association with chromatin marks that favor transcription (Choi et al., 2013; Mercier et al., 2015). A positive correlation between the recombination rate and gene density has been observed in many plant families (Apuli et al., 2020; Brazier & Glémin, 2022; Choi et al., 2013; Gion et al., 2016; Paape et al., 2012; Silva‐Junior & Grattapaglia, 2015; Wang et al., 2016), including most grasses (Bauer et al., 2013; Casale et al., 2022; Darrier et al., 2017; Gardiner et al., 2019; Jordan et al., 2018; Marand et al., 2019). It is worth noting that some studies have instead reported a negative correlation between recombination rate and gene density (Giraut et al., 2011; Kim et al., 2007; Rodgers‐Melnick et al., 2015; Yang et al., 2012).

Polymorphisms among homologous chromosomes are expected to prevent recombination due to defective strand invasion and homology pairing caused by the increase in mismatches among nonsister chromatids (Henderson, 2012). Earlier studies in plants, however, reported contrasting correlations between recombination rate and parental sequence divergence (Bauer et al., 2013; Jordan et al., 2018; Marand et al., 2019; Saintenac et al., 2011; Salomé et al., 2012; Yang et al., 2012). Recent reports in Arabidopsis suggested that the recombination rate has a positive correlation with parental allelic divergence until a level of mismatch among homologous chromosomes prevents CO formation (Blackwell et al., 2020; Hsu et al., 2022). Accordingly, large structural variants (SVs) were shown to suppress local recombination in several plant species (Fernandes et al., 2024; Rodgers‐Melnick et al., 2015; Rowan et al., 2019; Shen et al., 2019).

The above‐mentioned contrasting findings impose the necessity to characterize the associations between recombination and genomic features at the species level to avoid making incorrect assumptions. In addition, due to differences in the employed research methods, disagreements were observed among studies on the same species (Apuli et al., 2020). In this respect, most reported recombination rates in plants were calculated based on a coarse genomic resolution that failed to capture the complete genetic variation generated from meiosis. At present, most reported recombination assessments at high resolution have been performed in Arabidopsis (Fernandes et al., 2024; Lu et al., 2012; Rowan et al., 2019; Sun et al., 2012; Wijnker et al., 2013; Yang et al., 2012), while only a few have been performed in major cereal crops such as maize (*Zea mays*) (Li et al., 2015; Rodgers‐Melnick et al., 2015), rice (*Oryza sativa*) (Marand et al., 2019; Si et al., 2015), and wheat (*Triticum aestivum*) (Jordan et al., 2018). In barley (*Hordeum vulgare*), the fourth of this list, earlier low‐resolution recombination studies successfully revealed the genetic basis of recombination as well as the association of recombination with some environmental and genomic features on a broad genomic scale (Arrieta et al., 2021; Casale et al., 2022; Colas et al., 2016, 2019; Dreissig et al., 2019, 2020; Higgins et al., 2012 and Schreiber et al., 2022); however, a high‐resolution study depicting the complete meiotic recombination landscape and the respective associations of genetic and epigenetic features in the barley genome is still lacking.

Consequently, in the present study, we aimed to (i) identify the genomic features that best explain the recombination variation among genetically diverse barley populations, (ii) detect recombination events in the barley genome at high resolution (10 kb), and (iii) analyze the effect of genomic features in determining the location of recombination hotspots and coldspots in the genome.

## RESULTS

### The genomic features associated with recombination rate variation along the barley genome

The association of genomic features with the recombination rate variation along the genome was firstly investigated at a low resolution (1 Mbp) using average values across the 45 DRR populations that cover a wide genetic diversity of cultivated barley. The recombination rate was low in the pericentromeric region and higher in the distal regions of the chromosomes, which also had greater gene density (Figure 1A). The SV load, the physical fraction spanned by the analyzed SVs (insertions, deletions, inversions, duplications, and translocations) increased toward the distal regions of the chromosomes as did the recombination rate (Figure 1A and Figure S1). In this way, the presence of SVs showed a significant positive Pearson's correlation (*P* < 0.001) of 0.56 with the recombination rate along the distal regions of the chromosomes (Figure 1C and Figure S3A), whereas a lower but significant correlation (*P* < 0.001) of 0.13 was observed in the pericentromeric region (Figure 1D and Figure S3B). The sequence divergence among the parental inbred lines, which showed a significant positive correlation (*P* < 0.001) with the SV load of 0.27, was found to have a significant positive correlation (*P* < 0.001) with recombination rates of 0.23 in the distal regions but only of (*P* < 0.05) 0.06 in the pericentromeric regions (Figure 1A, C and D). The methylation level in the three sequence contexts differed along the barley chromosomes, where the CpG and CHG contexts reached a maximum in the pericentromeric region and decreased toward the telomeres, while the methylation level in the CHH context increased toward the telomeres (Figure 1B). The CpG and CHG contexts had higher overall methylation levels than did the CHH context, thus a recombination effect on the recombination rate along the barley chromosomes is expected to be due to the CpG and CHG contexts but very little due to the CHH context (Figure 1B). The CpG and CHG contexts showed a significant negative correlation (*P* <0.001) of −0.87 and −0.89 with the recombination rate along the distal regions of the chromosomes (Figure 1C and Figure S3A), and a lower but significant correlation (*P* < 0.001) of −0.29 and −0.33 was observed in the pericentromeric region (Figure 1D and Figure S3B), respectively. The CHH contexts showed a significant positive correlation (*P* < 0.001) of 0.81 with the recombination rate along the distal regions of the chromosomes (Figure 1C and Figure S3A), whereas a lower but significant correlation (*P* < 0.001) of 0.16 was observed in the pericentromeric region (Figure 1D and Figure S3B).

**Figure 1:**
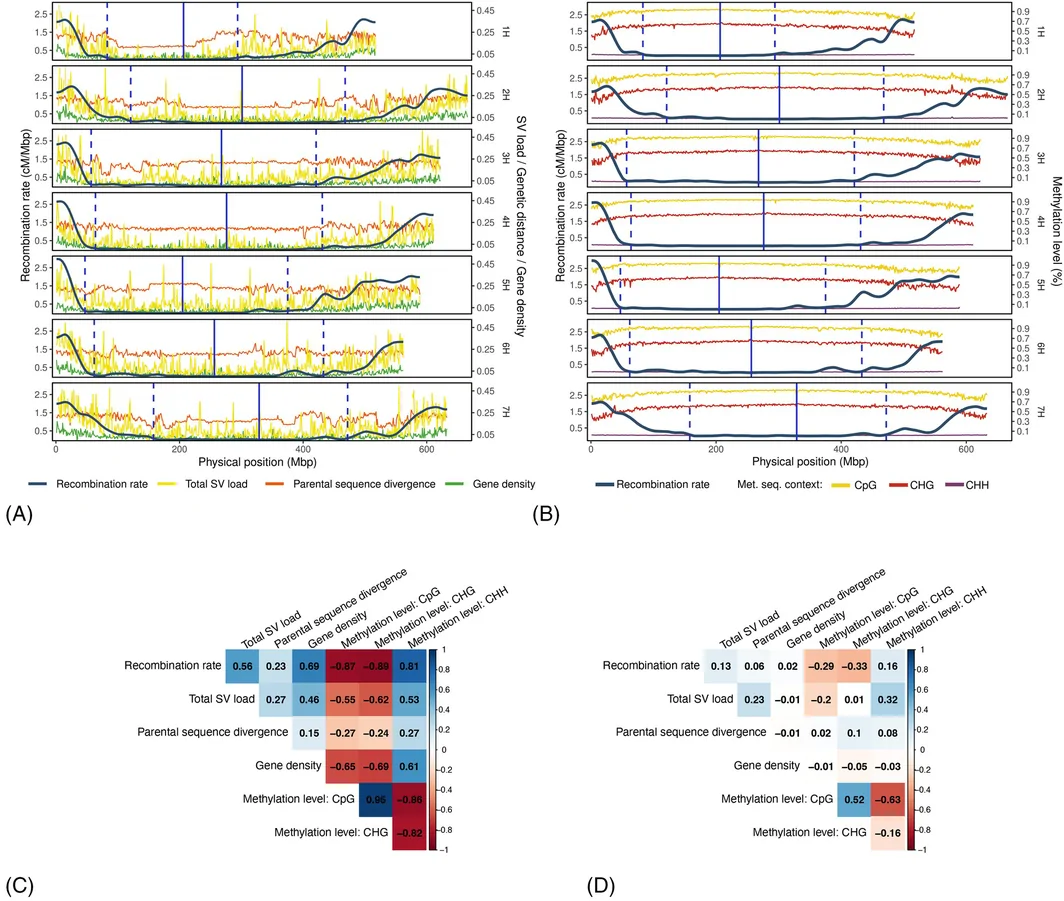
The genomic distribution of recombination rate, structural variants, parental sequence divergence, gene density, and DNA methylation across barley chromosomes, and their correlations in distal and pericentromeric regions. (A) Distribution of recombination rate, total structural variants' load fraction, gene density, and parental sequence divergence between the respective parental inbred lines on average across the 45 double round‐robin (DRR) populations in 1 Mbp windows across the seven barley chromosomes. The total structural variants (SV) load fraction represents the portion spanned of a given 1 Mbp genomic window by the sum of insertions, deletions, inversions, and duplications. (B) Distribution of recombination rate and methylation level in 1 Mbp windows for the sequence contexts CpG, CHG, and CHH, on average across the seven barley chromosomes and 45 DRR populations. The methylation level values for a given methylation sequence context represent the percentage of methylated reads of such context present in a 1 Mbp window averaged across the 45 DRR populations. The vertical blue solid line indicates the position of the centromere in the Morex v3 reference genome, and the vertical blue dashed lines indicate the pericentromeric region calculated across the 45 DRR populations. (C, D) Correlation matrix between recombination rate, total SV load fraction, parental sequence divergence, gene density, and methylation level for the sequence contexts CpG, CHG, and CHH, across 1 Mbp windows in the distal (C) and the pericentromeric (D) regions of the barley chromosomes for the average across the 45 DRR populations. Pearson's correlation coefficients are indicated with a color gradient from −1 (red) to 1 (blue).

### The genomic features associated with recombination rate variation among barley populations

The best subset of genomic features explaining the recombination rate variation among the 45 DRR populations in 1 Mbp genomic windows along the barley chromosomes was identified using a stepwise regression model (Figure 2). The genetic effects were found to be the most determining factor in explaining differences in recombination rate among populations with a promoting effect on both the distal (positive, 0.70) and the pericentromeric regions (positive, 0.27) of the chromosomes. The parental sequence divergence was also found to be positively correlated with the recombination rate in both mentioned chromosomal regions (positive, 0.28 and 0.11, respectively). A few windows showed a significant negative correlation between parental sequence divergence and the recombination rate (negative, 0.04 in both respective chromosomal regions). Notably, such windows with a negative correlation had a parental sequence divergence near its relative maximum, which appeared to be associated with a high SV load (Figure S4A). However, the physical fraction of 1 Mbp genomic windows spanned by all SVs was found to have a low (and mostly positive) correlation with the differences in recombination rate among populations (in distal regions, positive, 0.05; negative, 0.02). The methylation level in the three sequence contexts—CpG, CHG, and CHH—was negatively correlated with the recombination rate in the distal regions of the barley chromosomes (negative, 0.22, 0.13, and 0.09, respectively). However, such correlations were found to be low in the pericentromeric regions. The extended model revealed no impact of the different SV types on recombination rate variation among populations. (Figure S5 and Table S2).

**Figure 2:**
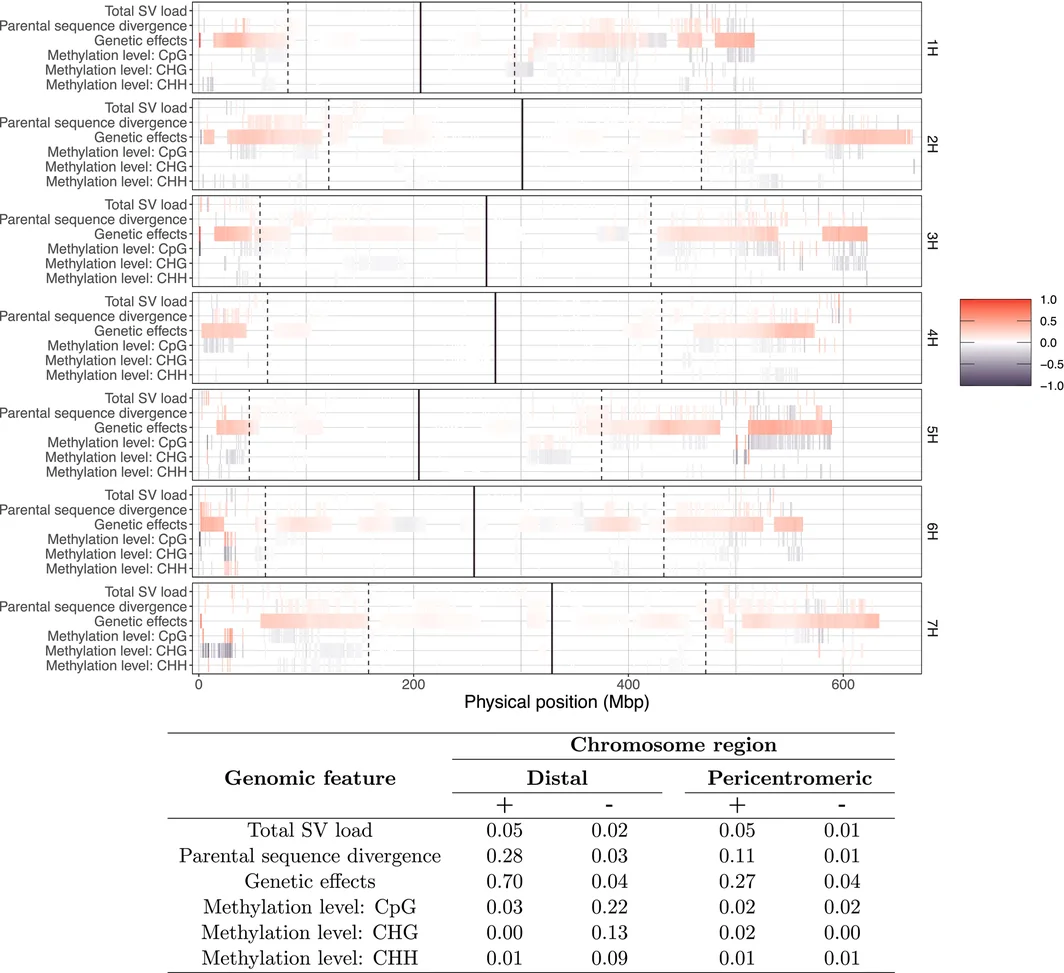
The genomic features explaining the variation in the recombination rate among the 45 double round‐ robin (DRR) populations in 1 Mbp windows across the seven barley chromosomes. The genomic features were selected for each window by a stepwise regression procedure. The standardized regression coefficients are indicated by a color gradient from −1 (purple) to 1 (red). The studied genomic features included sequence divergence among parental inbred lines, genetic effects, total structural variants (SV) load, and methylation level for the sequence contexts CpG, CHG, and CHH. The vertical solid line indicates the position of the centromere in the reference genome, and the vertical dashed lines indicate the pericentromeric region calculated across the 45 DRR populations. The analysis was performed separately for the distal and pericentromeric regions of the barley chromosomes, respectively. The fraction of 1 Mbp windows of the barley genome in which the genomic features were found to be significantly associated with the recombination rate variation across the 45 DRR populations are displayed in the table. The fractions of 1 Mbp windows positively and negatively associated with the recombination rate were indicated with the “+” or the “−” signs, respectively.

### The key parameters for detecting recombination events at high resolution

Three connected DRR populations (HvDRR13, HvDRR27, and HvDRR28) were selected randomly to investigate the association of recombination events with genomic features at a high resolution (10 kb). The mRNA sequencing of recombinant inbred lines (RILs) of the evaluated populations yielded 4.1 M sequence variants of which 858 K SNPs remained after being intersected with reported SNV parental data generated by DNA sequencing. A total of 12 K SNPs were added from the iSelect array, resulting in a total of approximately 870 K SNPs genome‐wide. After removing SNPs carrying nonparental alleles or with 100% missing data, the final number of genome‐wide SNPs ranged from 214 to 259 K per population (Table S3 and Figure S6). The median inter‐SNP distance was 132 bp on average across populations, which was 79 times shorter than the 10, 475 bp utilized to count COs for the same three populations analyzed in a previous study (Casale et al., 2022). RNA‐seq‐derived SNPs were distributed unevenly along the chromosomes, with a higher density of SNPs in the more distal chromosome regions and a relative depletion in pericentromeric regions (Figure S14). This pattern is consistent with the transcriptional origin of the SNP dataset, as pericentromeric regions are generally enriched in transposable elements and exhibit lower transcriptional activity, resulting in fewer expressed sequences from which RNA‐seq variants can be detected. The maximum inter‐SNP distance ranged from 5.45 to 11.99 Mbp among populations (Table S3), denoting large regions that were identical by descent (IBD) among the parental inbred lines involved in a given population and thus among their respective offspring. This resulted in an average density of 57 SNPs per Mbp across populations. The mean length for each parental SNP block category was 14.8, 44.1, and 111.5 Mbp for the short, medium, and long CO‐related blocks, respectively (Table S4). The block length was positively correlated with the number of SNPs per block (>0.75, *P* < 0.001). Therefore, the false‐positive rate for detecting CO was inversely proportional to the marker block length, supporting the identification of block length categories with different CO layers.

On average, we observed 30, 87, and 269 genome‐wide COs had accumulated per RIL across populations for the long, medium, and short block lengths, respectively (Table S5). Considering only the layer of COs generated by blocks longer than 3 Mbp (i.e., the long category), the genome‐wide CO counts per RIL ranged from 14 to 65 across populations (Figures S7 and S8). Since a given CO breakpoint was determined as the midpoint of the CO interval, the accuracy of the breakpoint location depended on the CO interval length (Table S6). The lengths of the detected CO intervals ranged from less than 20 bp to 10.8 Mbp with a median that varied from 41.9 kb to 151.6 kb depending on the considered CO layer. The average number of genome‐wide GC events that accumulated per RIL across populations was 58, 251, and 6,521 for long, medium, and short GC‐related block lengths, respectively. An average of 56 recombination hotspot windows per chromosome were found across the three selected populations (Table S7). Among these, 12 were found in the pericentromeric regions, and the rest were found in the distal regions of the barley chromosomes (Table S8 and Figure S9). The recombination hotspot windows contained 0.26, 0.14, and 0.25 of the total observed COs in the HvDRR13, HvDRR27, and HvDRR28 populations, respectively. Less than 10% of the hotspot windows in a given population were shared with another population, and less than 1% of the total counted hotspot windows were shared among the three analyzed populations (Figure S10). Interestingly, both the number and conservation level of coldspot windows far exceeded those of hotspots. On average, across populations, more than half (10,436 out of the 19,496) of the distal windows were recombination coldspots. More than 60% of the coldspot windows in a given population were shared with the other populations, and 16.7% of the coldspot windows were present in all three analyzed populations. The majority of the coldspot windows were located contiguously in regions with lengths that varied from 10 kb to 17 Mbp with an average of 322 kb across the three analyzed populations.

More than 15% of the GC hotspot windows in a given population were shared among the three analyzed populations (Figure S11). The GC hotspot windows were found to overlap with the CO hotspot windows in the distal region of the barley chromosome significantly more than they did under a random distribution across the three analyzed populations (Table S9). The GC hotspot windows detected in a given population overlapped with 12–15% of the CO hotspot windows in the same population and with 7.5–9.5% of the CO hotspot windows detected in the other two analyzed populations (Table S10). There was no significant (*P* > 0.05) difference between such overlap proportions in the HvDRR27 and HvDRR28 populations.

### The effect of methylation and structural variants on recombination rate variation at high resolution

#### The coldspot and hotspot windows have different methylation levels and SV load than other windows within the respective genomic regions

The coldspot and hotspot windows in a given chromosome region showed distinct methylation patterns compared to the remaining windows in the same region in the three analyzed populations (Figure 3). The methylation level of the CpG and CHG sequence contexts in the coldspot windows of both distal subregions was found to be significantly greater (*P* < 0.001) than that in the other windows within the same distal subregions. However, the methylation level in the coldspot windows of the distal telomeric subregion was significantly lower (*P* < 0.001) than that in the windows of the distal proximal subregion. The coldspot windows in such comparisons that were below the critical value (methylation levels of 0.89 and 0.59 for the sequence contexts CpG and CHG, respectively) were found to have a significantly (*P* < 0.001) greater total SV load fraction than the coldspot windows above the critical value (Table S11).

**Figure 3:**
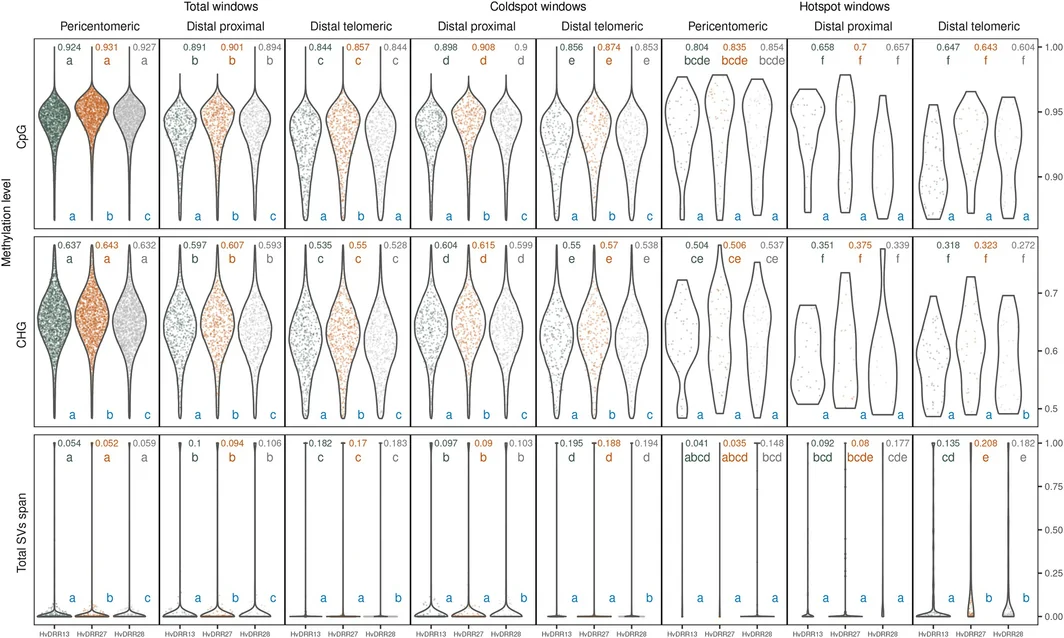
Distribution of the methylation level for the methylated sequence contexts CpG and CHG, and the total structural variants (SV) load fraction in 10 kb genomic windows grouped by their location in different chromosomal regions—pericentromeric, distal proximal, and distal telomeric—and recombination rate—total, coldspots, and hotspots—of the barley chromosomes in the analyzed double round‐robin (DRR) populations HvDRR13 (green), HvDRR27 (orange), and HvDRR28 (gray). For each DRR population, the methylation level at each sequence context in a given genomic window was calculated as the average among the respective parental inbred lines' methylation level values for the methylated cytosine positions in that window, weighted by the number of methylated cytosine positions corresponding to each parent. The displayed dots for chromosome regions and coldspots are a random subset of 1% of the total windows in each window group. For each genomic feature, the distribution's mean of each window group from a given population is indicated with the related population color at the top‐right of the respective plot. Significant differences (α = 0.001) in the Wilcoxon's rank‐sum test among such means across window groups are indicated with different letters below the respective mean and sharing the population color. For a given genomic feature in each window grouping category, significant differences (α = 0.008) in the Wilcoxon's rank‐sum test among populations are indicated with different blue letters at the bottom of each panel.

The methylation level in the CpG and CHG sequence contexts of the hotspot windows in the distal region was significantly lower (*P* < 0.001) than that across the other windows in any region of the barley chromosomes. The hotspot windows in the pericentromeric region were found to be significantly less methylated (*P* < 0.001) than the rest of the windows in that region, but not less than windows in the distal region.

In Figure 3, the average SV load across 10 kb windows is < 0.2. The coldspot windows in the distal telomeric regions were found to have a significantly greater total SV load (*P* < 0.001) than the rest of the windows in that subregion. By contrast, the coldspot windows in the distal proximal region did not show such an increase in total SV load. However, the coldspot windows below the critical value (SV loads equal to 0.187, 0.174, and 0.187 for the HvDRR13, HvDRR27, and HvDRR28 populations, respectively) had a significantly increased methylation level (*P* < 0.05) compared to the windows above the critical value for the CpG and CHG sequence contexts (Table S12). The hotspot windows were not observed to have a significantly different (*P* > 0.001) span of total SVs compared to the total windows in their respective chromosome regions. However, the observed overlaps between CO intervals and insertions/deletions and duplications were found to be significantly less frequent (*P* < 0.001) than such overlaps under a random distribution of the COs and the respective SVs in the distal regions of the barley chromosomes in the three analyzed populations (Table S13). In the case of inversions, such a pattern was observed only for the HvDRR27 population. Moreover, the distance between the CO breakpoints and the closest SV of any type was significantly greater (*P* < 0.001) than the CO‐SV distances expected by chance (Table S14).

#### The genomic environment neighboring hotspot and coldspot regions

The coldspot regions were found to have a significantly higher (*P* < 0.001) methylation level in the methylated sequence contexts CpG and CHG than adjacent 10 kb genomic windows in both distal subregions of the chromosome in the three analyzed populations (Figure 4). In addition, coldspot regions were found to have a significantly higher (*P* < 0.001) total SV load than the neighboring 10 kb windows in the considered range from −40 kb to +40 kb around the respective coldspot. The hotspot regions were not found to have significantly (*P* > 0.001) different methylation level or SV load than those in their adjacent 10 kb genomic windows in any analyzed chromosomal region or population.

**Figure 4:**
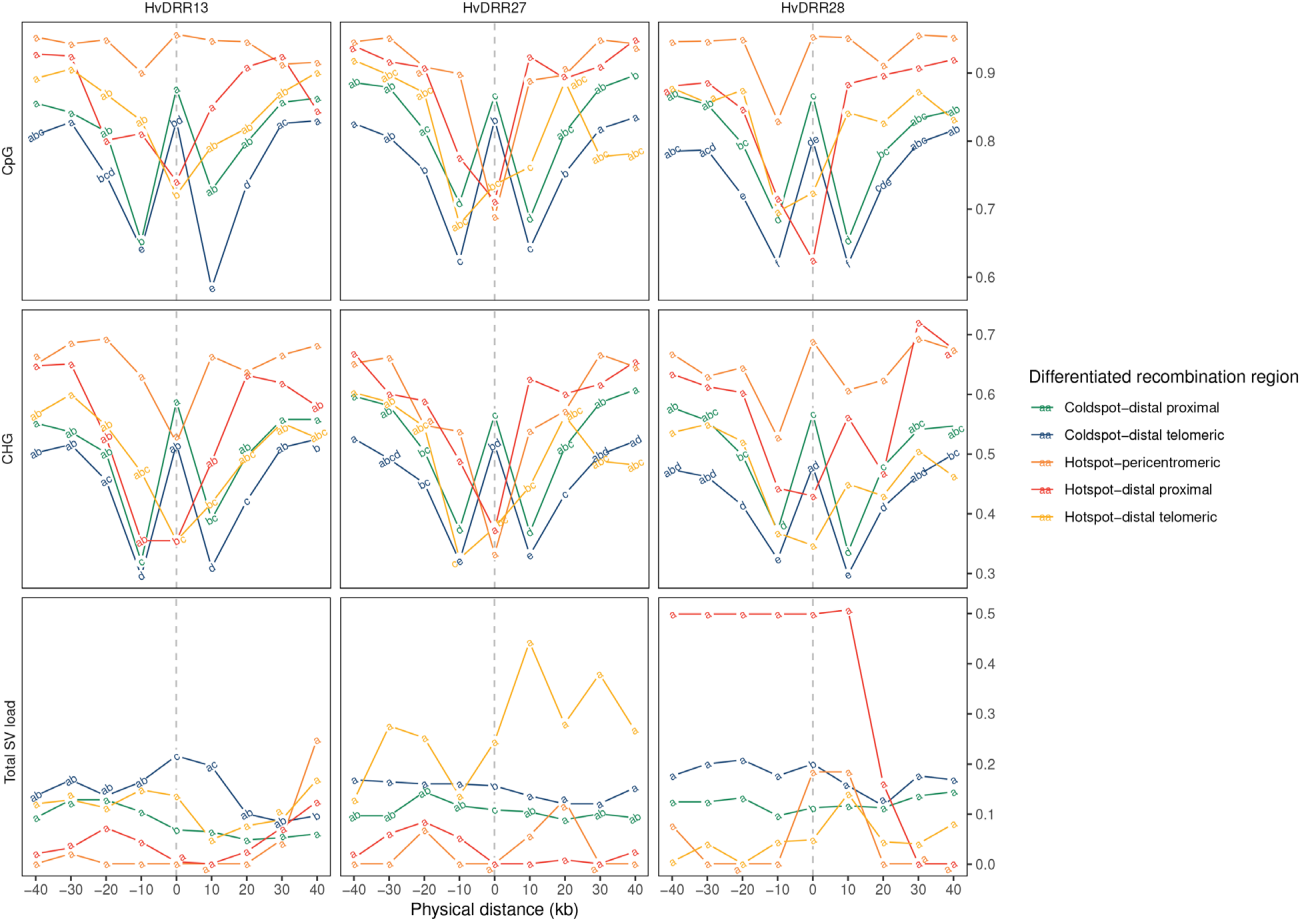
The mean values of the methylation level in the methylated sequence contexts CpG and CHG, and total structural variants (SV) load in the genomic windows grouped by the physical positions in the range from −40 kb to +40 kb around the coldspot and hotspot genomic regions in the pericentromeric, distal proximal, and distal telomeric region of the chromosomes in the analyzed double round‐robin (DRR) populations HvDRR13, HvDRR27, and HvDRR28. The vertical dashed lines indicate the relative location of either the coldspot or the hotspot windows, respectively. The significant difference (α = 0.001) in the Wilcoxon's rank‐sum test among the window groups corresponding to the different 10 kb physical positions neighboring the respective hotspot or coldspot region in a particular chromosome region is indicated with different letters.

The overlap between the coldspot regions and genes in the distal regions of the barley chromosomes was not significantly different (*P* > 0.001) from such overlap under a random distribution (Table 1). By contrast, the overlap between the hotspot regions and genes was found to be significantly (*P* > 0.001) greater than expected under a random distribution in the three analyzed populations, while the overlap between hotspots and intergenic regions was not found to be significantly (*P* < 0.001) greater than random, with the exception of the HvDRR27 population. In addition, a high proportion of the windows surrounding hotspot regions in both the proximal (0.37–0.49) and telomeric (0.33–0.45) subregions of the distal region of the genome were coldspot windows (Table S15).

**Table 1:** The observed overlaps among the recombination coldspot and hotspot 10 kb windows with genes and the intergenic regions in the distal region of the barley chromosomes, and their comparison with the overlaps generated under a random distribution of such regions in the analyzed double round‐robin (DRR) populations HvDRR13, HvDRR27, and HvDRR28. The random distribution of the genomic regions was simulated with 1000 permutations

Recombination region	Genetic region	Population	Observed overlaps	Random overlaps
Coldspot windows	Genes	HvDRR13	23 989	37 029
		HvDRR27	19 545	26 369
		HvDRR28	24 832	37 091
	Intergenic regions	HvDRR13	77 006	129 894
		HvDRR27	73 553	87 290
		HvDRR28	78 332	135 060
Hotspot windows	Genes	HvDRR13	1057***	237
		HvDRR27	739***	146
		HvDRR28	1264***	282
	Intergenic regions	HvDRR13	823	840
		HvDRR27	684***	486
		HvDRR28	1057	1031

*** *P* < 0.001 for *H*_0_: *μ*_Observed_ ≤ *μ*_Random_; Z‐test.

#### The variation in methylation and SVs in coldspot and hotspot windows among barley populations

Significant differences (*P* < 0.016) were observed among the three analyzed populations in the methylation levels of the CpG and CHG sequence contexts in all windows but also in coldspot windows (Figure 3). By contrast, the methylation level in the hotspot windows was found to be equal (*P* > 0.016) among populations in any of the analyzed chromosome regions. A similar trend was observed for the total SV load fraction: While observing significant differences (*P* < 0.016) among populations in all windows and coldspot windows in both the pericentromeric region and the distal subregions, such differences were not observed for the hotspot windows.

The methylation level of the two parental inbred lines of each of the analyzed populations differed significantly (*P* < 0.008) at the CpG and CHG sequence contexts of the genomic windows identified as coldspots in their respective offspring (Table 2). Thus, the increased methylation of only one of the parental genotypes at a genomic region might be enough to generate a coldspot in the offspring. In hotspot windows, no significant differences (*P* > 0.008) between parental inbred lines were found in the methylation level at either of the two analyzed sequence contexts, indicating that parents must have equally low methylation at a genomic segment to allow a recombination hotspot.

**Table 2:** Comparison of the methylation level at sequence contexts CpG and CHG in the 10 kb windows identified as recombination hotspots and coldspots in the double round‐robin (DRR) populations HvDRR13, HvDRR27, and HvDRR28 for the respective parental inbred lines. The coldspot and hotspot windows were calculated on the basis of the recombination rate of the DRR populations. For a given population and given methylated sequence context, the significant difference (α = 0.016) in the Wilcoxon's rank‐sum test among genomic window groups is indicated with different letters

Population	Coldspot windows				Hotspot windows			
	Offspring	HOR8160	Unumli‐Arpa	SprattArcher	Offspring	HOR8160	Unumli‐Arpa	SprattArcher
HvDRR13								
CpG	0.880 c	0.875 b	–	0.883 a	0.666 a	0.661 a	–	0.668 a
CHG	0.581 c	0.571 b	–	0.590 a	0.334 a	0.322 a	–	0.345 a
HvDRR27								
CpG	0.894 b	–	0.891 a	0.892 b	0.673 a	–	0.667 a	0.678 a
CHG	0.595 c	–	0.587 a	0.602 b	0.346 a	–	0.330 a	0.362 a
HvDRR28								
CpG	0.880 c	0.875 b	0.881 a	–	0.645 a	0.643 a	0.644 a	–
CHG	0.573 c	0.570 b	0.574 a	–	0.319 a	0.319 a	0.315 a	–

#### DNA motif abundance in hotspot windows

We investigated the abundance of DNA sequence motifs previously associated with crossover regions in *Arabidopsis thaliana* within the barley CO hotspot windows. Motif abundance was compared between 10 kb hotspot windows and matched background windows separately for distal and pericentromeric regions. PolyA/T motifs were significantly enriched in hotspot windows in both regions, with approximately 23 and 19% higher abundance in distal and pericentromeric regions, respectively. By contrast, CTT/GAA and CT/GA motifs were significantly depleted in hotspot windows in both regions. CTT/GAA abundance was approximately 8% lower in hotspot windows, while CT/GA abundance was reduced by approximately 5% in distal regions and 6% in pericentromeric regions. No significant differences in CCN/GGN motif abundance were detected between hotspot and background windows in either genomic region (Table S16, Figures S12 and S13). Overall, these results indicate that the association between crossover hotspots and previously reported crossover‐associated motifs is not uniform, with PolyA/T showing consistent enrichment, whereas CTT/GAA and CT/GA were less abundant within hotspot regions.

## DISCUSSION

### Detection of recombination events at high resolution in barley

In the present study, COs were detected at a high resolution of 10 kb in three randomly selected but connected barley DRR populations. This is a high resolution compared to other pedigree‐based studies as previous studies with a higher resolution (1–2 kb) were population‐based studies that inferred COs from ancestral LD (Choi et al., 2013; Marand et al., 2019). The substantial decrease of the median inter‐SNP distance compared to a previous study with the same three populations (Casale et al., 2022) produced a slight increase of the CO discovery rate of 0.31 times when considering the COs related to > 3 Mbp marker blocks. However, such increase jumps to 2.74 and 10.59 times if considering the COs related to > 500 kb and > 10 kb blocks, respectively (Tables S3 and S5). The part of the observed increase which is due to the additional recombination that occurred at heterozygous regions in the selfing generation analyzed in the first study is expected to be small due to the decreasing remaining heterozygosity after every selfing generation that produces fewer new observable COs per generation during inbreeding (Esch et al., 2007). Instead, our finding illustrates the added value of increasing the genotyping intensity. In similar comparisons between low‐ and high‐resolution analyses reported in previous studies, only small differences were detected in populations of *Arabidopsis thaliana* and maize (*Zea mays*) (McMullen et al., 2009; Rodgers‐Melnick et al., 2015). However, substantial differences were reported in populations of wheat (Esch et al., 2007; Gardiner et al., 2019; Gutierrez‐Gonzalez et al., 2019) and Populus (Apuli et al., 2020). In addition to the different utilized resolutions, other reasons behind observing differences in the recombination rate in the same population may include the genotyping error rate, the data filtering criteria, and the size of considered CO events.

The observed CO rate per RIL per chromosome per generation in the present study, when considering the COs related to >3 Mbp marker blocks, is in line with high‐resolution studies in Arabidopsis (Lu et al., 2012; Qi et al., 2014; Rowan et al., 2019; Sun et al., 2012; Wijnker et al., 2013; Yang et al., 2012) and rice (*Oryza sativa*) (Si et al., 2015) that reported rates of 1.5–2.2 COs per chromosome per generation, and with another high‐resolution study on wheat (Gardiner et al., 2019) when looking at the COs related to >500 kb marker blocks.

The present study is the first to characterize GC events in barley, along with a few in other crop species (Gardiner et al., 2019; Li et al., 2015; Si et al., 2015). The poor documentation of GCs in plants beyond studies in Arabidopsis is because phenotypic screens can barely detect GC events. In addition, the detection of GC events at the molecular genetic level is also challenging because of their short length (Mancera et al., 2009; Mercier et al., 2015). This makes GC detection very sensitive to the marker density and GC rate, which also depend on the recombination rate, the tract length of the repair intermediates where GCs occur, and the polymorphism density (Wijnker et al., 2013). Moreover, in most flowering plants, gametes do not remain grouped after meiosis, making it difficult to observe the expected 3:1 allelic proportion of GCs (Sun et al., 2012). In the present study, the average number of genome‐wide GC events per RIL across populations was 58, 251, and 6,521 for long (2–10 kb), medium (20 bp–2 kb), and short (2–20 bp) GC‐related marker block lengths, respectively. The SNPs analyzed per population could be translated to 0.00003, 0.00014, and 0.0039 GC events per site per RIL per generation for the different types of GC‐related marker block lengths. Marker blocks shorter than 20 bp are expected to contain a high number of false‐positive GCs since they were predominantly called based on two markers only. Therefore, if considering the GC‐related marker blocks of long (2–10 kb) and medium (20 bp–2 kb) length only, the detected 0.00017 GCs per site per RIL per generation is in the same order of magnitude as that observed in studies using a similar approach for GC detection (Gardiner et al., 2019; Yang et al., 2012). However, the observed GC rate in our study was two orders of magnitude greater than that reported in tetrad analysis studies performed in Arabidopsis (Lu et al., 2012; Sun et al., 2012; Wijnker et al., 2013) and sequencing of rice F2 populations (Si et al., 2015). This disagreement might be explained not only by the less precise GC detection method used in our study but also by the occurrence of nonallelic sequence alignments caused by SVs inflating the number of false‐positive gene conversions (Qi et al., 2014; Si et al., 2015). It is also worth noting that the reported GC rate is the frequency of GCs generated from NCO and CO events combined. In the present study, we did not attempt to estimate the rate of NCO in barley because such are only traceable after gamete formation when they lead to GC, and it was not possible to precisely differentiate between CO and NCO conversion tracts with the employed marker resolution. In addition, the DSB rate in barley is not known enough to estimate the NCO rate from the detected CO events.

The present study is the first to report genome‐wide CO hotspots at high resolution in barley. On average, across the three investigated populations, of the 80 hotspots per chromosome, only 12 were found in the pericentromeric region, and the rest were found in the distal regions of the barley chromosomes (Figure S9 and Table S8). In addition, while the three investigated populations always shared one parent, the proportion of shared hotspots between two and three populations was 10% and 1%, respectively, of the total hotspots detected in a given population (Figure S10A). This observation is in good agreement with previous studies in Arabidopsis showing that recombination hotspots were cross‐specific (Salomé et al., 2012).

By contrast, in the case of the GC hotspot windows, the overlap among the three populations was more than 15% (Figure S11). Moreover, GC hotspots were found to have high overlap not only with GC hotspots of the same population but also with windows that are hotspots in other populations (Table S10). Thus, GC hotspots might be considered fingerprints of population‐specific silenced COs that result in NCO in regions with high DSB rates in the genome of the species. This observation suggested that although the CO rate and distribution present extensive intraspecies variation, such DSBs might be highly conserved within the species. However, this requires further research.

Additionally, in barley, recombination hotspots alternate in the genome with coldspots, as observed in previous studies in other species (Mercier et al., 2015). Indeed, by dividing the genome into 10 kb genomic windows, hotspot windows were found to be adjacent to coldspot windows in 42.5% of the cases in the distal regions of the barley chromosomes (Table S15). This continuous intermittence in the recombination rate might explain the above‐mentioned large difference in CO events found between the high‐ and low‐resolution analyses on the barley populations under study.

To avoid calling recombination coldspots in the pericentromeric and telomeric regions of the chromosomes, which are long regions depleted from recombination as seen in previous studies (Boideau et al., 2022), in the present study, coldspots were identified only in the distal region of the chromosomes by employing a long physical distance margin between regions. Although large IBD regions with maximum inter‐SNP distances of 5.45–11.99 Mb may limit crossover detection, the median inter‐SNP distance was only 132 bp across populations, and the majority of detected 10‐kb coldspot windows occurred within longer regions averaging 322 kb (Table S7), indicating that coldspots were not predominantly restricted to isolated regions of limited marker availability (Table S3). Furthermore, each population shared 60% of its coldspot windows and more than 16% with the other two populations, thus demonstrating a greater conservation of coldspots than hotspots in barley (Figure S10B). Such differential conservation between recombination hotspots and coldspots might be linked to the different genomic features determining their occurrence. These distinct associations between the analyzed genomic features with hotspots and coldspots, respectively, support the notion that the regions identified as coldspots in our study are not solely attributable to undetected recombination events and may have biological relevance.

### The genomic features associated with recombination rate variation in barley

The present study is the first comprehensive evaluation of the genomic features associated with differences in recombination rates along the genome and among barley populations. In addition to the positive correlation shown between recombination and gene density on a broad scale, in the present study, 10 kb hotspot windows were found to be associated with regions of high gene density (Table 1), as repeatedly reported in previous studies in grasses (Bouchet et al., 2017; Casale et al., 2022; Darrier et al., 2017; Gardiner et al., 2019; Jordan et al., 2018; Marand et al., 2019; Rodgers‐Melnick et al., 2015), and other plant families (Apuli et al., 2020; Choi et al., 2013; Gion et al., 2016; Paape et al., 2012; Silva‐Junior & Grattapaglia, 2015; Wang et al., 2016). Furthermore, the hotspot windows were located in proximity (<20 kb apart) but did not overlap with the genes (Figure 4). This finding in barley is in line with previous observations in Arabidopsis, maize, and rice showing, with a higher resolution, an increased CO frequency toward gene promoters and terminators (Choi et al., 2013; Li et al., 2015; Marand et al., 2019; Sun et al., 2019; Wijnker et al., 2013), similar to that observed in budding yeast (*Saccharomyces cerevisiae*) (Pan et al., 2011). The proximity of hotspots to genes implies that relying on mRNA‐seq SNPs for CO detection might have inflated the count of hotspots over coldspots as genic regions were more covered than nongenic regions.

In the present study, the genetic effects were calculated as the proportion of the parental *GRE* not related to methylation, assuming parental sequence divergence and SV load as part of the *SRE* (Casale et al., 2022). Such genetic effects were shown to be the factor explaining the greatest proportion of differences in recombination rates among barley populations. Here, we hypothesize that such genetic effects are the effect of genes related to the recombination machinery, explaining a portion of the uneven distribution of the recombination hotspots along the barley chromosomes. The wide distribution of the observed genetic effects and methylation variations along the chromosomes is in line with previous studies in wheat (Jordan et al., 2018) and barley (Casale et al., 2022) reporting that differences in the genome recombination rate among populations are explained by multiple loci with small effects.

The results of the present study are in line with earlier studies in plants in which recombination was found to be positively associated with genetic divergence among homologous chromosomes (Blackwell et al., 2020; Marand et al., 2019; Yang et al., 2012). Although a negative association was reported in some studies (Bouchet et al., 2017; Gion et al., 2016; Gutierrez‐Gonzalez et al., 2019; Jordan et al., 2018; Saintenac et al., 2011; Serra et al., 2018), the contradiction might be explained by a sigmoid relationship between both variables, meaning that recombination has a positive correlation with genetic divergence until a level after which the high polymorphism among homologs suppresses COs due to the increase in mismatches, as recently reported in Arabidopsis (Blackwell et al., 2020; Hsu et al., 2022). In this respect, the few observed 1 Mbp windows with a significant negative association were found to have parental sequence divergence at the relative maximum level, which appeared to be associated with an extensive SV load (Figure S4A,B).

The SV load was not identified as a determining factor for the differences in the recombination rate among populations on a broad scale, presumably because the employed resolution of 1 Mbp was too coarse to detect differences among populations, as most of the analyzed SVs were smaller (Figure 2). The positive correlation between the recombination rate and the SV load along the genome on a broad scale (Figure 1A,C) might be explained by the accumulation of DNA repair errors in highly recombining regions throughout the evolutionary history of barley, as demonstrated in humans (Arbeithuber et al., 2015; Halldorsson et al., 2019). By contrast, at a fine scale, we were able to detect a decreased overlap and a longer distance between CO breakpoints and SVs compared to a random distribution, thus indicating the negative association of SVs with the occurrence of COs in barley (Tables S13 and S14). This is in line with previous high‐resolution studies in Arabidopsis (Fernandes et al., 2024; Rowan et al., 2019) and other plant species (Rodgers‐Melnick et al., 2015; Shen et al., 2019) where the presence of SVs was shown to suppress COs. Additionally, the type and size of the SVs were not found to be related to such effects, which is in line with previous findings in Arabidopsis (Rowan et al., 2019).

A negative correlation was observed on a broad scale between recombination rate and the extent of methylation in the CpG and CHG sequence contexts (Figure 1B). This is in accordance with previous studies in other plant species showing that COs occurred in euchromatic regions while heterochromatic regions were depleted of COs and that hypomethylation at CpG sites increased the genome‐wide CO rate (Apuli et al., 2020; Boideau et al., 2022; Colomé‐Tatché et al., 2012; Fernandes et al., 2024; Marand et al., 2019; Melamed‐Bessudo & Levy, 2012; Mirouze et al., 2012; Rodgers‐Melnick et al., 2015; Salomé et al., 2012; Wijnker et al., 2013; Yelina et al., 2012). This association was confirmed at high resolution by observing the relationship between 10 kb coldspot windows and increased methylation in the CpG and CHG sequence contexts (Figure 3). Furthermore, compared with those in the nonhotspot windows, the methylation levels in the CpG and CHG sequence contexts in both the distal and pericentromeric regions of the barley chromosomes decreased in the hotspot windows, in line with previous findings in maize (Rodgers‐Melnick et al., 2015).

In our study, the parental inbred lines of the analyzed populations were found to differ in the methylation level in the genomic windows identified as coldspots in their offspring populations (Table 2), indicating that the inheritance of high methylation from only one parent was sufficient to prevent recombination in a particular region of the genome. In a previous study on the same barley populations in which methylation was not separated from the genetic effects of genotypes, the effect of individual parental inbred lines was shown to be the major determinant of the recombination rate of the respective biparental offspring populations (Casale et al., 2022). The increased methylation at genomic regions leading to coldspots might be an important part of the effect of the parents on the recombination rate. By contrast, no significant differences between parental inbred lines were found in the methylation level of hotspot windows, indicating that the occurrence of a recombination hotspot is associated with equally low methylated parents at the respective genomic segment.

Moreover, this is the first study showing the joint effect of methylation and the accumulation of SVs in determining genomic regions deprived of recombination outside the pericentromeric region (Fernandes et al., 2024) and the variation in this effect within the genomic region (Figure 3 and Tables S11 and S12). In the distal telomeric region of the barley chromosomes, most 10 kb coldspot windows were found to be associated with increases in both the recombination level and the SV load. However, in the distal proximal region, increased methylation was found to be associated with most of the 10 kb coldspot windows, but an increased SV load was found to be associated with coldspot windows with no increased methylation. Interestingly, the effects of both methylation and SVs on the occurrence of coldspots were noticeable not only when comparing coldspot windows with other windows located far away in the same genomic region but also when comparing coldspots with their neighboring windows. This indicated a marked local effect of methylation and SVs on recombination suppression (Figure 4). In addition, the differences in both methylation level and SV load among barley populations were associated with the differences in the localization of coldspot windows among populations.

The enrichment of PolyA/T motifs in hotspot windows is consistent with their previously reported association with crossover regions in Arabidopsis (Rowan et al., 2019), suggesting that these sequence features may contribute to the formation or positioning of recombination hotspots across divergent plant species. By contrast, the depletion of CTT/GAA and CT/GA motifs in hotspot windows indicates that their association with recombination is not necessarily conserved across species. The absence of a significant association for CCN/GGN further suggests that the contribution of individual sequence motifs to crossover activity may be context dependent. Overall, these results support a contribution of local sequence composition to crossover landscape variation in barley, while indicating that the sequence features associated with recombination in Arabidopsis are not uniformly conserved.

The genomic features associated with differences in recombination rates among genetically diverse barley populations were identified at low resolution to then investigate their effects on local recombination variation at high resolution. Our findings agree with previous studies and illustrate that the recombination landscape in barley is highly predictable. In accordance with earlier findings, most of the recombination occurs in multiple short highly recombining sections in the distal regions of the chromosomes. Such recombination hotspots are located in proximity to genes and where the levels of methylation and SV load are low enough to allow CO concretion. The hotspots alternate with long regions deprived of recombination (coldspots) associated in part with increased methylation or the accumulation of SVs. These local differences in the recombination rate among barley populations are highly inheritable and can be determined by the effect of only one parent in a cross. The present analyses suggest that in addition to these two genomic features, other differences in the recombination machinery must exist, which form the basis for what we designated genetic effect.

## MATERIALS AND METHODS

### Identification of the genomic features associated with recombination rate variation among barley populations

The identification of the genomic features associated with recombination rate variation among barley populations was firstly performed at a low resolution (1 Mbp) to take advantage of previously generated data covering a wide range of the genetic diversity of cultivated barley. This part of the study utilized the entire barley double round‐robin populations (DRR), which consist of 45 biparental populations generated by crossing 23 parental inbreds following a DRR design, previously described by Casale et al. (2022). Parental inbreds were selected from a diversity panel to maximize genotypic and phenotypic richness (Weisweiler et al., 2019). The recombination rates of the DRR populations have been derived from genotyping the populations with the 50K Illumina Infinium iSelect SNP genotyping array (Bayer et al., 2017) as described in detail by Casale et al. (2022). The recombination rates were recalculated based on the Morex v3 reference genome sequence (Mascher et al., 2021) at 1 Mbp genomic windows. Structural variations among the original parental inbreds are assumed to average out on a genome‐wide basis in comparison to Morex; thus, the physical length of a genomic window of Morex was used when calculating the recombination rate of each population. The pericentromeric region of each chromosome was defined as the continuous region surrounding the centromere for which the average recombination rate across the 45 DRR populations was 5‐fold lower than the respective chromosome average across populations in 1 Mbp genomic windows. The non‐pericentromeric regions were designated in the following as distal regions.

Whole‐genome bisulfite DNA sequencing data for the 23 DRR parental inbred lines was obtained by extracting DNA from inbred lines from a mix of tissues, including the whole seedling plant, the leaf, and the apex, at stage 47 on the Zadoks scale. DNA library preparation was performed with NEBNext® Ultra™ II (New England Biolabs, Inc., Ipswich, MA, USA), and bisulfite conversion was performed with the EZ DNA Methylation‐Lightning Kit (Zymo Research, Irvine, CA, USA). The resulting 150 bp paired‐end libraries were sequenced with Illumina HiSeq™ 2000 and NovaSeq™ (Illumina, Inc., San Diego, CA, USA). The raw reads were trimmed with Trim Galore! (Krueger et al., 2023), mapped against the Morex v3 reference genome with Bismark (Krueger & Andrews, 2011), and aligned with Bowtie 2 (Langmead & Salzberg, 2012). The Morex v3 reference genome was SNP corrected with known SNPs for each inbred to increase the mapping efficiency. For quality control, SNPs were called with Bis‐SNP (Liu et al., 2012) and filtered for shared genomics positions with single nucleotide variation (SNV) data generated by DNA sequencing of the respective inbred lines (Weisweiler et al., 2022), as described in detail by Kühl et al. (2026). Pooling accuracy was evaluated by comparing the proportion of methylated reads between the mixed sample and the weighted average across the three separate tissue samples of the inbred line Sissy. Bisulfite conversion rates were assessed using standard protocols (Schmitz et al., 2021). The level of methylation at cytosine positions present in the sequence contexts CpG, CHG, and CHH, was calculated as the percentage of the methylated reads per position. For each DRR population, the methylation level at each sequence context in a given genomic window was calculated as the average among the respective parental inbred lines' methylation level values for the methylated cytosine positions in that window, weighted by the number of methylated cytosine positions available for each parent.

The gene density in 1 Mbp windows was calculated as the physical fraction spanned by the coding sequence of genes in each window. The locations of genes and intergenic regions on the barley chromosomes were obtained from the Morex v3 reference sequence. The genetic divergence among parents of the DRR populations was calculated from single nucleotide variant (SNV) data derived from genome‐wide sequencing (Weisweiler et al., 2022).

The SNVs were also used to calculate the general recombination effects (*GRE*) of the parental inbred lines from the pattern of recombination frequencies observed across all 45 populations by using a mixed‐model framework, described in detail by Casale et al. (2022). The GRE is the individual effect of a given parent on recombination that occurs in its progeny regardless of the other parents involved. In the next step, the proportion of the sum of the GRE of both parents involved in a given population that was not explained by the average methylation in each 1 Mbp window was estimated using a linear regression. The residual of this analysis was assumed to represent the genetic effects on recombination in a given genomic window. The specific recombination effect (SRE) for a given parental combination was not taken into account to estimate the genetic effects because it was previously described to cause only a minor effect on the recombination rate of a given biparental barley population (Casale et al., 2022).

The SVs, such as inversions, insertions, deletions, duplications, and translocations, between the parental inbred lines of the DRR populations and the Morex reference genome were obtained from Weisweiler et al. (2022). On a population basis, a structural variant was determined when a structural change with respect to the Morex reference was present in one parent but not in the other. The SVs were categorized by size (50–299 bp, 0.3–4.9 kb, 5–49 kb, 50–249 kb, 0.25–1 Mbp, and >1 Mbp), except for translocations whose length was not determined (Table S1). The physical length fraction spanned by SVs in every 1 Mbp genomic window across the genome was estimated for all SV categories and sizes. Furthermore, the total SV span fraction generated by the sum of all SV categories and sizes in each 1 Mbp window was calculated (hereafter referred to as SV load).

Pearson's correlation between all pairs of the above‐mentioned genomic features was calculated by genomic window and population after normalization. To identify which of the features better explained the recombination rate variation among the 45 DRR populations at each 1 Mbp genomic window in the barley chromosomes, a stepwise regression approach was used. The procedure keeps for each 1 Mbp window the subset of genomic features that explain differences among the recombination rates of the DRR populations with the highest Akaike information criterion (AIC). The fraction of 1 Mbp windows of the barley genome in which a given genomic feature was retained in the model provides an estimation of the feature's importance across the entire genome. Moreover, the direction of the correlation between the analyzed features and the recombination rate provides a notion of the impact of the feature on either promoting or repressing recombination. The model included the total SV load, parental sequence divergence, genetic effects, and methylation level in the sequence contexts CpG, CHG, and CHH. In addition, an extended model was constructed by breaking down the SV load into the different SV types.

### Investigation of the genomic features associated with the recombination rate in barley at high resolution

#### Plant material, genotyping, and data cleaning

The detection of recombination events at a high resolution (10 kb) and the investigation of their associated genomic features were then performed in a reduced number of DRR populations. From the 45 DRR population set, three connected populations (HvDRR13, HvDRR27, and HvDRR28) were selected randomly for high‐density genotyping using an mRNA‐seq approach as described by Arlt et al. (2023). These populations are the product of a triangle cross among three parental inbred lines (HOR8160, SprattArcher, and Unumli‐Arpa). The respective 64, 92, and 79 RILs were cultivated at the S7 generation in petri dishes in a randomized incomplete block design where the parental inbred lines were included as controls. The blocks were harvested 7 days after planting with less than 2 hours difference between the first and last sample. The whole seedling was utilized for mRNA extraction. For mRNA sequencing (RNA‐Seq), a RIL‐specific library was constructed using the VAHTS Universal V6 RNA‐seq Library Prep Kit for Illumina (Vazyme, China), and RIL‐specific barcodes were used. The pooled libraries were sequenced on the DNBSEQ‐G400 platform (MGI Tech Co., Ltd., Shenzhen, GD, China) by BGI Genomics (Beijing, China), generating 1.42 billion 150 bp paired‐end reads. The reads were trimmed with Trimmomatic (Bolger et al., 2014) and aligned to the Morex v3 reference sequence using HISAT2 (Kim et al., 2019). Variant calling was performed using BCFtools (Li et al., 2009).

The obtained variants were selected based on their intersection with the reported SNVs from the parental inbred lines (Weisweiler et al., 2022). An additional 12 K subset from the SNP array mentioned above was added to the total set at genomic positions not present in the RNA‐seq dataset. On a population basis, SNPs carrying nonparental alleles were set to missing data, and SNPs with 100% missing data or monomorphic parental alleles were discarded. Missing data for genotypes at polymorphic positions were imputed on a population level using Beagle (Browning et al., 2018) with default parameters.

#### Detection of recombination events

A recombination event in a given RIL haplotype was called when a group of contiguous SNPs which alleles were inherited from the same parental inbred (in the following designated as SNP block) switched to a SNP block inherited from the other parent (i.e., parental allele phase change). The recombination breakpoints were determined as the midpoint of the interval between both blocks (i.e., the CO interval). The blocks comprising fewer than three SNPs were considered false‐positive CO events and were discarded. Then, blocks with a parental allele phase change shorter than 10 kb were considered GC events, while blocks with a parental allele phase change longer than 10 kb were considered to be produced by CO (Gardiner et al., 2019; Yang et al., 2012). To enable comparisons with earlier studies (Gardiner et al., 2019; Yang et al., 2012), GC events which result were designated in the following as GC blocks were grouped into the category long (2–10 kb), short (20 bp–2 kb), and very short (2–19 bp). Similarly, CO events which result were designated in the following as CO blocks were grouped into the category short (10–500 kb), medium (500 kb–3 Mbp), and long (>3 Mbp) blocks. The longest CO block length threshold that kept every major parental allele phase change (3 Mbp) was defined visually by graphical genotypes on a 500 kb scale from 0.5 to 20 Mbp (Figure S2). The CO block length categories were considered to be different CO layers, and further analyses were performed on a multilayer basis. Individuals with a CO count falling outside the 3‐fold interquartile range of their respective population were assumed to be outliers and were discarded from further analyses.

The pericentromeric and distal regions of each chromosome were defined as explained above at 10 kb genomic windows for each of the three analyzed populations independently. The distal regions were defined as the chromosome segments between the pericentromeric region and the telomeres of the chromosomes.

In each 10 kb genomic window of the genome, the physical fraction spanned by the COs determined in all RILs of a population was aggregated to calculate the accumulated CO probability per window on a population basis. The accumulated CO probabilities per window were normalized per chromosome and per population. The CO hotspot windows in a given population were defined as the windows with a CO probability within the top 0.01 percentile among the windows with a CO probability above the chromosome average per population. The GC hotspot windows were determined in the same way as the CO hotspot windows. The windows located in the distal regions with a CO probability of zero were considered coldspot windows. The coldspot windows that were located beside other coldspot windows were combined into coldspot regions. To avoid calling coldspot windows near the pericentromeric region and telomeres, only windows located away from such regions were considered coldspot windows. This distance was granted by introducing an arbitrary margin with a length of 12.5% of the physical length of the respective distal region. To control for spurious associations generated by the variation of the features along the chromosomes, the windows from the distal regions were grouped into those close to the telomere (distal telomeric) and those close to the pericentromeric region (distal proximal).

#### Investigation of the association between genomic features and recombination rate

The fraction of each window spanned by SVs, the gene density, the methylation level at the sequence contexts CpG and CHG (derived from the parental measurements described above), and the parental difference for these contexts were calculated as described above for each 10 kb genomic window of the barley chromosomes in the three analyzed populations HvDRR13, HvDRR27, and HvDRR28.

In addition, the 10 kb windows neighboring coldspot and hotspot regions which were defined above were grouped by their relative position in the range from −40 kb to +40 kb around the respective coldspot or hotspot. The statistical comparison among window groups of any kind for the mentioned features was performed with the Mann–Whitney *U* test with Bonferroni correction for multiple testing.

The observed overlaps of the coldspot and hotspot regions with genes and intergenic regions of the three analyzed populations were statistically compared with random overlaps simulated with regioneR (Gel et al., 2016) by running 1,000 permutations on a by‐chromosome basis where the respective pericentromeric regions were masked.

The observed overlap between CO intervals and the genomic regions spanned by SVs was assessed as described above with regioneR for insertions/deletions, duplications, and inversions. In addition, the mean distance between the CO breakpoints and their closest SV in the genome was compared with the equivalent of random simulated COs in 10 Mbp genomic windows, each with a probability of CO occurrence according to the recombination rate per chromosome in the DRR populations reported by Casale et al. (2022). The significant differences among the means of the observed and simulated CO‐SV distances were evaluated with the Mann–Whitney *U* test.

For comparison between CO hotspot windows and matched background windows, 10‐kb background windows were randomly sampled from the same chromosome and genomic region (Distal or Pericentromeric) as each hotspot window, excluding windows containing CO hotspots. Ten background windows were sampled per hotspot window. Sequences were extracted from the Morex V3 reference genome, and the abundance of four motif classes previously associated with crossover regions in Arabidopsis (Rowan et al., 2019; Wijnker et al., 2013)—PolyA/T, CTT/GAA, CT/GA, and CCN/GGN—was quantified as the total number of motif occurrences within each 10‐kb window. Motif counts were compared between hotspot and background windows separately for distal and pericentromeric regions using two‐sided Wilcoxon rank‐sum tests. Enrichment was calculated as the ratio of mean motif counts in hotspot windows to those in background windows.

## AUTHOR CONTRIBUTIONS

FC contributed to the design of the project, analyzed the data, and performed the analyses related to meiotic recombination, and performed all statistical analyses. CA created the libraries, sequenced, and processed the RNA‐Seq data. MK analyzed the bisulfite‐sequenced DNA data. Both CA and MK contributes equally to this work as second authors. JL created the segregating populations. JE and TH created the bisulfite libraries for DNA sequencing. BS designed and coordinated the project. FC and BS wrote the manuscript. All authors read and approved the final manuscript.

## CONFLICT OF INTEREST

The authors declare that they have no competing interest.

## Supporting information


**Table S1.** Number of structural variants (SV) segregating in the analyzed double round‐robin (DRR) populations HvDRR13 HvDRR27, and HvDRR28, classified by type (inversions, deletions, insertions, duplications, and translocations) and size (50–299 bp, 0.3–4.9 kb, 5–49 kb, 50–249 kb, 0.25–1 Mbp, and >1 Mbp) on average per recombinant inbred line (RIL).
**Table S2.** The fraction of 1 Mbp windows of the barley genome in which the genomic features were found to be significantly associated with the recombination rate variation across the 45 double round‐robin (DRR) populations. The analysis was performed separately for the distal and pericentromeric regions of the barley chromosomes, respectively. The studied genetic features included parental sequence divergence, genetic effects, and span fraction of structural variants (SV) such as insertions, deletions, inversions, and duplications. The epigenetic features included the methylated level for the sequence contexts CpG, CHG, and CHH. The significant correlations between the recombination rate and the mentioned features were determined by a stepwise regression procedure. The fraction of 1 Mbp windows positively and negatively associated with the recombination rate were indicated with the “+” or the “−” signs, respectively.
**Table S3.** The quality metrics of the filtered high‐density RNA‐seq single nucleotide polymorphism (SNP) markers in the analyzed double round‐robin (DRR) populations HvDRR13, HvDRR27, and HvDRR28.
**Table S4.** The central tendency metrics of the length (bp) and the number of markers per block (N markers/block) of the crossover (CO)‐related parental marker blocks. The marker blocks are presented for three different thresholds (>10 kb, >500 kb, and >3 Mbp) on average per recombinant inbred lines (RIL) in the analyzed double round‐robin (DRR) populations HvDRR13, HvDRR27, and HvDRR28. Pearson's correlation coefficient (r) between block length and N markers per block is indicated as well as the respective significance.
**Table S5.** The summary of the crossover (CO) and gene conversion (GC) events classified by their related marker block size (COs: >10 kb, >500 kb, and >3 Mbp; GCs: 2–20 bp, 20 bp–2 kb, and 2–10 kb, respectively) on average per recombinant inbred line (RIL) in the analyzed double round‐robin (DRR) populations HvDRR13, HvDRR27, and HvDRR28.
**Table S6.** The central tendency measures for crossover (CO) intervals' length classified by related marker block size (>10 kb, >500 kb, and >3 Mbp) on average per recombinant inbred lines (RIL) of the analyzed double round‐robin (DRR) populations HvDRR13, HvDRR27, and HvDRR28.
**Table S7.** The number of 10 kb windows corresponding to recombination coldspots and hotspots, and the number of coldspot regions in the barley chromosomes in the analyzed double round‐robin (DRR) populations HvDRR13, HvDRR27, and HvDRR28.
**Table S8.** The number of 10 kb windows corresponding to the total, coldspots, and hotspots in the pericentromeric, distal proximal, and distal telomeric regions of the chromosomes in the analyzed double round‐robin (DRR) populations HvDRR13, HvDRR27, and HvDRR28.
**Table S9.** The observed overlaps among the gene conversion (GC) hotspot and crossover (CO) coldspot and hotspot 10 kb windows in the distal region of the barley chromosomes, and their statistical comparison with the overlaps generated under a random distribution of such regions in the analyzed double round‐robin (DRR) populations HvDRR13, HvDRR27, and HvDRR28. The random distribution of the genomic regions was simulated with 1000 permutations.
**Table S10.** The proportion of gene conversion (GC) hotspots of 10 kb windows overlapping with crossover (CO) hotspots, their neighboring windows at 10 and 20 kb away, CO hotspots in the two other studied populations, and the rest of the windows in the pericentromeric and the distal regions of the chromosomes in the analyzed double round‐robin (DRR) populations HvDRR13, HvDRR27, and HvDRR28. For a given population, the significant difference (α = 0.0083) in the Chi‐squared test among the overlap of two given window regions with the GC hotspot windows are indicated with different letters.
**Table S11.** Total structural variants (SV) load fraction of the coldspot 10 kb windows in the distal telomeric region of the barley chromosomes which were below and above the critical value in the comparison of the methylation level between such coldspots and the total genomic windows of the distal proximal region of the chromosomes for the methylated sequence contexts CpG and CHG in the three analyzed populations HvDRR13, HvDRR27, and HvDRR28.
**Table S12.** Methylation level of the coldspot 10 kb genomic windows in the distal proximal region of the barley chromosomes which were below and above the critical value in the comparison of the total structural variants (SV) load fraction between such coldspots and the total windows in their respective subregion in the chromosomes of the three analyzed populations HvDRR13, HvDRR27, and HvDRR28.
**Table S13.** Observed overlaps among the crossover (CO) intervals (related to >3 Mbp marker blocks) and the genomic regions spanned by structural variants (SV) along the distal regions of the barley chromosomes, and their statistical comparison with the overlaps generated under a random distribution of such regions in the double round‐robin (DRR) populations HvDRR13 HvDRR27, and HvDRR28. The SVs were classified by type (inversions, deletions, insertions, duplications, and translocations). The random distribution of the genomic regions was simulated with 1000 permutations.
**Table S14.** Physical distance between crossover (CO) breakpoints and their closest structural variant (SV) compared with the same distance for simulated COs across the genome of the three analyzed populations HvDRR13, HvDRR27, and HvDRR28. The SVs were classified by type (inversions, deletions, insertions, duplications, and translocations) and size (50–299 bp, 0.3–4.9 kb, 5–49 kb, 50–249 kb, 0.25–1 Mbp, and >1 Mbp).
**Table S15.** Proportion of either hotspot or coldspot 10 kb genomic windows in the averaged neighboring windows located 10 kb upstream or downstream of the hotspot and coldspot genomic regions in the distal proximal and telomeric subregions of the barley chromosomes in the three analyzed populations HvDRR13, HvDRR27, and HvDRR28.
**Table S16.** Comparison of motif abundance between CO hotspot and matched background windows. Mean counts of four previously reported crossover‐associated DNA motifs within 10‐kb CO hotspot windows and matched 10‐kb background windows, shown separately for distal and pericentromeric genomic regions. CO hotspots were identified independently in each population (HvDRR13, HvDRR27, and HvDRR28), and 10‐kb windows overlapping hotspots in at least two populations were selected as CO hotspot windows for subsequent analyses. Fold change represents the ratio of mean motif counts in hotspot windows to mean motif counts in background windows. Statistical significance was assessed using a two‐sided Wilcoxon rank‐sum test. Motif classes comprise PolyA/T, CTT/GAA, CT/GA, and CCN/GGN.
**Figure S1.** Distribution of recombination rate and structural variants (SV): insertions, deletions, inversions, and duplications, across the seven barley chromosomes. The displayed values represent the spanned fraction of a 1 Mbp genomic window by the sum of all SVs of the same kind. The values were averaged across the 45 double round‐robin (DRR) populations in 1 Mbp windows across the seven barley chromosomes. The vertical blue solid line indicates the position of the centromere in the Morex v3 reference genome, and the vertical blue dashed lines indicate the pericentromeric region calculated across the 45 DRR populations.
**Figure S2.** Example of the graphical genotype for one recombinant inbred line (RIL). The four graphical genotypes for each of the seven barley chromosomes show, from the raw data, the different three crossover (CO)‐related parental marker block categories considered in this study: 10–500 kb, 0.5–3 Mbp, and >3 Mbp, respectively.
**Figure S3.** Correlation matrix between recombination rate, the total structural variants (SV) load and such of the different types—insertions, deletions, inversions, and duplications—separately, the parental sequence divergence, gene density, and the methylation level at the sequence contexts CpG, CHG, and CHH, across 1 Mbp windows in the distal (A) and the pericentromeric (B) regions of the barley chromosomes for the average among the 45 double round‐robin (DRR) populations. Pearson's correlation coefficients are displayed and indicated with a color gradient from −1 (red) to 1 (blue).
**Figure S4.** Relative value of genomic features in the 1 Mbp genomic windows in which the explanatory feature was found significantly correlated with the variation in the recombination rate among the 45 double round‐robin (DRR) populations. The significance of the correlation was determined by a stepwise regression procedure. The studied genomic features included the recombination rate (light blue), sequence divergence among parental inbred lines (orange), total structural variants (SV) load (yellow), genetic effects (green), and methylation level in the sequence contexts CpG (red), CHG (maroon), and CHH (purple). The explanatory features are (A) sequence parental divergence, (B) total SV load, (C) genetic effects, (D, E, and F) methylation level in CpG, CHG, and CHH sequence contexts, respectively. The dots correspond to the relative value of the recombination rate.
**Figure S5.** Summary genomic features explaining the variation in the recombination rate among the 45 double round‐robin (DRR) populations in 1 Mbp windows across the seven barley chromosomes. The significance of the correlation was determined by a stepwise regression procedure. The standardized regression coefficients are indicated by a color gradient from −1 (purple) to 1 (red). The studied genomic features included sequence divergence among parental inbred lines, genetic effects, span fraction of structural variants (SV)—such as insertions, deletions, inversions, and duplications—and methylation level in the sequence contexts CpG, CHG, and CHH. The vertical solid line indicates the position of the centromere in the reference genome, and the vertical dashed lines indicate the pericentromeric region calculated across the 45 DRR populations.
**Figure S6.** Venn diagram for the number of polymorphic single nucleotide polymorphism (SNP) markers detected by RNA‐seq in the analyzed double round‐robin (DRR) populations HvDRR13, HvDRR27, and HvDRR28.
**Figure S7.** Stacked graphical genotypes of the recombinant inbred lines (RILs) for chromosome 6H in the HvDRR13 population. Different colors indicate parental allelic blocks inherited from different parents. The RILs are ordered according to their hierarchical complete clustering based on Euclidean distances of recombination rates for this chromosome. The vertical solid line in the background indicates the position of the centromere in the Morex v3 reference genome, and the vertical dashed lines indicate the pericentromeric region calculated across the 45 DRR populations.
**Figure S8.** Box plot of the number of counted genome‐wide crossovers (CO) with >3 Mbp marker blocks in the analyzed double round‐robin (DRR) populations HvDRR13, HvDRR27, and HvDRR28.
**Figure S9.** Normalized accumulated crossover (CO) probability (gray line), hotspot (red dots), and coldspot (turquoise triangles) 10 kb genomic windows across the seven barley chromosomes in the analyzed double round‐robin (DRR) populations HvDRR13, HvDRR27, and HvDRR28. The vertical solid line in the back‐ ground indicates the position of the centromere in the v3 Morex reference genome, and the vertical dashed lines indicate the pericentromeric region calculated for each chromosome and population.
**Figure S10.** Venn diagrams for the number of crossover (CO) hotspot (A) and coldspot (B) 10 kb windows in the analyzed double round‐robin (DRR) populations HvDRR13, HvDRR27, and HvDRR28.
**Figure S11.** Venn diagrams for the number of gene conversion (GC) hotspot 10 kb windows in the analyzed double round‐robin (DRR) populations HvDRR13, HvDRR27, and HvDRR28.
**Figure S12.** Motif abundance in CO hotspot and background genomic windows. Mean counts of four previously reported crossover‐associated DNA motifs within 10‐kb CO hotspot windows and matched 10‐kb background windows in distal and pericentromeric genomic regions of the barley genome. CO hotspots were identified independently in each population (HvDRR13, HvDRR27, and HvDRR28), and 10‐kb windows overlapping hotspots in at least two populations were selected as CO hotspot windows for subsequent analyses. Background windows were sampled from the same chromosome and genomic region as the corresponding hotspot windows.
**Figure S13.** Enrichment of DNA motifs in CO hotspot windows relative to matched genomic background. Fold enrichment of four previously reported crossover‐associated DNA motifs in 10‐kb CO hotspot windows relative to matched 10‐kb background windows in distal and pericentromeric genomic regions of the barley genome. CO hotspots were identified independently in each population (HvDRR13, HvDRR27, and HvDRR28), and 10‐kb windows overlapping hotspots in at least two populations were selected as CO hotspot windows for subsequent analyses. Enrichment was calculated as the ratio of mean motif counts in hotspot windows to mean motif counts in background windows. The dashed line at 1 indicates no enrichment or depletion. Motif classes comprise PolyA/T, CTT/GAA, CT/GA, and CCN/GGN.
**Figure S14.** Chromosome‐wide distribution of RNA‐seq‐derived SNPs across the seven barley chromosomes and three analyzed populations HvDRR13, HvDRR27, and HvDRR28. The number of SNPs was calculated in non‐overlapping 10‐kb windows along each chromosome for each population. SNP density is shown as the number of SNPs per 10‐kb window, with chromosomes arranged as rows and populations as columns. The y‐axis is constrained to 2500 SNPs per window for visualization.

## Data Availability

The methylation data of the 23 DRR population parental lines and the raw mRNA sequencing data of the RILs from populations HvDRR13, HvDRR27, and HvDRR28 (and their parental lines) are both available at NCBI, BioProject accessions PRJNA1100572 and 1088431, respectively. Employed scripts are available at https://github.com/fedecasale/barley_recombination_features or from the authors upon request.
