# Supplementary material for: The role of methylation and structural variants in shaping the recombination landscape of barley
Source: Plant J. 2026 Oct 11;128(1):e71171. doi: 10.1111/tpj.71171 (PMC13655489; doi:10.1111/tpj.71171)
Supplement: Supplementary file 1 [file TPJ-128-0-s001.docx]

Table S1: Number of structural variants (SV) segregating in the analyzed dou- ble round-robin (DRR) populations HvDRR13 HvDRR27, and HvDRR28, classified by type (inversions, deletions, insertions, duplications, and translo- cations) and size (50—299 bp, 0.3—4.9 kb, 5—49 kb, 50—249 kb, 0.25—1 Mbp, and *>*1 Mbp) on average per recombinant inbred line (RIL).

|  | | | Populations |  |
| --- | --- | --- | --- | --- |
| SV | SV size | HvDRR13 | HvDRR27 | HvDRR28 |
| inversions | 50—299 bp | 2.73 | 4.02 | 6.79 |
|  | 0.3—4.9 kb | 2.41 | 3.68 | 6.26 |
|  | 5—49 kb | 3.40 | 5.29 | 8.82 |
|  | 50—249 kb | 3.49 | 5.28 | 8.85 |
|  | 0.25—1 Mbp | 1.73 | 2.56 | 4.15 |
| deletions | 50—299 bp | 194.41 | 310.01 | 520.13 |
|  | 0.3—4.9 kb | 147.63 | 235.56 | 391.03 |
|  | 5—49 kb | 124.13 | 193.98 | 322.59 |
|  | 50—249 kb | 5.73 | 8.39 | 13.82 |
|  | 0.25—1 Mbp | 0.35 | 0.58 | 1.00 |
| insertions | 50—299 bp | 104.02 | 158.99 | 263.67 |
|  | 0.3—4.9 kb | 11.83 | 17.06 | 28.49 |
| duplications | 50—299 bp | 31.81 | 50.33 | 84.38 |
|  | 0.3—4.9 kb | 41.54 | 63.29 | 106.15 |
|  | 5—49 kb | 106.02 | 157.11 | 261.18 |
|  | 50—249 kb | 9.89 | 14.72 | 24.33 |
|  | 0.25—1 Mbp | 1.22 | 1.78 | 3.03 |
| translocations | - | 467.43 | 366.05 | 400.85 |

Table S2: The fraction of 1 Mbp windows of the barley genome in which the genomic features were found to be significantly associated with the recombination rate variation across the 45 double round-robin (DRR) populations. The analysis was performed sepa- rately for the distal and pericentromeric regions of the barley chromosomes, respectively. The studied genetic features included parental sequence divergence, genetic effects, and span fraction of structural variants (SV) such as insertions, deletions, inversions, and duplications. The epigenetic features included the methylated level for the sequence con- texts CpG, CHG, and CHH. The significant correlations between the recombination rate and the mentioned features were determined by a stepwise regression procedure. The fraction of 1 Mbp windows positively and negatively associated with the recombination rate were indicated with the ”+” or the ”−” signs, respectively.

Chromosome region

Genomic feature	Distal	Pericentromeric

|  | + | - |  | + | - |
| --- | --- | --- | --- | --- | --- |
| Extended model |  |  |  |  |  |
| Insertions | 0.05 | 0.03 |  | 0.03 | 0.02 |
| Deletions | 0.06 | 0.03 |  | 0.05 | 0.02 |
| Inversions | 0.02 | 0.01 |  | 0.01 | 0.01 |
| Duplications | 0.03 | 0.03 |  | 0.03 | 0.01 |
| Parental sequence divergence | 0.27 | 0.04 |  | 0.10 | 0.01 |
| Genetic effects | 0.70 | 0.04 |  | 0.27 | 0.04 |
| Methylation level: CpG | 0.03 | 0.21 |  | 0.03 | 0.02 |
| Methylation level: CHG | 0.01 | 0.14 |  | 0.02 | 0.01 |
| Methylation level: CHH | 0.01 | 0.18 |  | 0.02 | 0.01 |

Table S3: The quality metrics of the filtered high-density RNAseq single nucleotide polymorphism (SNP) markers in the analyzed double round-robin (DRR) populations HvDRR13, HvDRR27, and HvDRR28.

Population Molecular level No. markers	Density	Inter-SNP distance

|  | | | (mrk/Mbp) | Median (bp) | Mean (bp) | Max (Mbp) |  |
| --- | --- | --- | --- | --- | --- | --- | --- |
| HvDRR13 | Genome-wide | 214,112 | 51 | 135 | 19,587 | 11.99 |  |
|  | Chromosomes |  |  |  |  |  |  |
|  | 1H | 20,220 | 39 | 122 | 25,525 | 6.03 |  |
|  | 2H | 36,824 | 55 | 137 | 18,074 | 3.47 |  |
|  | 3H | 43,542 | 70 | 147 | 14,272 | 3.13 |  |
|  | 4H | 16,158 | 26 | 153 | 37,761 | 11.99 |  |
|  | 5H | 39,233 | 67 | 138 | 14,979 | 2.95 |  |
|  | 6H | 27,092 | 48 | 135 | 20,733 | 5.48 |  |
|  | 7H | 31,043 | 49 | 117 | 20,335 | 8.16 |  |
| HvDRR27 | Genome-wide | 259,540 | 62 | 130 | 16,162 | 5.45 |  |
|  | Chromosomes |  |  |  |  |  |  |
|  | 1H | 33,233 | 64 | 137 | 15,530 | 4.50 |  |
|  | 2H | 44,013 | 66 | 120 | 15,121 | 4.91 |  |
|  | 3H | 48,897 | 79 | 144 | 12,709 | 2.90 |  |
|  | 4H | 28,722 | 47 | 144 | 21,243 | 4.76 |  |
|  | 5H | 34,566 | 59 | 125 | 17,002 | 4.24 |  |
|  | 6H | 34,024 | 61 | 132 | 16,509 | 5.45 |  |
|  | 7H | 36,085 | 57 | 111 | 17,516 | 3.75 |  |
| HvDRR28 | Genome-wide | 244,353 | 58 | 132 | 17,167 | 7.17 |  |
|  | Chromosomes |  |  |  |  |  |  |
|  | 1H | 32,732 | 63 | 137 | 15,768 | 5.22 |  |
|  | 2H | 40,850 | 61 | 123 | 16,292 | 3.52 |  |
|  | 3H | 37,402 | 60 | 137 | 16,615 | 3.29 |  |
|  | 4H | 28,759 | 47 | 150 | 21,202 | 5.79 |  |
|  | 5H | 44,375 | 75 | 138 | 13,253 | 2.96 |  |
|  | 6H | 28,936 | 52 | 138 | 19,412 | 7.17 |  |
|  | 7H | 31,299 | 49 | 111 | 20,194 | 4.60 |  |

Table S4: The central tendency metrics of the length (bp) and the number of markers per block (N markers/block) of the crossover (CO) related parental marker blocks. The marker blocks are presented for three different thresholds (*>*10 kb, *>*500 kb, and *>*3 Mbp) on average per recombinant inbred lines (RIL) in the analyzed double round- robin (DRR) populations HvDRR13, HvDRR27, and HvDRR28. Pearson’s correlation coefficient (r) between block length and N markers per block is indicated as well as the respective significance.

CO layer	Block length (bp)	N markers/block	*r*	Significance

|  | median | mean | sd |  | median | mean | sd |  | |
| --- | --- | --- | --- | --- | --- | --- | --- | --- | --- |
| *>*3 Mbp | 27,540,588 | 111,572,731 | 171,044,519 |  | 3,282 | 5,962 | 7,339 | 0.84 | *** |
| *>*500 kb | 7,033,905 | 44,161,151 | 94,042,919 |  | 336 | 2,395 | 4,260 | 0.78 | *** |
| *>*10 kb | 886,623 | 14,799,456 | 48,144,287 |  | 50 | 817 | 2,024 | 0.75 | *** |
| ∗∗∗*P <* 0*.*001; Pearson’s correlation. |  |  |  |  |  |  |  |  |  |

Table S5: The summary of the crossover (CO) and gene conversion (GC) events classified by their related marker block size (COs: *>*10 kb, *>*500 kb, and *>*3 Mbp; GCs: 2—20 bp, 20 bp—2 kb, and 2—10 kb, respectively) on average per recombinant inbred line (RIL) in the analyzed double round-robin (DRR) populations HvDRR13, HvDRR27, and HvDRR28.

Population Molecular level	Crossovers (COs)	Gene conversions (GCs)

|  | | *>*3 Mbp | *>*500 kb | *>*10 kb |  | 2–10 kb | 20 bp–2 kb | 2–20 bp |
| --- | --- | --- | --- | --- | --- | --- | --- | --- |
| HvDRR13 | Genome-wide  Chromosomes 1H | 32  4.92 | 96  11.43 | 283  30.84 |  | 51  6.73 | 225  32.71 | 6,584  739 |
|  | 2H | 4.24 | 17.57 | 55.25 |  | 8.48 | 42.00 | 1,176 |
|  | 3H | 3.92 | 13.78 | 46.59 |  | 13.94 | 53.75 | 1,186 |
|  | 4H | 5.56 | 14.90 | 33.00 |  | 4.52 | 20.60 | 615 |
|  | 5H | 4.52 | 14.83 | 48.95 |  | 6.63 | 27.92 | 1,164 |
|  | 6H | 2.95 | 10.02 | 29.49 |  | 4.43 | 19.86 | 780 |
|  | 7H | 5.95 | 13.60 | 39.03 |  | 6.54 | 28.35 | 924 |
| HvDRR27 | Genome-wide Chromosomes 1H | 32.76  4.31 | 100.27  12.96 | 318.95  39.35 |  | 73.75  10.52 | 311.16  41.54 | 7,687  946 |
|  | 2H | 5.96 | 17.38 | 55.06 |  | 14.99 | 68.24 | 1,370 |
|  | 3H | 4.16 | 16.20 | 53.39 |  | 15.21 | 57.47 | 1,363 |
|  | 4H | 3.21 | 7.65 | 28.26 |  | 4.69 | 21.69 | 822 |
|  | 5H | 6.22 | 18.39 | 51.08 |  | 5.19 | 18.55 | 1,091 |
|  | 6H | 3.89 | 12.76 | 40.02 |  | 9.49 | 38.15 | 943 |
|  | 7H | 5.00 | 14.93 | 51.79 |  | 13.66 | 65.52 | 1,151 |
| HvDRR28 | Genome-wide Chromosomes 1H | 26.40  2.99 | 64.35  7.47 | 205.99  22.22 |  | 49.13  6.12 | 216.64  28.47 | 5,292  659 |
|  | 2H | 4.45 | 12.13 | 36.19 |  | 9.19 | 44.36 | 907 |
|  | 3H | 4.63 | 10.23 | 34.51 |  | 9.79 | 33.73 | 850 |
|  | 4H | 2.69 | 6.32 | 18.56 |  | 3.91 | 14.63 | 582 |
|  | 5H | 3.73 | 8.18 | 32.28 |  | 5.87 | 26.47 | 916 |
|  | 6H | 2.77 | 8.47 | 24.63 |  | 4.44 | 17.27 | 607 |
|  | 7H | 5.14 | 11.54 | 37.59 |  | 9.81 | 51.71 | 773 |

Table S6: The central tendency measures for crossover (CO) intervals’ length classified by re- lated marker block size (*>*10 kb, *>*500 kb, and

*>*3 Mbp) on average per recombinant inbred lines (RIL) of the analyzed double round-robin (DRR) populations HvDRR13, HvDRR27, and HvDRR28.

| CO layer | median | mean | sd |
| --- | --- | --- | --- |
| *>*3 Mbp | 151,632 | 695,585 | 1,455,871 |
| *>*500 kbp | 145,897 | 619,208 | 1,279,607 |
| *>*10 kbp | 41,959 | 345,952 | 893,055 |

Table S7: The number of 10 kb windows corresponding to recombination coldspots and hotspots, and the number of coldspot regions in the barley chro- mosomes in the analyzed double round-robin (DRR) populations HvDRR13, HvDRR27, and HvDRR28.

| Recombination region | Chromosome | HvDRR13 | HvDRR27 | HvDRR28 |
| --- | --- | --- | --- | --- |
| Hotspot windows | 1H | 45 | 40 | 27 |
|  | 2H | 82 | 65 | 73 |
|  | 3H | 83 | 73 | 80 |
|  | 4H | 42 | 26 | 53 |
|  | 5H | 53 | 56 | 76 |
|  | 6H | 41 | 36 | 45 |
|  | 7H | 57 | 42 | 80 |
|  | Genome-wide | 403 | 338 | 434 |
| Coldspot windows | 1H | 12355 | 10351 | 10847 |
|  | 2H | 9574 | 7387 | 11192 |
|  | 3H | 9099 | 9468 | 11388 |
|  | 4H | 14549 | 14213 | 15751 |
|  | 5H | 8254 | 9880 | 10340 |
|  | 6H | 8831 | 13829 | 9362 |
|  | 7H | 7051 | 8639 | 6804 |
|  | Genome-wide | 69713 | 73767 | 75684 |
| Coldspot regions | 1H | 179 | 329 | 310 |
|  | 2H | 313 | 484 | 385 |
|  | 3H | 335 | 420 | 389 |
|  | 4H | 207 | 341 | 287 |
|  | 5H | 357 | 437 | 404 |
|  | 6H | 224 | 379 | 204 |
|  | 7H | 280 | 353 | 313 |
|  | Genome-wide | 1895 | 2743 | 2292 |
| Average length (kb) | | 367 | 269 | 330 |
| Length range (kb) | | 10–17,580 | 10–12,200 | 10–12,010 |

Table S8: The number of 10 kb windows corresponding to the total, coldspots, and hotspots in the pericentromeric, distal proximal, and distal telomeric regions of the chromosomes in the analyzed double round-robin (DRR) populations HvDRR13, HvDRR27, and HvDRR28.

|  | | | Population |  |
| --- | --- | --- | --- | --- |
| Recombination region | Chromosome region | HvDRR13 | HvDRR27 | HvDRR28 |
| Total windows | Pericentromeric | 248993 | 220993 | 242993 |
|  | Distal proximal | 63990 | 74490 | 66240 |
|  | Distal telomeric | 63988 | 74488 | 66238 |
| Coldspot windows | Distal proximal | 39915 | 41844 | 43047 |
|  | Distal telomeric | 29798 | 31923 | 32637 |
| Hotspot windows | Pericentromeric | 66 | 81 | 100 |
|  | Distal proximal | 85 | 67 | 71 |
|  | Distal telomeric | 252 | 190 | 263 |

Table S9: The observed overlaps among the gene conversion (GC) hotspot and crossover (CO) coldspot and hotspot 10 kb windows in the distal region of the barley chromosomes, and their statistical comparison with the overlaps generated under a random distribution of such regions in the analyzed double round-robin (DRR) populations HvDRR13, HvDRR27, and HvDRR28. The random distribution of the genomic regions was simulated with 1,000 permutations.

| Recombination region | Population | Observed overlaps | Random overlaps |
| --- | --- | --- | --- |
| GC hotspot windows CO coldspot windows | HvDRR13 | 91 | 443 |
|  | HvDRR27 | 71 | 323 |
|  | HvDRR28 | 138 | 483 |
| CO hotspot windows | HvDRR13 | _54_∗∗∗ | 3 |
|  | HvDRR27 | _38_∗∗∗ | 1 |
|  | HvDRR28 | _60_∗∗∗ | 3 |

∗∗∗*P <* 0*.*001 for *H*_0_: *µ_Observed_* ≥ *µ_Random_*; Z-test.

Table S10: The proportion of gene conversion (GC) hotspots of 10 kb win- dows overlapping with crossover (CO) hotspots, their neighboring windows at 10 and 20 kb away, CO hotspots in the two other studied populations, and the rest of the windows in the pericentromeric and the distal regions of the chromosomes in the analyzed double round-robin (DRR) populations HvDRR13, HvDRR27, and HvDRR28. For a given population, the signif- icant difference (α = 0*.*0083) in the Chi-squared test among the overlap of two given window regions with the GC hotspot windows are indicated with different letters.

|  |  | Population |  |
| --- | --- | --- | --- |
| Window region | HvDRR13 | HvDRR27 | HvDRR28 |
| CO hotspot | 0.147 a | 0.122 a | 0.119 a |
| CO hotspot +10 kb neighbor | 0.019 b | 0.019 b | 0.025 b |
| CO hotspot +20 kb neighbor | 0.010 b | 0.013 b | 0.012 b |
| CO hotspot in the other populations | 0.075 c | 0.086 a | 0.095 a |
| Pericentromeric | 0.000 d | 0.000 c | 0.000 c |
| Distal | 0.002 e | 0.002 d | 0.002 d |

Table S11: Total structural variants (SV) load fraction of the coldspot 10 kb windows in the distal telomeric region of the barley chromosomes which were below and above the critical value in the comparison of the methylation level between such coldspots and the total genomic windows of the distal proximal region of the chromosomes for the methy- lated sequence contexts CpG and CHG in the three analyzed populations HvDRR13, HvDRR27, and HvDRR28.

| Population | CpG |  | CHG |
| --- | --- | --- | --- |
|  | Below critical Above critical |  | Below critical Above critical |
| HvDRR13 HvDRR27  HvDRR28 | 0.222 ^∗∗∗^ 0.181  0.235 ^∗∗∗^ 0.165  0.222 ^∗∗∗^ 0.178 |  | 0.229 ^∗∗∗^ 0.164  0.222 ^∗∗∗^ 0.158  0.224 ^∗∗∗^ 0.164 |
| ∗∗∗*P <* 0*.*001; Kruskal-Wallis-Test. |  |  |  |

Table S12: Methylation level of the coldspot 10 kb genomic windows in the distal proximal region of the barley chromosomes which were below and above the critical value in the comparison of the total structural variants (SV) load fraction between such coldspots and the total windows in their respective subregion in the chromosomes of the three analyzed populations HvDRR13, HvDRR27, and HvDRR28.

| Population | CpG |  | CHG |
| --- | --- | --- | --- |
|  | Below critical Above critical |  | Below critical Above critical |
| HvDRR13 HvDRR27  HvDRR28 | 0.898 ^∗∗∗^ 0.898  0.909 ^∗∗∗^ 0.902  0.901 ^∗∗∗^ 0.897 |  | 0.604 ^∗∗∗^ 0.602  0.615 ^∗∗∗^ 0.609  0.600 ^∗∗∗^ 0.593 |
| ∗∗∗*P <* 0*.*001; Kruskal-Wallis-Test. |  |  |  |

Table S13: Observed overlaps among the crossover (CO) intervals (related to *>*3 Mbp marker blocks) and the genomic regions spanned by structural variants (SV) along the distal regions of the barley chromosomes, and their statistical comparison with the overlaps generated under a random distribution of such regions in the dou- ble round-robin (DRR) populations HvDRR13 HvDRR27, and Hv- DRR28. The SVs were classified by type (inversions, deletions, in- sertions, duplications and translocations). The random distribution of the genomic regions was simulated with 1,000 permutations.

|  | | | Population |  |
| --- | --- | --- | --- | --- |
| SV type |  | HvDRR13 | HvDRR27 | HvDRR28 |
|  | Total CO intervals | 1692 | 2371 | 1891 |
| Deletions | Total SVs  Obs. overlaps | 773  3670^∗∗∗^ | 808  4663^∗∗∗^ | 841  4576^∗∗∗^ |
|  | Perm. overlaps | 7705 | 13911 | 9616 |
| Duplications | Total SVs  Obs. overlaps | 773  2249^∗∗∗^ | 808  2693^∗∗∗^ | 841  2447^∗∗∗^ |
|  | Perm. overlaps | 2866 | 4583 | 3382 |
| Insertions | Total SVs  Obs. overlaps | 773  1141^∗∗∗^ | 808  1428^∗∗∗^ | 841  1287^∗∗∗^ |
|  | Perm. overlaps | 1852 | 3139 | 2206 |
| Inversions | Total SVs | 773 | 808 | 841 |

Obs. overlaps	262	257^∗∗∗^	297

Perm. overlaps	282	441	330

∗∗∗*P <* 0*.*001 for *H*_0_: *µ_Observed_* ≥ *µ_Random_*; Z-test.

Table S14: Physical distance between crossover (CO) breakpoints and their closest structural variant (SV) compared with the same distance for simulated COs across the genome of the three analyzed populations HvDRR13, HvDRR27, and HvDRR28. The SVs were classified by type (inversions, deletions, insertions, duplications, and translocations) and size (50—299 bp, 0.3—4.9 kb, 5—49 kb, 50—249 kb, 0.25—1 Mbp, and

*>*1 Mbp).

| SV type | SV size | Observed distance |  |  | Simulated random distance |
| --- | --- | --- | --- | --- | --- |
|  |  | mean median | sd |  | mean median sd |

| Inversions | 50—299 bp | 17,099,530^∗∗∗^ 6,671,607 | 27,149,096 | 7,555,888 | 4,082,929 | 9,701,340 |
| --- | --- | --- | --- | --- | --- | --- |
|  | 0.3—4.9 kb | 17,214,528^∗∗∗^ 7,919,230 | 25,013,784 | 8,350,472 | 4,881,894 | 9,698,011 |
|  | 5—49 kb | 13,582,916^∗∗∗^ 7,082,586 | 17,761,643 | 7,568,365 | 4,025,608 | 10,906,124 |
|  | 50—249 kb | 15,582,683^∗∗∗^ 5,935,824 | 23,080,540 | 7,183,728 | 2,968,177 | 11,953,339 |
|  | 0.25—1 Mbp | 30,009,390^∗∗∗^13,862,023 | 51,763,750 | 22,406,932 | 7,977,245 | 52,897,364 |
| Deletions | 50—299 bp | 544,971^∗∗∗^ 137,030 | 1,205,795 | 232,554 | 83,471 | 478,947 |
|  | 0.3—4.9 kb | 804,234^∗∗∗^ 189,082 | 1,754,699 | 306,130 | 101,326 | 677,696 |
|  | 5—49 kb | 1,231,131^∗∗∗^ 190,110 | 3,546,213 | 337,269 | 107,321 | 996,528 |
|  | 50—249 kb | 12,709,060^∗∗∗^ 3,625,545 | 25,218,392 | 3,985,361 | 1,875,750 | 6,368,258 |
| Insertions | 50—299 bp | 1,046,414^∗∗∗^ 206,000 | 2,882,087 | 358,385 | 140,706 | 826,689 |
|  | 0.3—4.9 kb | 6,830,746^∗∗∗^ 1,578,779 | 16,618,799 | 1,790,073 | 913,399 | 3,024,435 |
| Duplications | 50—299 bp | 1,545,476^∗∗∗^ 644,083 | 2,505,797 | 754,287 | 391,311 | 1,138,254 |
|  | 0.3—4.9 kb | 1,495,159^∗∗∗^ 646,717 | 2,393,655 | 793,538 | 395,685 | 1,227,684 |
|  | 5—49 kb | 663,015^∗∗∗^ 258,878 | 1,235,982 | 354,688 | 186,860 | 537,661 |
|  | 50—249 kb | 5,279,236^∗∗∗^ 2,299,383 | 7,738,683 | 2,443,219 | 1,339,239 | 3,225,454 |
|  | 0.25—1 Mbp | 57,759,853^∗∗∗^22,418,461 | 92,387,936 | 51,499,104 | 18,058,220 | 95,936,696 |
| Translocations | NA | 94,117^∗∗∗^ 44,497 | 137,792 | 75,258 | 39,326 | 109,027 |

∗∗∗*P <* 0*.*001 for *H*_0_: *µ_Observed_* = *µ_Random_*; Mann–Whitney *U* test.

Table S15: Proportion of either hotspot or coldspot 10 kb genomic windows in the averaged neighboring windows located 10 kb upstream or downstream of the hotspot and coldspot genomic regions in the distal proximal and telomeric sub- regions of the barley chromosomes in the three analyzed populations HvDRR13, HvDRR27, and HvDRR28.

| Population | Window type | Distal subregion | Hotspot | Coldspot |
| --- | --- | --- | --- | --- |
| HvDRR13 | Coldspot | Proximal | 0.04 | 0.00 |
|  |  | Telomeric | 0.05 | 0.00 |
|  | Hotspot | Proximal | 0.00 | 0.49 |
|  |  | Telomeric | 0.00 | 0.34 |
| HvDRR27 | Coldspot | Proximal | 0.02 | 0.00 |
|  |  | Telomeric | 0.02 | 0.00 |
|  | Hotspot | Proximal | 0.00 | 0.37 |
|  |  | Telomeric | 0.00 | 0.33 |
| HvDRR28 | Coldspot | Proximal | 0.04 | 0.00 |
|  |  | Telomeric | 0.05 | 0.00 |
|  | Hotspot | Proximal | 0.00 | 0.58 |
|  |  | Telomeric | 0.00 | 0.45 |

Table S16. Comparison of motif abundance between CO hotspot and matched background windows. Mean counts of four previously reported crossover-associated DNA motifs within 10-kb CO hotspot windows and matched 10-kb background windows, shown separately for distal and pericentromeric genomic regions. CO hotspots were identified independently in each population (HvDRR13, HvDRR27, and HvDRR28), and 10-kb windows overlapping hotspots in at least two populations were selected as CO hotspot windows for subsequent analyses. Fold change represents the ratio of mean motif counts in hotspot windows to mean motif counts in background windows. Statistical significance was assessed using a two-sided Wilcoxon rank-sum test. Motif classes comprise PolyA/T, CTT/GAA, CT/GA, and CCN/GGN.

| **Region** | **Motif** | **Hotspot** **window** **mean** | **Background** **mean** | **Fold** **change** | **P** **value** |
| --- | --- | --- | --- | --- | --- |
| Distal | PolyA/T | 229.15 | 186.49 | 1.229 | < 0.001 |
|  | CTT/GAA | 362.7 | 395.15 | 0.918 | < 0.001 |
|  | CT/GA | 1210.51 | 1276.9 | 0.948 | < 0.001 |
|  | CCN/GGN | 1080.03 | 1076.45 | 1.003 | 0.68652 |
| Pericentromeric | PolyA/T | 202.47 | 169.87 | 1.192 | 0.00874 |
|  | CTT/GAA | 362.16 | 394.05 | 0.919 | 0.01052 |
|  | CT/GA | 1207.42 | 1285.93 | 0.939 | < 0.001 |
|  | CCN/GGN | 1176.26 | 1095.65 | 1.074 | 0.39897 |

2.5

1.5

0.5

0.15

0.05

1H

2.5

1.5

0.5

0.15

0.05

2H

2.5

1.5

Recombination rate (cM/Mbp)

0.5

0.15

0.05

SV span fraction in window (%)

3H

2.5

1.5

0.5

0.15

0.05

4H

2.5

1.5

0.5

0.15

0.05

5H

2.5

1.5

0.5

0.15

0.05

6H

2.5

1.5

0.5

0	200	400	600

Physical position (Mbp)

0.15

0.05

7H

Recombination rate	SV type:	Insertions	Deletions	Inversions	Duplications

Fig. S1: Distribution of recombination rate and structural variants (SV): insertions, deletions, inversions, and duplications, across the seven barley chromosomes. The displayed values repre- sent the spanned fraction of a 1 Mbp genomic window by the sum of all SVs of the same kind. The values were averaged across the 45 double round-robin (DRR) populations in 1 Mbp win- dows across the seven barley chromosomes. The vertical blue solid line indicates the position of the centromere in the Morex v3 reference genome and the vertical blue dashed lines indicate the pericentromeric region calculated across the 45 DRR populations.

raw data 10−500 kb

1H

0.5−3 Mbp

>3 Mbp

raw data 10−500 kb

2H

0.5−3 Mbp

>3 Mbp

raw data 10−500 kb

3H

0.5−3 Mbp

>3 Mbp

raw data 10−500 kb

4H

0.5−3 Mbp

>3 Mbp

raw data 10−500 kb

5H

0.5−3 Mbp

>3 Mbp

raw data 10−500 kb

6H

0.5−3 Mbp

>3 Mbp

raw data 10−500 kb

0.5 −3 Mbp

>3 Mbp

0

200

400

Physical position (Mbp)

600

7H

Fig. S2: Example of the graphical genotype for one recombinant inbred line (RIL). The four graphical genotypes for each of the seven barley chromosomes show, from the raw data, the different three crossover (CO) related parental marker block categories considered in this study: 10–500 kb, 0.5–3 Mbp, and >3 Mbp, respectively.

Recombination rate

Total SV load

Insertions

Deletions

1

0.8

**−0.82**

**−0.86**

**0.95**

**0.61**

**−0.69**

**−0.65**

**0.27**

**−0.24**

**−0.27**

**0.15**

**0.35**

**−0.4**

**−0.32**

**0.29**

**0.18**

**0.25**

**−0.3** **−0.33**

**0.26**

**0.13**

**0.21**

**−0.7**

**−0.68**

**0.53**

**0.34**

**0.56**

**0.28**

**0.63**

**0.65**

**−0.74−0.74**

**0.5**

**0.28** **0.27**

**0.56**

**0.53**

**−0.62**

**−0.55**

**0.46**

**0.27**

**0.28**

**0.77** **0.74** **0.74**

**0.48**

**0.81**

**−0.87−0.89**

**0.69**

**0.23**

**0.37**

**0.29**

**0.67** **0.65**

**0.56**

0.6

0.4

0.2

Recombination rate	**0**

Total SV load

**0.13**

**0.09**

**0.14**

**−0.03**

Insertions

Deletions

**−0.01**

1

0.8

**−0.16**

**−0.63**

**0.52**

**−0.05−0.03**

**0.1** **0.08**

**−0.01** **0.02**

**0.28**

**−0.18**

**0.16**

**0.11**

**−0.07**

**−0.01**

**0.01**

**0.08** **0.07**

**0.29**

**−0.03**

**0.06**

**−0.02**

**−0.18**

**−0.03**

**0.27**

**0.56**

**0.08**

**0.04** **0.04**

**−0.01−0.01**

**0.19**

**0.1** **0.04** **0.06**

**0.32**

**0.01**

**−0.2**

**0.23**

**0.82**

**0.55**

**0.72**

**0.16**

**−0.33**

**−0.29**

**0.06** **0.02**

**0.13**

0.6

0.4

0.2

Inversions

Duplications Parental sequence divergence

Gene density Methylation level: CpG

Methylation level: CHG

0

−0.2

−0.4

−0.6

−0.8

−1

Inversions

Duplications Parental sequence divergence

Gene density **−0.01**

Methylation level: CpG Methylation level: CHG

0

−0.2

−0.4

−0.6

−0.8

−1

[A] [B]

Fig. S3: Correlation matrix between recombination rate, the total structural variants (SV) load and such of the different types –insertions, deletions, inversions, and duplications– separately, the parental sequence divergence, gene density, and the methylation level at the sequence contexts CpG, CHG, and CHH, across 1 Mbp windows in the distal (A) and the pericentromeric (B) regions of the barley chromosomes for the average among the 45 double round-robin (DRR) populations. Pearson’s correlation coefficients are displayed and indicated with a color gradient from -1 (red) to 1 (blue).

1.00	1.00

● ●

●

●

●	●

●

●

●	●

●

●	●	●	●

●	●	●

●	●	●	●	●

●	●

●

●	●

●

●

●	●

●

●

●●	●

● ●●

●	●	●

●	●	●	●	●	●	●	●	●	●	●	●	●	●

●	●	●	●●	●	●	●	●	●

●	●	●

●

●

●	●	●	●	●●	●●	●●	●

●	● ●	●	●	●	●	● ●	●	●	●	●

●

●

●	●

●

●

●	● ●

●	●	●	●	●●	●

●	●

●	●	●	●	●	●

●	●

●	●	●

●	● ●	●	●	●	●	●	●

●	●

●

●	●	●	●

●	●	●

●	●	●	●	●

●	●	●	●	●	●	●	● ●

●	●	●	●	●	●	●	●	●	●	●

●

●

●

●	●

●	●	●	●	●

● ●	●	●	●	●	●	●	●	●	●

●	●	●	●	●	●	●	●

●	●	●	●	●	●	●	●	●	●	●

●	●	●	●	●	●

●

●	●	●

● ●●

●

●●	●	●	●	●	●	●

●	●	●	●	●	●●	●	●	●	●	●	●

●	●

●	●	●	●

●	●

●	●	●	●

● ●●	●

●	●	●

●

●	●

●	●	● ●	●	●	●	●	●	●	●	●	●

●●●	●

●	●

●

●	●

●	●	●	●	●	●	●	●	●	●	●	●	●

●

●	●	●	● ●

●	●	●	●	●	●

●	●

●

●

●

●

● ●

●	●	●

●	●	●	●	●	●	●	●

●

●

●	●	●	●	●	●	●	●

●	●	●	●

●

●●	● ●● ● ● ●●

●

●	● ●	●	●	● ●	●	●● ●	●	●	●

●

●	●	●	●	●	●	●

●	●	●

●	●	●

●	●

●	●	●	●	● ●	●

●	●	● ●	●	●	●	●

●	●	●	●	●	●	●	●	●

●

●	●	●	●	●	●	●	●	●	●	●	●

●	●	●	● ●	●	●	●	●	●

●	●	●	●	●	●	●	●

●

●	●	●	●

●

●	● ●	●	●

●	●	●

●

●	●	●	●	●	●

●	●	●	●	●	●	●	●	●	●

●	●	●

●	●	●	●	●	●	●

●	●	●	●	●	●

●	●

● ●

●	●	●	●	●

● ● ● ●● ●● ● ●●

●	●	●	●	●

● ● ● ● ● ● ● ● ●●

●	●	●	● ●	●	●	●	●

●	●	●	●	●	●● ●

●	●	●	●	●	●

●	● ●

●	●	●

●	●

●

●	●	●	●	●	●	●	●	●	●

●

●	●	●	●	●	●

●

●	● ●	● ●

●	● ●	●●	●	●	●	●	●

●	●	●

●	●	●

●	●	●	●

●	●

●	●	●

●	●	●	●	●	●	●	●	●	●	●	●	●

●	●

●	●	●

●

●	●

●

●

●	●	●	●

●

● ●	● ● ●	●	●	●	●

●	●	●●	●	●

● ●	●	●	●	●	●

●	●	●	●

●	●	●

●

●	●	●	●	●	●

●	●	●

●	●	●	●	●	●	●

●	●	●

●	●

●	●

●	●	●

●	●	●	●	●	●

● ●	●	●	●	●	● ●	●

●	● ●	● ●	●	●	●	●	●

●	●	●

●	●

●	●	●	●	●	●	●

●	● ●	●	●	●	●	●	●	●

●	●	●	●	●	●	●	●	●

●	●

●	●	●	●

●

●	●	●	● ● ●	●	●	●

● ● ● ● ● ●●

●	●	●	●	●	●	●	●	●	●●	●	●	●	●

●

●	●	●	●

●	●	●	●	●	●

●	●

●	●	●	●

●	●	●	●	●	●	●	●	●

●	●	●	●	●

●	●	●	●	●	●	●●	●

●	●	●	●	●	●

●●

●	● ●	●	●	●

●	●	●	●	● ●	●	●	●	●	●	● ●

●

●	●

●

●	●	●	●	●

●

●	●	●	●	●

●

●	●

●	●	●

●

●

●	●	●	●	●	●	●	●

●	●	●	●	●	●	●	●	●

●

●	●	● ●	●	●	●	●	●	●

● ●	●	●	●	●	●	●	●	●	●	●	●	●

●

●	●

●

●	●●	●	●	●	●	●	●	●	●	●	●	●	●	●

●

●●	●

●	●

●	●

●

●	●

●	●	●	●	●	●	●	●

●

●	●	●	●	● ●	●	●	●	●	●	●	●	●	●	●

●	●	●

● ●	●	●	●	●	● ●	●	●

●	●	●	● ●● ●

●	●	●●	●	●	●	●	●	●	●

●

●●

●	●	●	●	●	●	●	●● ●	●

●	●	●	●	●	●	●	●	●	●	●	● ●	●	●	●	●

●	●

●

●	●

●	●	●	●

●

●	●	●

●	●	●	●●	●

● ●	● ● ●	●	●	● ●	●	● ●	●	●

●	●

● ●

●	●

●

●	●	● ●	●	●	●	●	●	●	●	●	●	● ●	● ●	●	●	●	●	●

●	●	●

●

●	●	●

●

●	●	●

●	●	●	●	●

● ● ●	●	●

●	●	●	●	●	●	●	● ● ●	●● ●

●	●

●	●

●	●	● ●	●	●

●	●	●	●

●	● ●	●	●	●	●	●	●	●	●	●	●

●	●	●

●

●	●	● ● ●	●	●	●	●

●

●

●

●	●

●

●	●	●	●	●	●	●	●	●	●	●	●	●	● ●	●	●	●	●	●	●	●	●

●	●	●	●

●

●	●	●	●	●	●

●	●	●

●	● ●	●	●	●

●	●	●	●	●	●	● ●	●●	●

●	●	●	●	●	●	● ●	●

●

●

●	●	●	●	●	●	●	●	●	● ●	●

●	●

●

●

●

●	●	●

●	●

● ● ● ● ● ● ● ●●

●	●	●

●	●	●	●	●	●	●	● ●● ●

●	●	●

●	●	●	●	●	●	●	●	●	●	●	●	●	●

●	●	●	●	●	●	●

●	●

●	●	●	●

●	●	●	●	●	●	●	●	●	●

●	● ●	●	●	●

●	●	●	●	●	●	●	●	●	●

●	●	●	●	● ●	●	●	●	● ●	●	●

●	●	●

●	●	●

●	●

●	●

●	●	●	●	●	●	●	●	●	● ●

●	●	●	●

●	●	●	●

●	●	●	●	●	●	● ● ●	●	●	●	●● ●	●	●	●

●

●	●	●	● ●	●●	●●	●	●	●	●	●	●	●	●

●	●	●

●	●	●	●	●	●	●

●

●●	●	●	● ●

● ●	●	●

●

●

●	●

●	●	●	●

●	●	●

●

●	●

●	●	●

●	●	●	●	●

●

●	●	●	●	●	●	●	●	●	●

●

●

●	●●	●

●	●	●	●	●	●	●

●

●	●	●

●	●	●

●	●	●

●

● ● ● ●● ● ●●

●	●

●	●

● ● ● ● ● ●●

● ●	●	●	●	●

●	●	●	●

● ●● ● ● ●●	●

●	●	●	●	●	●

●	●	●	●	●

●	●	●	●	●

●	●

●	●

●	●

●

●

●●

●

●	●	●

●	● ●	●	●

●

●	●	●	●	●	●	●	●● ●

●	● ●	●

●	●	● ●

● ● ● ● ●● ● ● ● ● ● ●●

●	●

●	●

●

● ●	●	●	●	●

●	●	●	●	●	●

●	●	●	●	●	●	●	●●	●

●	●	●	●

●	●	●

●	●	●●	●

●	●● ●	●	●	●●	●

●	●	●	●	●	●	●

●	●	●	●	●	●	●

●	●●	●	●

●

●	●	●	●	●	●	●	●	●	●	●	●	● ●	●	●	●

●	●

●

●	●	●	●	●

●	●	●

●	●	●	●	●	●	●	●

●	●	●	●	●	●	●

● ●	●

●	●	● ● ●	● ●	●	●	●	●	●	●	●	●	●	●

●

●	●	●	●

●

●	●	●	●	●	●	●

●	●	●	●

●

●

●	●

●	●

●

●

●

●

●	●	● ●	●	●	●	●	●	●

● ●●

●

●	●	●	●	● ●	●	●	●	●	●

●	● ●

●	●	●	●	●	●	●	●	●

●

●

●

● ●

●●	● ●	●	●	●

●

●

●	●	●	●

●	●	●

●

●	●	●

●	●	●	●	●	●

●

●	●	●	●	●

●	●	●	●	●	●	●	●	●	●	●

● ● ● ● ● ● ● ● ● ● ● ● ● ●● ● ●●

●

●	●	●	●

●	●	● ●	●	●	● ●

●	●	●	●	●	●	●	●

●	●

●	●	●	●	●

●	●	●	●	●	●	●	●	●	●	●	●	●

●

● ● ●● ●●

●	●	●

●	●	●	●	● ●	●	●	●	●	●	●

●	●	●

●	●	●	●

●

●	●	●	●	●	●	●	●	●	●

●	●	●	●

●	●	●	●	●

●

●	●	●	●

●

●	●	●	●	●	●

● ●	●

●

●	● ●	●	●●	●	●

●●	●	●	●	●

● ●●	●	●	●	●	●	●

●	●	●	●

●	●	●	●	●	● ●	●	●	●	●

●

●	●	●	●	●	●	●

●	●

●

●	●	●

● ●	●	●	●	●

●

●	●	●	● ●	●	● ●	●	●

●	●	●	●	●	●

●	●	●	●	●	●	●	●	●	●	●	●	● ●

●	●

●	● ●

●

●	●

●	●

●

●	●	●	●	●	●	●	●	●	●	●	●	●	●	●	●	●

●●	●

●	●	●	●	●	●	●

●	●	●●	●	●

●	●	●	●	●

●	●	●	●	●	●	●	● ●	●	●	●

●	●	●	●●	●	●	●

●	●	●	●

●

●

●	●

●	●	●	●

●	●

● ● ● ● ● ● ● ● ● ● ● ● ●●

●	●

●	●	●	●

●	●	●	●

●

●	●

●	●	●	●	●	●

●	●	●

●	●	●	● ●

●	●	● ●	●	●

●

●	●	●	●	●	●	●	●	●

●

●	●	●	●	●

●	●	●

●	●	●

●	●	●

●

●	●	●	●	●	●	●	●	●	●	●

●

●	●	●●	●

●●●	●	●	●	●

●

●	●	●	●	●	●

●	●	●	●	● ●	● ●	● ●

●	●	●	●	●	●	●	●	● ●	●

●	●	●	●●	●	●	● ●	●

●

●

●	●	●	●	●	●	●	●	●	●	●

●	●	●	●	●	●

●	●

●

●	●	●	●	●

●	●

●

●

●	●

●	●	●	●	●	●	●	●	●

●	●	●	●	●	●	●	●

●

●	●	●	●	●	●	●	●	●	●	●

●

●	●	●

●	●	●	●	●	●

●	●	●	●●	●

●	●

●	●	●	●	●	● ●	●	●	●

●

●	●	●

●	●

●	●	●	●	●

●

●	●

●

●	●	●	●	●	●	●	●	●	● ●	●●	●	●	● ●	●	●	●	●	●	●	●	●	●

●	●

●

●

●	●	●	●	●	●	●

●	●	●	●	●

●

●	●	●	●	●	●	●

●	●	●

●	●	● ●	●	● ●

●	●	●	●	● ●	●	●

●	●	●	●	● ●	●	●	●

●	●	●	●	●	●	●	●	●	●	● ●

● ●	●	●

●

●

●	●	●

●	●	●	●	●	●	●	● ● ●	●	● ●

●

●

●

●

●	●

●	●	●	●	●	● ●	●	●●	●	●	●	●	●	●●	●	●	●	●	●	●	●	● ●	●

●	●	●●	●	●

●	●	●	●	● ●	●

●

●	●	●	●	● ●	●	●	●	●	●

● ●	●

●

●

●	●	●	●	●	●	●	●	●	●	●	●	●	●

●	●	●	●	●

●	●	●	●	●	●	●	●	●

●	●	●	●	●	●	●	●

●	●	●

● ● ● ●●

●	●

●	●	●	●	●	●	●●	● ●

●	●	●	●	●	●	●	●	●	●	●	●	●	●	●	●	●	●

●	●

●	●	●

●

●

●	●	●	●	●	●

●	●●	●	●	●	●	●

●	●

●	●

●

●

●	●

●	●

●	● ●	●	●

● ● ●● ● ● ● ● ● ● ● ● ● ● ●●

●	●

●	●	●	●	●	●	●	●	● ●

●	●	●	● ●	●

●

●	●	●	●	●	●

●	●	●

●	●	● ●	●	●	●	●

●

●	●	●	● ●	●	●

●	●

●	●	●

●	●	●	●	●	●	●	●	●	●	●	●	●	● ●	●	●

●	●

●	●

●	●	●

●	●	●	●	●

● ●	●	●	●	●	●	●

●	●	●	●

●	●

●	● ● ●

●	●

●	●	●	●	●	●	●	●●	● ●

●

●	●

●	●	●

●	●	●	●	●	●	●	●	●	●	●●	●	●

●	●	●	●	●	●	●	●

●

●	●

●	●

●	●	●	●

●	●

●	●

●	●	●	● ●	●	●

●

●	●	●	●	●

●	●	●

●	●	●	●	●	●	●	●	● ●

●	●	●	●	●	●	●	●	●	●	●	●	●	●	●	● ●

● ●	●	●	●	●	●	●	●	●	●	●	●

●	●

●

●	●	●	●	●	● ●	● ●	●	●

●	●

●	● ●	●	●	●	●	● ●	●	●	● ●	●	●	●

●	●	●

●	●

●	●	●	●	●	●	●	●	●	●	●	●	●	●	●	●	●	●	●	●	●	●	●	●	●	●

●	●

●	●	●

●

●	●	●	●	●

●	●	●	●	●

●	●	●	●	●	●●	●

●	●	●	●

●	●	●	● ● ●

●	●	●	●	●	●	●●	●	●	●

●	●	●

●

●	●	●	●	●	●	●	●	●	● ●	●	●	●	●	●

●	●

●

●

●●	●	●

●	●

●●	●	●●	●● ●

●	●

●

●	●

●	●	●	●	●	●	●	●

●	●	●	●	●	●	●

● ●	●	●	●	●	● ●	●	●	●	●	●	●	●	●	●	●

●	●	●	●

●	●

●	●	●	●	●

●

●

●	●	●	● ●

●	●

●	●	●	●	●	● ●	●	●	●	●	●	●	●	●

●	●	●	●	●

●	●	●

●	●	●	●	●	●	●	●

●	●	●	●

●	● ●	●	●	●	●	●	●

●	●	●	●

●	●	●	●	●	●	●

●	●	●	●	●	●	●	●	●	●

●	●	●	●

●

●	●	●	● ●●●	●

●	●	●

●	●

●	●	●	●	●

●

●	●	●	●

●

●	●	●	●	●	●	●

●

●	●	●	●	●	●	●	●	●	●	●	●	●

●	●

●● ●	●	●	●	●	●	●	●	●	●

●	●	●	●	●	●	●	● ●	●	●

●

●●

●	●

●

●	●

●	●	●

●	●	●	●	● ●●	●	●	●

●

●	●	●	●●	●	●	●

●

●	●	●	●	●	●	●	●	●●	●

●	●	●	●	●	●	●	●	●

●	●

●

●●	●	●

●●	●

●

●

●	●	●

●	●	●

●

●	●	●	●	●●	●	●	●

●	●

●	●	●	●

●●	●●	●	●	●

●	●

●	●

●	●	●	●	●	●	●	●	●	●	●	●	●

●	●	●	●	●	●	●	●

●	●	●	●	●	●

●	●

●	●

●	●	●	●

●

● ●	●

●	●	●●	●	●

●	●	●	●

●	●	●

●	●	●	●	●	●	●	●

●	●	●	●

●

●	●	●	●	●	●	●	●	●	●	●	●	●	●	●	●	●	●	●	●

●

●	●	●

●

●	●	●

●

●	●

●

●	●	●	●	●	●

●	●	●●	●	●	●

●	●	●	●	●	●	●	●

●	●●	●	● ●	●	●	●	●	●	●

●

●

●●	●● ●	●	●	●	●	●	●●	●	●	●	● ●	●	●

●	●

●	●	●	●	●

●	●	●	●

●	●

●

●

●	●	●

●	●	●	●	●

●	●

●	●	●	●

●	●	●	●	●	●	●	●	●	●

●	●	● ●	●	●	●	●

●

●

●	●	●	●	●	●	●	●	●	●	●	● ●	●	●

● ● ●●

●	●

●	●	●	●	●●	●	●	●	●

●	●	●	●	●

●	●	●	● ●	●	●	● ●	● ●

●	●	●	●	●	●	●	●	●	●	●	●	●	●

●

●	●

●	●

●	●	●	●	●	●	●	●	●	●	●	●●	●	●

●	●	●	●	●	●	●	●	●	●●	●	●	●

●

●	●	●	●

●	●

●

●

●	●	●

●	●●	●	●	●	●

●	●	●	●	●	●	●	●	●	●	●	● ●

●	●	●	●	●	●	●	●	●	●

●

●	●	●	●

●

●	●●	●	●

●	●	●	●	●	●	●	●	●

●	● ●	●	●	●

●	●

●	●	●	●	●	●	●	●

●

●	●

●	●	●

●	●	●	●	●	●	●

●	●	●	●

●

●	●

●	●

●	●

●	●	●	●	●	●

●

●

●	●	●	●

●	●	●

●	●

●	●	●

●

●	●	●	●	● ●	●

●	● ●	●	●	●	●	●	●

●	●	●

●	●	●

●

● ● ● ●●

●

●	●	●	●

●	●	●

●	●●	●	●	●	●	●

●	●	●

●

●●	●

● ●	●	●	● ●

●	●

●	●	●	●	●

●	●

●	●	●	●	●	●	●	●	●

●	●	●	●	●	●

●	●●●	●	● ●	●	●●	●	●	●	● ●	●	●	● ●

●	●	●	●	●	●	●	●

●	●	●

●	●

●	●	●

●

●

●	●	●	● ●	●	●

● ● ●●

●	●	●	●	●	●●	●	●	●	● ● ●

●	●	●	●	●	● ●	●	●	●	●	●

●

●

●	●	●

●

● ●

●	●	●

●

●	●	●	●	●	●	●	●

●	●	●	●	●	● ●

●	●	●	●	●●	●	●	●	●	●	●	●	●

●	●	●

●	●

●

●	●	●	●	●	●	●	●

●	● ●	●	●	●	●

●	●	●

●

●	●

●	●	●	●

●	●	●	●	●	●	● ●	●	● ●	●	●	●●	●	●	●	●	●●	●

●	●	●	●	●	●	●

●	●	●	●

●

●	●	●

●

●

●	●

●

●	●

●

●	●

●

●	●

●	●	●	●	●	●	●	●	●	●	●	●

●	●

●	●	●	●	●	●	●	●	●	●	●	●	●	●	●	●	●	●

●	●	●	●

●	●

●	●	●	●

●●

●	●	●	●

●	●	●	●	●	●	●

●

●	●

●

●	●

●

●	●	●	●

●	●	●	●	● ●	●	● ●	●

●

●	●	●	●	●	●	●

●	●	●	●	●

●

●

●●	●	●

●

●	●

●	●	●

●	●	●	●	●

●	● ●

●	●	●	●	● ●	●	●	●	●

●	●	●	●	●	●	●	●	●	●	●	●	●	●	●	●

●	●	●

●	● ●	●	●	●	●	●

●	●	●

●	●

●

●

●	●

●	●	●

●	●	●	●	● ●	●	●	●

●

●

●	●	●	●	●	●

●	●	●

●	●	●	●	●	●	●	● ●

●	●

●	●	●

●

●	●	●	●

●	●	●	●	●

●	●

●	●	●	●	●	●

●	●	●●	●

●

●	●	●	●	●	●	●●	●	● ●

●	●	●	●	●	●●	●	●	●

●	●	●	●	●	●

●	●	●	●	●

●	●	●	●	●	●

●	●	●	●	●	●	●	●

●	●	●	●	●	●	●●	●	●	●	●	●

●

●	●

●	●	●	●

●

●	●	●	●	●	●

●	●

●

●

● ● ● ●●

●	●

●	●

●	●	●	●

●	●	●	●	●

●

●

●	●	●

●

●	●

●	●

●	● ●

●

●	●

●	●

●	●	●	●	●	●	●	●	●

●	●	●	●	●	●	●	● ●	●	●	●	●	●	●	●

●

●	●	●

●

●	●	●	●

●	●	●	●

●	●	●

●

●●	●●	●	● ●	●	●	●	●	●	●

●	●	●

● ● ● ●	● ● ● ● ● ● ● ●●

●	●●	●	●	●	●

●	●	●

●	●

●●	●

●	●	●

●

● ●

●	●

●	●	●	●	●	●

●	●	●	●	●	●

●

●	●	●	●	●

●

●	●

●

●

●

●

●

●	●

●	●	●

●	●	●	●	●	●	●	●

●	●	●	●	●	●	●	●	● ● ●

●	●

●	●	●	● ●	●

●

●●	● ●

●

●●

●

●	●	●	●

●	●

●	●

●	●

●	●	●

●

●	●

●	●

●

●	●	●	●

●

●	●	●	●	●	●	●	●	●

●

●	●

●	●	●

●	●	●	●

●	●	●	●	●	●	●	●

●	●	●	●	●	●	●

●	●	●	●	●	●●	●

● ● ● ●●

● ● ●● ●	● ● ● ● ● ● ● ●●

●	●	●	●

●

●

●

●	●

●	●

●

●	●

●	●	●	●	●	●	●	●	●	●	●	●	●

●	●	●	●

●

●	●	●

●

●

●

●	●	●	●	●	●	●

●	●	●	●	●	●	●	●	●

● ●	● ●	●	●	● ●	●	●	●	●

●	●

●

●	●

●	●	●	●

●●	●●	●	●	●

●	●	●

●●	●	●

●

●	●	●	●	●	●●	●	●	●	●	●

●	●	●	●	●

●	●	●	●	●	●	●

●	●	●

●	●	●	●	●

●

●	●	●	●

●	●	●

●

●

●	●	●	●

●	●	●

●	● ●

● ●	●	● ●

●	●

●	●

●

●	●

●	●

●	●

●

●

●

●

● ●

●	●	●

●	●	●	●	●

●	●	●	●	●	●	● ●	●●	●	●

●	●	●	●	●	●	●	●	●	● ●	●	●	●	●	●	●	●	●	●	●	●	●

●

●

●

●	●

●

● ●

●	●

●

●	●	● ●	●	●

●

● ●	●	●	●	●	●	●	●	●

●	●	●	●	●	●	●	●	●

●

●	●	●	●

●

●	●

●	●

●

●

●

●	●	●

●

●	●

●	●

● ● ● ● ● ● ● ● ● ● ● ● ●●

●	● ●	●	●	●	●

●	● ●	●	●	●

●	● ●	●

●

●	●	●	●	●	●	● ●	●

●	●

●

●

●	●

●

●

● ●	●

●

●	●

●

●

●	●	●	●

●	●	●	●	●	●

●	●	●	●	●	●	●	●	●	●

●	●

●

●	●	●	●	●	● ●	●	●	●	●	●	●

●	●

●	●	●	●	●

●

●	●	●

●

●

●	●	●

●

●

●

●

●	●	●	● ●	●

●

●

●

●

●

●	●	●

●

●

●	●	●	●	●	●

●	●	●	●

●

●

●

●

●

●	●	●	●

●	●●	●	●	●	●	●

●	●	●	●	● ●	●	●	● ●

●	●	●

●	●	●	●

●	●	●	●	●	●	●	●	●	●	●	●	●	●	●

●

●	●

●

●	●	●

●	●

●	●

●

●

●	●

●

●	●

●

●	●	●	●	●	●	●	●

●	● ●	●	●	●	●	●	●

●	●

●	●	●	●	●	●

●	●	●	●	●	●	●

●

●	●

●	●	●

●	●	●

●

●	●	●	●

●

●

●	●	●	●

●

●

●

●

●	●

●

●

●

●

●

●	●	●●	●	●

●	●	●	● ●	●	●	● ●	●

● ● ● ● ● ● ● ● ● ● ● ● ● ●●

●

●	●

●

●	●	●

●	●

●	●	●	●	●	●	●	●	●

●	● ●	●	●	●

●	●

●	●

●	●	● ●

●	●	●	●	●	●	●	●	● ● ●	●	●

●

●	●

●	●	●

●

●

●

●	●	●

●

●

●

●	●

●

●

●	●	●	●

●	●

●	●

●

●	●	●	●

●	●	●	●	●

●

●

●

●	●

●	●	●	●	●	●	●

●	●	●	●	●	●

●	●	●	●	●●

●

● ●	●

●

●	●	●

●	● ●	●	●	●

●

●

●

● ● ● ●●

●	●	●	●

●	●	●

●	●	●

● ● ● ● ● ● ●●

●	●	●	●

● ●●	●

●

●	●	●	●●	●

●	●	●	● ●	●	●	●

●	●	●	●	● ●	●	●

●	●

●

●

●

●

●	●	●	●

●

●

●

●	●

●

●	●

●

●	●

●	●	●	●	●	●	●	●	●

●

●

●

●

●	●●	●	●

●

●

●	●

●

●	●	●	●	●

● ●	●	●	●	● ●	●

●	●	●	●	●

●	●	●	●	●	●	●	●	●

●	●	●	●	●

●

●

●	●	●	●	●	●	●	● ●	●	●

●	●	●	●

●	●	●

●

●

●	●

●

●

●	●	●

●	● ●	●

●	●

●	●

●	●

●

●

●	●	●	●

●

●	●	●

●	●	●	●	● ●	●

●	●

●

●	●	●	●	●	●

●

●●	●	●	●	●

●	●	●	●	●	●	●

●

●

●

●

●

●

●	●

●	●

●	●

●	●	●	●	● ●	●	●

●

●

●

●

●

●

●	●	●	●	●

●

●

●

●

●	●	●

●

●

●

●	●	●

●	●

●	●	●

●

●	●	●

●

● ●	●	●	●	●	●	●	●

●	●	●

●	●	●	●	●	●

●

●

●

●

●

●	●	●

●	●	●	●	●	●	●	●	●	●	●

●

●

●

●

●

●

●

●	●	●

● ●	●	●	●

●	●	●	●●	●	●	●●	●

●

●

●

●	●	●

●	●	●

●

●	●	●	●	●	●●	●	●

●

●	●	●

●	●	● ●

●	●	●	●

●	●	●

● ●	●	● ●	●	●	●	●	●	●

●

●	●

●

●	●	●	●	●	●

●	●	●	●

●

●

●

●

●	●

●	●	●	●

●

●

●

●	●	●	●

●	●	●	●	●

●

●	●	●	●	●

●

●	●

●	●	●	●	●

●

●	●

●	●

●	●

●	●

●	●	● ●	●

●

●	●	●	●

●

●

●	●	●	●	●

●	●

●

●

●

●

●	●

●	●	●	●	●	●	●	●	●	●

●	●

●	●

●	●	●	●

●

●

●

●	●	●

●	●	●	●	●	●

●

●

●

●

●

●	●	●	●	●	●	●	●

●	●	●

●	●	●	●	●

●	●	●	●	●

●

●	●	●	●

●

●

●	●	●

● ●

●

● ●●

●	●

●	●	●	●

●	●

●

●	●	●	●	●

●	●

●	●	●	●	● ●

●

●

●

●

●

●

●	●

●

●	●

●

●	●	●	●	●	● ●	●	●	●	●

●	●	●	●	●	●	●	●	●	●	●	●	● ●● ●	●

●	●	●	●

●

●	●

●	●

●

●

●

●	●	●	●	●	●	●	●

●	●	●	●

●

●	●	●	● ●

●	●	● ●

●

●	●	●	●

●

●	●	●

●

●	●

●	●	●

●

●

●	●	●	●	●

●	●

●

●	●	●	●

●

●	●	● ●

●	●	●●	●

●	●	●	●

●	●

●

●

●

●

●

●

●

●	● ●

●	●

●

●	●

●

●

●	●	●

●	●	●

●	●	●

●	●

●

●

●

●	●

●	●	●	●	●	●

●	●

●

●	●	●	●	●	●	●

●

●●	●	● ●	●	●

●	●

●	●

●

●	●

●	●

●	●

●

●

●

●	●	●

●	● ●	●	●

●

●	●	●	●

●	●

●	●

●

●	●●	●

●	●

●

●

●

●	●

●

●	●

●	● ●	●	●	●

●	●

●

●	●	●

●

●

●

●

●	●

●	●

●

●	●

●

●

●	●

●	●	●	●	●	●	●	●	●

●	●	●	●

●

●

●

●	●

●

●	●

●	●	●	●	●

●	●	●	●	●

●

●

●	●

●	●	●	●	●	●	●	●	● ●

●	●	●	●	●

●

●	●	●	●

●	●	●	●	●	●

●	●

●	●	●	●

●

●	●	●

●

●

●

●

●

●

●

●

●

● ●	●

●	●	●	●	●

●

●	●	●

●	●	●●	●	●	● ●

●

●

●

●	●

●	●

●

●

●

●	●

●	●

●	●	●	●

●

●	●

●	●	●	●

●

●

●	●	●	● ●	●	●

●

●	●	●	●

●	●	●

●

●

●	●

●

●	●	●	●	●	●

●	●	●	●	●	●

●

●

●	●

●

●

●	●	●	●

●	●●

●	●	●

●

●	●

●	●	●

●	●

●	●

●

●

●

●

●	●

●	●	●	●	●

●

●

●

●

●

●	●	●	●

●	●

●

●

●

●

●

●	●	●

●	●

●	●	●	●	●	●

●	●	●	●	●	●	●

●	●	●

●	● ●●	●	●● ●	●	●	● ●	●	● ● ●	● ●	●	●	●●	●	●	●	●	●●	●	●

●	●	●	●	●	●

●	●	●	●	●	●

●	●

●

●●	●	●●	●	●	●	●

●	●	●

●	●

●	●	●

●

●	●	●	●	●	●	●	●	●	●	●	●	●	●	●	●	●	● ●	●	●

●	●

●	●	●	●

●	●	●	●	●	●	●	●	●	●	●

●

●	●●	●	● ●	●	●	●	●

●	●	●	●	●	●

●

●

●●

●●	●

●	●	●	●	●	●	●	●	●	●

●	●	●

●	●	●	●	●	●	●	●	●	●	●	●	●	●	●	●	●

●	●

●	●	●	●	●

●	●

●	●	●	●

●	●	●	●	●	●	●	●	●	●

●	●	●	●	●	●	●	●	●	●

●	●	● ●	●	●	●	●

●	●	●

●	●	●	●	●	●	●	● ●	●	●	●	●	●	●	●	●	●

●	●	●

●

●	●

●

●	●

●	●

●

●	●	●

●	●

●

●

●	●	●	●	●	●

●	● ●	●	●	●	●	●

●	●	●	●	●

●	●

●	●	●

●

●

●	●●	●	●	●

●	●	●	●	●

●	●

●	● ●	●	●

●	●●	●	●

●	●	●	●	●	●	●●	● ●	●

●	●	●	●	●	●	●

●●	●	●	●	●	●

●	●	●	●	●	●	●●	●	●	●	●	●	●

●

●

●

●

●

●

●	●	●	●	●	●	●	●	●	●	●

●	●	●	●●	●	●

●	●

●	●	●	●

●	●	●

●	●

●	●	●	●	●

●	●	●	●	●

●	● ●	●	●

●	●	●

●	●

●	●	●	●	●	●	●

●

●

●	●	●	●

●	●	●	●	●

●	●	●	●	●	●	●

●

●	●

●	●	●	● ● ●	●	●	●

●

●

●	●	●	●

●

●	●	●	●	●	●	●	●	●	●	●	●	●	●	●	● ●	●	●	●	●	●	●	●	●	●	●	●

●	●	●	●

●

●	●	●	●

●	●	●	●	●	●	●●	●	●

●	●

●

●

●	●	●	●	●	●	●	●

●	●

●

●

●	●

●	●	●

●	●

●

●

●	●	●	●	●	●

●	●	●	●	●	●	●	●	●	●	●	●

●	●

●	●

●

●

●

● ●	● ●	●

●

●

●

●

● ●	●	●

●

●	●

●

●	●● ●	●	●	●	●	●

●

●	●	●	●

●

●

●

●

●

●

●	●	●	●

●	●

●	●	●	●	●	●	●

●

●

●●

●

● ●	●	●	●●	●	●	●

●

●

●

●	●

●

●

●

●

●	●	●	● ●	●

●	● ●	●

●	●	●

●

●	●	●	●	●

●	●	●

●

●

●	●

●	●

●	●

●

●

●

●

●

●

●

●	●	●	●

●

●

●	●	●	●	●

●

●

●

●

●	●

●	●

●

●	●

●●	●	●

●

●

●	●	●

●	●

●

●	●

●	●	●	●	●

●

●

●	●	●	●	●	●	●

●●

●

●

●

●

●

●	●

●

●	●	●	●

●	●	●

●

●	●	●

●

●	●

●

●	●

●

●

●	●

●

●	●

●

●

●

●

●

●	●

●

●	●	●

●

●

●	●

●

●	●	●	●

●

●	●	●	●

●	●

●

●

●

●

●

●	●

●

●

●

●	●

●	●

●	●	●

●

●	●

●

●

●

●	●	●

●	●	●

●●

●	●

●	●	●	●	●	●

●	●

●	●

●

●

●	●	●	●

●	●	●	●

●	●

●

●	●

●

●	●

●	●

●

●

●	●

●

●

●	●	●	●

●	●	●

●

●

●	●	●

●	●

●

●

●

●

●

●	●	●

●

●	●	●	●

●	●

●

●	●

●	●

●

●

●

●	●	●

●

●

●	●

●	●

●

●	●	●	●	●	●	●

●

●

●

●

●	●

●

●

●

●

●

●	●

●	●

●

●

●

●	●

●

●

●

●

●

●

●	●

●	●

●	●

●

●

●	●

●	●

●	●	●	●

●

●	●	●	●	●	●

●

●

●	●	●	●

●	●	●

●	●	●	●	●

●

●

●

●

●

●	●	●

●

●

●

●

●	●

●

●

●

● ●	●

●	●	●	●	●

●	●

●

●	●

●

●

●

●

●

●

●

●	●	●	●

●	●

●

●

●

●

●

● ●

●	●	●

●

●

● ●

●

●●

●

●

●	●	●

●

●

●

●	●

●

●

●

●

●

● ●

●

●	●	●

●

●

●

●

●

●

●

●

●

●

●

●

●

●

●

●	●

●

●

●

●

●

●

●

●	●

●

●

●

●

●

●	●

●

●

●

●	●	●

●

●

●	●

●

●	●

●	●

●

●

●

●	●	●	●	●

●

●

●

●

●	●

●

●	●	●

●	●

●

●

●

●	●

●

●

●

●

●

●

●

●	●

●

●

●

●

●

●

●

●	●

●

●

●

●

●	●	●

●	●

●

●

●

●

●

●

●	●

●	●

●

●	●

●

●

●	●

●

●

●

●

●

●

●	●

●

●

●

●

●

●

●

●

●

●

●	●	●	●	●	●	●	●	●	●

●

●

●

●

●

●

●

●	●

●

●	●	●	●

●	●

●	●

●

●

●	●

●	●

●

●

●	●	●

●	●

●

●

●

●

●

●	●

●

●

●	●	●

●	●	●	●

●

●	●

●

●

●	●	●	●

●	●

●	●

●	●	●	●

●

●

●

●

●

●

●

●

●

●

●

●

●

●	●

●	●

●

●

●

●

●

●	●

●

●

●

●

●

●

●

●

●	●	●

●

●

●

●

●

●

●

●

●	●

●

●

●

●

●	●

●

●	●

●	●

●	●

●

●	●

●	●

●

●	●	●

●

●

●	●

●	●

●

●

●	●	●

●	●

●

●

●

●

●

●	●

●	●

●

●

●

●	●

●	●

●	●

●

●	●

●

●	●

●	●

●

●

●

●	●	●

●

●

●	●

●	●

●

●	●	●

●	●	●

●	●

●	●

● ●

●

●

●

●

●

● ●

●

●	●	●

●

●

●

●

●	●

●	●

●

●

●

●

●

●

●

●

●

●

●	●

●

●

●

●	●

●

●

●	●

●

●

●	●

●	●

●

●

●	●

●

●

●

●	●

●

●

●

●

●

●

●

●

●

●

●

●	●

●

●

●

●

●

●

●

●

●

●

●

●

●

●

●	●

●	●	●	●

●

●

●

●	●

●

●●

●

●

●

●	●	●	●	●

●

●	●

●

●	●

●

●	●

●

●

●

●	●

●

●

●	●	●

●

●	●	●

●

●

●	●

●

●	●

●	●

●	●

●	●

●

●

●

●

●

●

●

●

●

●

●

●

●

●

●

●

●	●

●

●

●

●

●

●

●

●

●

●

●

●

●

●

●	●

●

●

●

●

●

●

●

●	●

●	●

●

●

●

●

●

●

●

●

●

●	●

●

●

●

●

●

●

●

●

●

●

●

●

●

●

●

●

●	●

●

●

●

●

●

●

●

●

●

●	●

●	●

●	●

●

●●

●

●	●

●

●

●

●

●

●

●

●

0.75	0.75

0.50	0.50

Relative value

Relative value

0.25	0.25

0.00

0.00	0.25	0.50	0.75	1.00

Relative sequence parental divergence

0.00

0.00	0.25	0.50	0.75	1.00

Relative total SVs load

1.00	1.00

●	●	●	●	●	●

●	●

●

●	●

●

●

●	●	●	●	● ●	● ●	●	●	●

●	●	●	●	●	●	●	●	●	●

●

●	●	●

●

●

●	●

●	●	●	●	●	●	●	●	●	● ●	●	●	●	●	●

●	●	●

●

●	●	●	●

●	●

●	●

●	●

●	●	●	●	●	●	●	●

●	●	●	●	●	●	●	●	●	●	●●	●	●

●	●	●	●	●	●	●

● ●	●	●	●	●	●	●	●	●	●

●	●	●

●	●	●

●

●	●	● ●	●	●	●	●	●	●	● ●	●●	●	●	●	●	●

●

●	●	● ●	●	●	●	●	●	●	●	●	● ●	●	●	●	●	●	●	●

●	●	●	●	●	●

●●	●	●	●	●	●	●	●	●	●	●	● ● ● ●	● ●●	●	●	●	●	●	● ●	●	●	●	●	●	●	●

●

●	●

●

●	●	●	●	●	●	●

●	●	● ●	●	●	●	●	●	●	●	●

●	●●	●	●	●	●	●

●	●	●	●	●	●	●	●	●

●	●	●	●

●

●	●	●	●	●	●	●	●	●	●	●	●	●	●	●	●

● ●

●	● ●

●	●	● ●	●	●	●	●	●	●	●	●

●	●	●	●	●

●	● ●	●	●	●	●	●	●	● ●●	●	●	●

●	●

●	●

●

●	●	●	●	●	●	●

●	●	●	●	● ●	●●	●

●	●	●	● ●	●	●	●

●

●	●	●	●	●	●	●	●	●	●	●	●	●	●	●

●	●	●●	●	●	●	●●	●	●	● ●	●	●	●	●	●	●	●	●	●	●	●	●	●	●	●	●	●	●	●	●●	●	●	●

●	●	●	●	●	●

●	●

●

●●	●	● ●	●	●	●

●	●	●	●	●	●	●	●	●	●	●	●	●	●	●	●	●	●	●	●	●	●	●	●	●	●	●	●	●

●	●

● ● ● ● ● ● ● ● ● ● ● ● ● ● ● ● ● ● ● ●●

●	●	● ●	●	●	●

●	●	●

●

●	●	●	● ●	●	●	●	●	●	●	●

●	●	●

●	●	●	●	●	●	●	●	●	●	●	●

●	●	●	●	●	●	●	●	●	●	●	●	●

●	●	●	●	●

●	●	●	●	●	● ●

●	●	●	●	●	●	●	●	●	● ●	●	●	● ●	●	●	●	●	●	● ●	● ●	●	●	●	●	●	●

●

●	●	●

●	●	●	●	●

●	●	●	●●	●	●	●

●

●	●

●

●	●

●	●	●	●	●	●	●	● ●	●	● ●	●	●	●	●●	●	●	● ●	●	●	●	●	●	●	●	●

●	● ● ●	●	●	●	●	●	●	●	●	●	●	●	● ●	●	●	●	● ●	●	●	● ●	●	●	●	●	●	●	●	●	●	●	●	●	●	●	●

●

●	●

●	●	●	●	●	●

●	●

●

●	●

●	●	●	●

●	●	●	●	●	●	●

●	●	●

●	●	●	●

● ●	●	●	●	●●	●	●	●	●	● ●	●	●	●	●	●	●

●	●	●	●	●	●	● ●	●●	●	●	●	● ●

●	●	●	●	●	●	●	●	●	●	●	●	●	●

●	●	● ●	●●	●●	● ●	●	●	●●	●	●	● ●●	●	●	●	●	●	●	●●	●	●	●	●	●	●	●●	●	●	●	●

●	●	●	●	●	●	●	●	●	●	● ●	●	● ●	●	●	●	●	●	●	●	●	● ●	●	● ●	●	●	●	●	●	●	●●	●	● ●	●	●	●	●	●	●	●	●	●

● ● ● ● ● ●● ● ●●	● ● ● ● ● ● ● ●●

●	●	● ●	● ●	●	●	●	●	● ●	●	● ●	●	●

●	●	●

●●	●	●	●	●	●	●	●	●	●	●	●	●	●	●	●	●	●	●	●	●	●	● ●	●	●	● ●	●	●	●	●	●	●	●	●

●

●	●	●

●	●	●

●	●	●	●	●

●	●	●	●	●	●	●	●	●	●	●	●	●	●	●	●	●	●	●●	●	●	●	●	●

●	●	●	●	●	●	●	●	●	●

● ●	●

●	●	●	●	●	●	●	●	●

●	●	●	●	●	●	●	●	●	●	●	● ●	●	●	●	●	●	●	●	●	●	●	● ●	●	●	●	●	●	●	●	●

●	●	●	● ●	●	●	●	●	●	●	●	●	●	●	●	●	●

●	●	●	●

● ●●

●	●	●	●	●	●	●	●	●	●

●	●	●	●	●●	● ●	●	●	●	●●	●	●	●	●	●	●	●	● ●	●	●	●	●	●

●	●	●	●	●	●	●

●●	●	●	●	●	●	●	●	●	●	●	●

●●	●	●●	●	●	●	●	●	●	●

● ● ● ● ●● ● ● ● ● ● ● ● ● ● ● ● ● ● ●● ● ● ●● ●●

●	●	●	●	●	●	●	●	● ●	●	●	●	● ●	●	●

●

●	●	●	●	●	●●	●	●	●	●	●	●	●	●	●	●	●	●	●	●	●	● ●	●

●	●	●	●	●

●

●	●	●

●	●

●	●	●

●	●	●	●	●

●

●	●

●	●	●	●

●

●

●	●	●

●	●

●	●	●

●	●	●	●●	●	●

●●	●	●	●	●	●	●

●	●

●	●	●	●

●	● ●	●	●	●	● ●	●	●	●	●	●	●	●	●	● ●	●	●	●	●	●	● ●	●	●	●	● ●	●	●

●	●	●	●	●	● ●	●	● ●	●	●	● ●	●	●	●	●	●	●	●	●

●	●	●	●	●	●	●	●	●	●	●	●	●	●	●	●	●	●	●	●●	●	●	●	●	● ●	●	●	●	●	●	●	●	●	●	● ●	●	●	●	●

●

●	●	●	●	● ●	●	●

●	●	●

●	●	●	●	●	●	●	●	●	●	●	●	●	●	●	●

●	●

●

●	●	●	●	●	●

●	●●	●	●	● ●	●	●	●	●	●	●	● ●	●	●	●	●	●	● ● ●	●	●	● ●	●	● ●	●	●

●	●	●

●	●●	●	●	●	● ●	●	● ●	●	●	●	●	●	●	●	●	●	● ●	●	●	●	●●	●	●

●	●	●	●	●	●	● ●	● ●	●	●	●	●	● ●	●	●	●	●	●	●	●	●	●	●● ●	●	● ●	●	●

●

●	●	●	●	●	●	● ●	●	●	● ●	●	●	●	●	●	●	●	●	●	●	●	●	●	●	●	●	●	●	●●	●●	●	●	●	●●	●	●	●	●	●	●	●

●

●

● ●	●	●

●

●	●	●	●	●

●	●	●

●	●	●	●	●	●

●	●

●	●	●	●	●	●	●	●	●	●	●

●

●	●●	●	●

●	●	●	●	●	●	●	●	●	●●	●	●

●	● ●	●	●	●	●	●	●	●	●	●

●	●	●●	●	●	●	●	●	●	●	●	●	●	●

●	●	●	●

●	●	●	●	●	●	●

●

● ●	●	●	●

●	●	● ● ●	●	●	●	●	●	●	●	●

●	●	●	●	●

●●	● ●	●	●	●	●	●	●	●●	●

●

●	●	●	●	●	●	●	●	●	●	●	●	● ●	●	●	●●	●●	●	●	●	●	●	●	●	●	●	● ● ●	●	●	●●	●	●	●●	●	● ●	●	●	● ●●	●	● ●	●	●

● ●

●	●●	● ●

●	●	●	●

●	●	●	●	●	●	●	● ●	●

●	●	●	●	●	●	●	●	●	●	●	●	●	●	●	● ● ●	●	●	●	●	●	●

●	●	●	●	●

●	●

●	●	●	●	● ●	●	●	●	●	●	●	●	●	●	●	●	●	●	●	●	●	●	●	●	●	●	●	●	●	●	●	●	● ●	●	●	●	●	●	●	●	●	●	●	●	●	●	●	●	●

●	●

●	●	●

●	●	●

●

●	●

●

●

●

●	●	●	●	●	●	●	●	●	●	●	●	●	●	●	● ●	●	●	● ●	●	●●	●	●	●	●	●	● ●	●	●	●	●	●	●	●	●●	●	●

●	●	●

●

●	●	●	●●	●	●	●	●	●	●	●	● ●	●	●	●	●	●●	●	●	●	●	●	●	●	●	● ●	●	●	●

●	●

●	●	●	●

●	●	●	●	●	●	●●	●	●	●	●	●	●	●	●

● ●	●	●	●	●	●	● ●	●	●	●	●	●	●	●●	● ●	●	●	●	●	●	● ●	●	●	●	●	●	● ●	●	●	●	●	●	●	●	●

●	●	●	●	●	●	●	●	●	●	●	●	●	●	●	●	●	●	●	●	●

●	●	●	●	●	●	●	● ●	●	●	●	●	●	●	●	●	●	●

●●	●	●	●●	●	● ●	●	● ●	●●	●	●	●

●	●

●	●

●	●

●

●	●	●	●	●

●	●	●

●	●	●	●●	●	●	●	●●	●	●	● ●	●	●	●	●	●●	●	● ●

●	●	●	●	●	●	●	●	●	●	● ●	●	●	●	●	●	●	●

● ●	●	●

●	●	●	●	●	●	●	●	●	●

●	●	●

●

●

●	● ●	●	●	●	●	●	●	●

●	●	●	●	●	●	●	●	● ●	●	●	●	●	●	●	● ●	●	●	●	● ●	●	●	●	●	●	●	●	●	● ●	●

●	●

●

●	●	●

●	●

● ●	●

●	●

●

●	●	●	●	●	●	●	●	●	●● ●	●	●	●	●

●	●	●	●	●	●	●	●	●	●	●	●	●	●	●	●	● ●	●	●	●	●	●	●	●	●	●	●

●●	●	●	●●	●	● ● ●●	●	● ●	● ●

●	●	●

●	●	●	●	●	●

●	●

●	●	●	●	●	●	●	●	●	●	●	●	●	●	●

●	●	●	●	●	●

●	●	●	●

●

●

●	●	●	● ●	●	●	●●	●	●	●	●	●	●

●	●

●

●	●	●	●	●	●	●	●	● ●	●	●	● ●	●

●	●	●	●	●	●	●	●	●	●	●	●	●	●	●	●	●

●	●	●	●	●	● ●	●	●

●	● ●

●	●●	●	●	●	●

●	●	●	●	●	●●	●	●	●	●	●	●	●	●	●	●	●	●	●	●	●	●	●	●	●	●	●	●	●	●	●	●	●	●	●	●	●	●	●

●

●	●

●	●	●	●	●	●	●	●	●	●	●	●	●	●	●	●

● ● ● ●● ● ● ● ●●

●	●	●	●	●	●	●

●	●	●	●	●	●	●	●	●	●	●	●	●	●	●	●	●	●	●	●	●	●

●	●●	●	●	●	●	●	●	●

●	●	● ●	● ●	●	●	● ●	●

●

●

●	●	● ●	●	●	●	●	●	●	●

●

●	●	●	●

●	●	●	●	●	●	●●	●	●	●	●

●	●	●	●	●	●	●	●

●

●

●	● ●	●	●	●	●	●	●	●	●	●

●	●	●	●	●	●	●

●	●

●	●	●	●	●	● ●	●	●	●

●

●	●	●	●	●	●	●	●	●	●	●	●	● ●	● ●	●	●	●●	●	●	●	●	●	●	●	●	● ●	● ●	●

●

● ●	●	●	●	●	●	●	●	●	●	● ●	●	● ●	●	●	●	●	● ●	●	●	●	●	●	●	●	●

●

●	●

●	●

●●	●	●	●	●	●	●	●	●	●	●	●	●	●	●●	●	●	●	●	●	●	●

● ●	●	● ●	●	●	●	●●	●	●	●	●

●	●

●	●	●

●	●

●

●	●	●	●	●	●	●	●	●	●	● ●	●	●	●	●	●	●	● ●	●	●	●	●	●	● ●	●	●

●	●

●	●	●	●	●	●

●	●	●	●	●	●	●	●	●	●	●

●	●	●	●	●	●	●	●

●	●

●	●	●	●	●	●

●	● ●	●	●	●	●

●	●	●	●	● ●	●	●●	●	●	●	●	●	●	●	●	●	●	●	●	●	●	●	● ●	●	●	●	●	●	●	● ●	●	●	●

●	●	●

●	● ●●	● ●●	●	● ●	●	● ●	●	●	●●	●	●●	●●	●	● ●	●	●	●	●	●	●	● ●	●	●	● ●	●	●	●	●	●●●	●	●	●	●	●

●

●	●

●	●	●

●	●	●

●	●	●	●	●

●

●	●	●	●	●

●	●	●

● ●	● ● ●	●	●	●	●	●	●	●	●	●

●	●	●

●	●	●	●	●	●●	●	●	●	●	●	●	●	●

●●	●	●	● ● ● ●	●	●	●	●	●	●	●	●	●	●	●	●	●	●	●	●	●	●	●	●	● ●	●	● ●	●	●●	●	● ●	●	●	●	● ●

●

●	●	●

●	●	●	●	●

●●	●	●	●	●

●	●	●	●	●	●	●	●	●	●	●

●●	●	●	●	●	●	●	●	●	●●

●	●	●	●	● ●●	● ●	●	●	●	●	●	●	●	●	●	●	● ●	●	● ●

● ●	●	●	●	●	●	●	●	●	●	●	●	●	●●	●	●	●	●	●	●	●	●	●	●	●	●	●	●	●	●	●

● ●	●●●	●

●	●	●	●	●	●	●	●	●	●	●	●	●	●	●	●	●	●	●	● ●	●	●	● ● ●

●

●	●	●	●	●●	●	●	●	●	●	●	●

●	●	●●	●	●	●	●●●	●●●	●	●	●	●	●●	●	●	●	●	●	●	●	●	●	●	●	●	●	●●	●	●●	●	●	●	●	●	●	●	●	●	●	●	●	●

●	●	●	●	●	●	●	●	●	●	●	●	●	●

●	● ●	●	●	●	●	●	●	●	●	●	●	●	●	●

●	●	●	●	●	●	●	●	●	●

●	●	●	●	●	●	●	●	●	●

●

●	●	●	●

●	●	●	●	●

●	●	●	●	●	●	●	●	●	●	●	●	●	● ●	●	●

●	●	●	●	●	●	●	●	●	●	●	●	●	●	●	●	●	●	●	●	●	●	●	●	●	●	●	●	●	●	●	●	●	●	●	●	●	●	●	●	●	●	●	●	●	● ●	●	●	●

●	●	●

●

●	●	●●	●	●	●	●	●	●●	●	●	●	●	●	●	●	●

●	●	●	●	●	● ●	● ●	●	●	●	●	●

●	●	●	●	●

●	●	●	●	●	●	●	●	●	●	●	●	●	●	●	●	●	●	●

●●	●	●	●	●●	●	●	●	●●	● ●	●	●	●	●	●●	●

●	●	●	●	●	● ●	●●	●	●	●	●●	●	●	●	●	● ●

●	●	●	●●	●

●

●

●	●

● ●	●	●	●	●	●	●	●	●	●	●	●	●	●	●	●	●	●	● ●	●●	●	●	●	●	●	●●	●	●

●	●

●	●	● ●	●	●	●	●	●	●	●	●

●	●	●	●	●	●	●	●	●	●	●	●	●	●	●	●	●	●	●	●	●	●	●	●	●	●	●	●	●	●	●

●	●	●	●	●	● ●	●	●	●	●	●	●	●	●	●	●	●	●	●	●	●	●	●	●	●	●	●	●	●	●	●	●	●	●	●	●	●	●	●	●	●

●	●	●	●	●	●	●	●●	●	●	●	●	●●	●	●	●	●	●	●	●	●	●	●	●	●	●	●	●	●	●	●	●	●	●	●

●	●

●

●	●

●

●	●	●	●	●

●	●	●	●	●

●	●	●	●	●	●	●	●	●	● ● ●	●	●	●	●	● ●	●

●	●	●	●	●	●

●	●	●	● ●	●

●	●

●	●	●	●	● ● ●	●	●	●●	●	●	●	●	●	●	●	●	●● ●	● ●	●	●	●	●	● ● ●	●	●	●●	● ●	●	●	●	●	●	● ●	● ●	●	●	●	●	●	●	●	●	●	●	●	●	●

●	●	●	●

●	●	●	● ●	●	●	●	●	●	●	● ●	●	●	●	● ●	● ●	●	●	●	●	●	●●	●

●	●	●	● ●	●	●	● ●	●	●	●	●

●	●

●	●

●

●	●	●	●

●	●	●	●● ● ●	●	● ●	● ●●	●	●●	●● ●	●●	● ●	●	●	●	●	●	●	●	●	●

● ●	●	●	●	●	●	●	●	● ●	● ●	●	●	●	●	●	●	●	●	●	●	● ●	●	●	●	●	●	●	●	●	●	●	●	●	●	●

●	●	●	●	●	●	●	●	●	●	●	●	●	●	●	●	●●	●	●

●	●	●	● ●	●	●	●	●	●	●	●	●	●	●	●	● ●	●	●	●	●●	●	●	●	●	●	●	●	●	●	●	● ●	●	●	●	●	●	●	●	●	● ●	●●	●

●

●

●	●	●	●	●	●	●	●	●	●	● ●	●	●	●	●	●	●	●	●	● ●

●	●	●	●	●	●	●	●	●	●	●	●●	●●	●	●	●	●	●	● ●	●	●

●	●

●

●	●	●	●	● ●	●

●	●	●	●	●	●	●	●	●	●	●	●

●	●	●	●	●	●	●	●	●	●●	●	●

●	●	●

●●	●	●	●

●	●	●	●	●	●	●

●	●	●	●	●	●	●

●	●●	●	●	●	●	●	●	●	●	●	● ●	●	●	●	●	●	●	●	●	●●	●	●●	●	●	●	●	●	●	●	●	● ●	●	● ● ●	●	●	●	●	●	●	● ●	●	●	●	●	●	●

●

●

● ●	●

●	●	●

●	●

●●	●	●	●	●	●

●	●	●

●	●	●	●	●	●	●

●	●	●	●	●	●	●	●

●	●	●	●	●	●	●	●	●	●	● ●●	●	●	● ●	●	●	●	●	●	●	●	●	●	●	●	●	●	●	●	●	●	●	● ●	●	●	●	●

●	●	●	●	●

●	●	●

●	●	●	●	●	●	●	●	●	●	●

●

●

●

●	●

●	●●	●	●	●	●

●	●	●	●	●	●

●	●	●	●	●	●	●	● ●	●	●	● ●	●	●	●	●	●	●	●	●

●	●	● ●	●●	●	●	●	● ●	●	●	●	●●	●	●	●	●	●	●	●	●	●●	● ●	●	●	●	●	●	●	●	●	●	●	●	●	●	●	●●	●	● ●	●	●●	●	●	●	●	●	●	● ●	●	●

●	●

●	●	●	●	●	●	●	●	● ●	● ●	● ●	●	●	●	●	●	●●	●	●	●	●	●	●	●	●	●	●	●	● ● ●	●	●

●	●	●	●	●	●

●	●	●

●	●

●	● ●	●	● ●	●	●	●

●	●	●	●	●	●	●	●

●	●	●	●

●

●	●

●	●

●	●	●	●

●	●	●	●	●	●	●● ●	● ●	●	●	●	●	●	●●	●	●	●	●

● ●	●	●	●	●	●	●

●

●	●	● ●	●	●	●	●	●	●	●	●	●	●	●	●	●

●	●●	●	●	●	●	●	●	●●	●	●	●	●

● ●	●	●	●	●	●	●	●	●	● ●	●	●	●	●	●	●	●	●	●	●	●	●	●	●	●	●	● ●	●	●	●	● ●	●	●

●●	●	●	●	●●	●	●	●	●	●	●	●

●	●	●	●	●	●	●	●	●	●

●	●	●	●	●	●	●	●	●	●	●	●	●	●	●	●	●	● ●●	●	●	●	●	●	●	●	●	●

● ●●

●	● ●

●

●

●	●	●	●	●	●

●	●	●	●	● ●●	●	●	●	●	● ●	●	●	●

●	●	●	●	●	●	●	●	●	●	●

● ●	●	●	●	●	●	● ● ●	●	●	● ●	●	●	●	●	●	●	●	●	●	●	●	●	●	●	● ●●	●	●	●	●

●●	●	●	●	●

●	●

●	●	●	●	●	●	●	●● ●	●	●	●	●	●	●	●	●	●	●	●	●	●	●	●●	●	●	●	●	●	●	●	●

●	●	●

●	●	●	●	●	●●	●	●	●	● ●	●	●	●	●●	●	●	●	●	●	●	●	●	●	●	●	●	●	●	●	●	●	●

●	●	●	●●	●	●	●	●	●	●	●	●	●	●	●	●	●	● ●	●	●	●	●	●	●	●	●	●	●	● ●	●	●	●	●	●	●

●	●	●

● ●	●

●	●	●

●

●	●	● ●●	●	●	●	●	●	●	●	●	●●	●	●	●	● ●	●	●	●	● ●	●	●●	●	●	●	●	●	●	● ●	●	●	●	●	●	●	●	●	● ●	●	●

●	●	●	●	●	●	● ●	●	●	●	●	●	●	●	●	●	●	●	●	●	●	●	●	●	●	●	●	●	●	●	●	●	●	●	●	●	●	●

●	●

●	●	●●	●	●	●	●	● ●● ●	●	●●	●	●	●●	● ●	●	●●	●	●	●	●	●	● ●	●●	●	●	●	●	●	●	●	● ●	●	●●	●	● ● ●	●●	●	●	●	●	●	●	●	●	●	●●	●

●	●	●	●	●	●	●●	●	●	●	●	●	●	●●	●	●	●	● ●	●

●	● ●	●	●

●	●	●	●

●	● ●

●	●	●	●

●	●	●	●

●●	●	●	●

●	●	●	●	●	●

●	●	●	●	●	●	●	●	●	●	●	●●	●	●	●	●	●	●	●	●	●	●	●	●	●	●	●	● ●	●	●	●	●

●	● ●	●	● ●	●	●	●	● ●	●	●	●	●	●	● ●	●	●	●	●	●	●	●	● ●	●	●	●	●

●	●	●	●	●	●

●	●	●	●	● ●	●	●	●●	●●	●	● ●	●	●	●	● ●	● ●	●	●	● ●	●	●	●	●	●	●	●	●	●	●	●	●	●	●	●

●

● ●	●

●	●	●	●	●	●	● ●	●	●	●	●●	●	●	●●	● ●

●	●	●	●	●	● ●	●	●	●	●	●	●

● ●●	●	●	●	●	●	●	●	●	●	●	●	●	●	●	●	●	●	●	●	●	●	●	●	●	●	●	●	●	●

●	●	●	●	●	●	● ●	●	●	●	●	●	●	●	●	●	● ●	●	●

●	●

●	●	●	●

●

●	●	●	●	●	●	●	●	●	●	●	● ●	●

●	●	●	●	●	●	●	●

●	●	●	●	●	●

●	●	●	●	● ●	●	●	●	●	●	●	●	●	●	●	●	●	●	●	●	●	●	●	●	●	● ●	●

●	●	●	●	●	●	●	●	●	●	●	●	●	●	●	●	●	●	●

●

●	●

● ● ● ● ● ● ●●	●

●	●	●	●

●	●

●	●

●

● ●	●	●	●	●

●	●	●	●	●

●	●	●	●	●	●	●	●	●

●	●	●	●	●	●	●	● ●	●	●	●	●	●	● ●	●	●	●	●	●	●	●	● ●	●	●	●	●	● ●	●	● ●	●	● ●	●	●	●	●	●	●	●	●	●	●	●	●	●	●	● ●

●	●	●	●	●	●	●	●	●

●	●	●	●	●	●	●

●

●	●	●

●	●	●

●	●

●

●	●

●	●	●●	●	●	● ●	●	●	●	●

●	●	●	●	●	●	●	●	●	●	●

● ●	●	● ●

●	●

●	●	●	● ●	●	●	● ●

●	●	●

●	●●	●	●	●	●	●	● ●	●	●	●	●

●	●

●	●	●	●	●	● ●	●	●	●	●	●	● ●	●	●	●	●	●	●	●	●● ●	●●	●	● ●	●	●	●	●	● ● ●	●	●●	●	●	●	●	●	●● ●	●	●	●	●	●

●	● ● ● ● ● ● ● ● ● ●●

●	●	●

●	●	●

● ● ● ● ● ●●

●	●	●	●	●	●	●	●	●	●

● ●	●

●	●	●	●	●	●	●	●	●	●	●	●	●	●	●	●	●	●	●	●	●	●	●	●	●	●	●	●	●	●	●	●	●

●	●	●	●	●	●●	●	●	●	●	●	● ●	●	●	●	●	●	●

●	●	●	● ●	● ●●	●	● ●	● ●	●	●	●	●	●	●	●	●	●	● ●	●	●	● ● ●	●	●	●	●	●

●	●	●	●	●	●	●	●	●	●	●	●	●	● ●

●

●

●	●	●	●	●	●

●	●	●	●	●	● ●	●

●

●	●	●	●	●	● ●	●	●	●	●	●	●	●	●	●	●	●

●	●	●

●

●	●	●●	●	●	●	●	●	●	●	●	●	● ●	●	●	●	●	● ●	●	●	● ●	●	●	●	●	●

●	●	●	●

●●	● ●	●	●	●	●	●	● ●	●	●● ●	●	●	●	●	●	●	●	●	●	●	●	●

●	●

●	●	●	●

●	●	●	●	●	●	●	●	●	●	●	●	●	●	●	●	●	●	●	●	●

●	●	●	●	● ●	●	●	●●	● ●	●	●	●	●	●	●	●	●	●	●	●

●	●	●	●	●	●	● ●	●	●	●	●	●	●	●	●	●	●	●	●	●	●

●	●	●	●	●	● ●	●	●	●	●	●	●	●	●	●

●	●	●	●	●	●	●	●	●	● ●	● ●	●● ●	●	●	●	●	●	●	●	●	●	● ●	●	●	●● ● ●	●	●	●	●	●	●	●	●	●	●	●	●	●	●	●	●

●	●	●	●

●	●	●

●

●	●

●	●

●	●	●	●	● ●	● ●	●

●	●●	●	●	●	●	●	●	●	●	●	●

●	●	●	●	● ●	●	●	●	●	●

● ● ● ● ●● ● ● ● ● ● ●●

●	●	●	●	●	●	● ●	●

●

●	●	●	●	●	●	●	●	●	●	●	●	●	●	●	●	●	●	●	●	●	●	●	●	●	●	● ● ●	●	●	● ●

●	●	●	●	●	●	●	●	●	●	●

● ● ● ● ● ● ● ● ● ● ● ● ●● ●● ● ● ● ● ● ● ● ● ● ● ● ● ● ● ● ● ●● ● ● ● ●● ●● ● ● ●● ●●

●●	●	●	●	●	●	●	●	●	●	●	●	● ●	● ●	●	●	●	●	●	●	●	● ●	●	●	●	●	●	●	●	●

●	●	●	●	●

●	●	●	●	●	●	●●	●

●

●	●

●

●	● ●	●

● ●	●	●	●	●	●

●

● ●	●

●	●	●	●	●	● ●	●●	●	●	●●	●	●	●	●	●	●	●	●	●	●●	●	● ●	●	●●	●	●	●	●	●

● ●

●	●	●	●	● ●	●	●	●	●	●	●	●

●●	●	●	●	●	●	●	●	●	●	●	●	●	● ●	●	●	●	●	●	●	●	●	●	●	●	●	● ● ●	● ●	●	●	●	●●	●	●	●	●	●	●	●	●	●

●	●●	●	●

●

●

●	●	●	●	●	●	●	●

●	●	● ●	● ●	●

●

●	●	●	●	● ●	●	● ●	●●	● ●	● ●	●	●	●	●

●	●	●	●	●	●	●●	●	●	●	● ●	●	●	●	●	●	● ●	● ●● ●	● ●	●

●	●	●	●	●	●	●	●	● ●	●	●

●	●	● ●	● ●	●	●	●	●	●	●

●	●	●	●	●	●	●	●	●	●	● ●	●	●	●	●	● ●	●	●	● ●	●	●	●	●	●	●	●	●	● ●	●	●	●	●	●	●	●	●

●	● ●

●	● ●	●	●	●	●	●	●	●	●	●● ●	●	●●	●	● ●	● ●	●	●	●	●	●	●	●	●● ●	● ●	●	●	● ●	●●	● ●	●●	●	●	● ●	●	●	●	●	●	●	●	●	●	● ●	●	●	●	●	●	●	●	●	●	●

●	●

●	●	●	●	●●	●	●	●	●	● ●	●	● ●	●	●	●	● ●	●	●	●	● ●	●	● ●	●	●	●	●	●	●	●	●	●	●	●●	●	●	●	● ●	●	●	●	●	● ●	●

●	●	●●	●	●	●	●	●	●	●	●●●

●

● ●	●	●

●	●	●	●

●	●	●	●	●	●	●	●	●	●●	●	●	●	●	●	●	●	●	●	●	●	●	●	●	●

●	●	●	●	●●	●	●●	●●	●	●	●	● ●	●	●	●	●	●	●	●	●	●	●	●	●	●	●	●

●	●	●

●	●	●	●	●	●	●	●	●	●	●	●	●	●	●	●	●	●	●	●	●	●	●●	●	● ●	● ●	●	●	● ●	●	●	●

●	●	●	●●	●	●	●	●	●

●	●	●	●	●	●	●	●	●	●	●	●	●	●	●	●	●

●	●	●	●	●	●	●	●	●	●	●	●	●	●	●	●	●	●	●	●	●	● ●	●	●	●	●

● ● ● ● ● ●●

●

●●	●	●	●●	●

●	●	●●	●	●	●	●	●	●	●	●	●	●	●	●	●	●

●	●	●

●	●	●	●	●	●	●	● ●

●	●	●	●	●	●	●	●

●	●	●	●	●

●	●	●	● ●	●	●	●●	●	●	●	●	●	●	●	● ●	●	●	●	●●	●	●	●	●	●	●	●	●	●

● ● ● ● ● ●●

●	●	●	●	●	●	●●	●	●	●	●●●	●	●	● ●	●	●	●

●	●	●	●	●	●●	●	●	●	●	●	●	●	●	●	●

●	●

●	●	●	●	●

● ●	●

●	●	●	●	●	●	●	●	● ●	●	●	●	●	●	●	●	● ●

●	●	●	●	●	●

●	●	● ●	●	●	●	●	●	●	●	●	●	●	●	●	●	●

●	●	●	●	●	● ●	●	●	●	●	●	●	●

●	●	●	●	●	●	●	●	●	●

●	● ●	●	●	● ●	●	●

●

●	● ● ●● ●● ● ● ● ●● ● ● ● ● ● ● ● ● ● ● ● ● ● ● ● ● ● ● ● ● ● ● ●●

●	●	●	●	●	●	● ●	●	●	●	●	●

● ●	●	●	●	●	●	●	●	●	●	●	●	●	●	●

●	●	●	●	●	●	●	●	●	●

●	●	●

●	●

●	●	●	●	●	●	●

●	●	● ●	●	●	●	●	●

●	●

●	●	●	●	●	●	●	●	●	● ● ●	●	●	●	●●●	●	● ●	●	●	●	●	●	●	●	●	●	● ●	●	●

●	●	●	●	●	●	●	●	●	●	●●	●	● ●	●	●	●	●	●	●	● ●	●	●	●	●	●	●	●	●	●	●	●

●	●	●	●	●

●	●	● ●	●	●	●	●

●

●

●	●

●	●	●	●	●

●

●●	●

●	●	●	●	●	●	●

●

●	●

● ●	●	● ●	●	●	●	●	●	●	●	●	●	●	●	●

●	●	●	●●	●	●	●	●

●	●	●	●	●	●	● ●	●

●

●	●	●	●	●	●	●	●	● ●	●	●	●	●	●	●	●	●	●	●	●	●	●	●	●	●	●	●	●

●

●

●	●	●	●	●	●	●	●	●	●	●	●	●

●	●	●	●	●	●	●	●	●	●	●	●	●	●	●	●	●	●	●	●	●	●

●	●	●	●	●	●	●	●	●	●	●	●	●

●	●	●	●	●	●	●	●	●	●	●	●	●	●	●	●	● ●	●	● ● ●●	●	● ●	● ●	●	●	●	● ●	● ●	●●	●	●●●●	● ●	●	●	●	●	●	● ●	●	●	●	●	●

●

●

●	●

●

● ●	●	●	●	●

●	●	● ●	●	● ● ●	●	●	●	●	●	●

●	●	●	●

● ● ● ● ● ● ● ● ● ● ● ● ● ● ● ●●

●	●	●	●	● ●	●	●	●	●	●	● ●	●	●	●	●	●	●	●	●	●	●	●	●	●	●	●

● ●	●	●	●

●	●	●	● ●	●

●

●	●	●	●	●	●	●	●	●	●	●	●	●	●	●	●	●	●

●	●	●	●	●

●	●	●

●

●

●

●	●	●	●	●

●	●

●	●	●	●	●	●	●	●	●

●	●	●	●

●	●	●	●	●	●	●	●	●	●

●	●	●	●	●	●	●	●	●●	●	●	● ●	●	●	●	●	●	●	●	●	●	●●	●	●	●	●	●	●●	●

●	●	●	●	●

●	●	●	●	●	●	●	●	●	●	●	●	●	●	● ●	●	●	● ●	●	●	●	●

●	●	●	●	●	●	●	●	●	●

●

●	●

●	●	●	●	●	●	●	●	●

●	●	●	●	●	●	●	●	●	●	●	●	●	●	●	●	● ●	●	●	● ● ● ●	●	●	●	●	●	●	● ●	●	●	●	●	●	●	●	●	●	●	●

●	●	●	●	●	●	●	●

●	●	●	●	●	●	●	●	●	●	●	●	●	●	●	●	●●	●	●	●	●	●	●	●	●	●

●	●

●	●	●	●	●	●	●	●	●	●	●	●	●	●

●

●	●	●	●	●	●	● ●	●	●●	●●	●	●	●	●	●	●	●	●	●	●	●

●	●	●

●	● ●	●	● ●	● ● ● ●	●	●	●	●	● ●	●

●	●

●	●	●	● ●	●	●	●	●	●	●	●● ●	●	●	●	●●	●●	●

● ●	●	●	●	●	●	●	●	●	●	●	●

●	●	●	●	●	●	●	●	●	●	●

●	●	●

●	●

●	●●	●	●	●	●

●	●

●	●

●●	●	●	●	●	●	●	●	●	●	●	●	●

●

●	●	●	●	●	●	●	●	●	●	●	●	●	●	●	●

●	●	●	●	●	●	●	●	●	●	●	●	● ●	●	●	●	●	●	●	●

●

●	●

●	●	●	●	●	●

●	●

● ●	●	●	●	● ●	● ●	●	●	●	●	●

●	●	●	●	●	●

●	●	●	●	● ●	●	●	●	●	●	●

●	●	●	●	●	●	●	●

●	●	●

●	●	●	●●	●	●	●	●	●	●	●	●	●	● ●	●	●	●	● ●	●	●	●	●	●	●	●	●	●	●	●

●	●	●	● ●	● ●	● ●	●	●	●	●	●	●	●	●	●●	●	●	●	●	●	●	●

●	●	●

●	●	●●	●●	●	●	●	●	●	●	●	●

●	●	●

●

●	●

●●	●

●

●	●	●	●	●	●	●	●

●	●	●	●	●	●

●	●	●	●

●	●	●	●	●	●	●

●	●

●	●	●	●	●	● ●	●	●

●	●	●	●	● ●	●	●	●	●	●● ●	●	●	●

●	●	●	●	●	●

●	●

●	●	●	●	●	●	●	●●	●	●

●	●	●	●

●	●	●	●	●	●	●	●	●	●	●	●	●	●	●	●	●	●	●	● ●	●	●	● ●	●	●	●

●	●	●	●	●	● ●	●	●	●	●	●	●	●	●

●	●	●	●

●	●

●	●	●	●	●	● ●	●	●	●	●	●	●	●

●	●	●	●●	●	●	●	●	●	●	●	●	●	● ●	●	●	●	●	●	●	●	●	●	●	●●	●	●	●	●

●

●	●	●	● ●	●	●	●	●	●	●	● ●	●	●	●	● ●	●	● ●	●	●	●	●

●	● ●

●	●	●	●	●

●	●	●	●	●	●	●●	●	● ●	●	●	●	●	●	●	●	●	●	●	●	●

●

●

●	●	●	●	●	●	●	●

●	●	●	●	●	●	●	●●	●	●	● ●	●	● ●	●	●

●	●	●	●	●	●	●	●

●	●	● ●	●	● ●	●

●	● ●	●	●	●	●	●	●	●	●	●	●	●	●	●	●	●	●	●	●	●	●	●	●	●	●	●	●	●	●	●	●

●	●	● ●	●	●	●	●	●	●	●	●	●	●	●

●	●

●	●●	●	●	●	●	●	●	●	●	●	●	●	●

●	●	●	●

●	●	●	●	● ●	●	●	●	●

●	● ●	●	●	● ●	●	●	●	●●	●	●	●	●	●	●	●

●	●	● ●	●	●	●	●	●	●	●	●	●	●	●	●	●	●	●	●	●

●	●	●	●	●	●	●	●	●	●	●	●	●	●	●	●	● ●	●	●	●	●	●	●	●

●

●	●	●	●	●

●	●	●

●	●	●	● ●	●	●	●	●	●	●	●	●	● ●	●	●	●	●	●	●	●	●	●

●

●

●	●	●	●

●	●	●	●	●	●	●	●	●	●	●	●	●	●

●

●	●	●	●	●	●	●	●	●

●	●	●	●	●	●	●	●

●

●	●	●	●	●	●

●	●	●	●	●	●	●	●	●●	●	●	●	●	●	●●	●	●	●	●

●	●	●	●

●	●	●	●

●	●	●	●	●

●	●	●	●	●

● ●	●	●	●	●	●	●	●	●	●	●	●	●	●	●	●	●	●	●	●

●	●	●	●	●	●

●	●	●	●	●	●	●	●

●	●

●	●	●	●

●	●	●	●	●	●	●

●	●	●	●	●	●

●	●	●	●	●	●	●	●	●	●

●	●●	●	●	●	●	●	●	●	●	●	● ●	●

● ●	●

● ●	● ●	●	● ●	●	●	● ●	● ●	●	●	● ●	●	●	●

●	●	●	●	●	●	●	●	●

●	●	● ●	●	●

●	●	●	●	●

●

●	●

●	●	●	●	●●	●	●

●

●	●	●

●	●●	● ●	● ●	●	● ●	●	●	●	●● ●	●	●	●	●	●	●	●●	● ●

●	●	●	●	●	●	●	●	●	●	●	●	●	●	●	●	●	●	●	●

●	●	●	●	●	●	●	●	●	●	●	●	●	●	●	●	●	●	●	●	●	●

●	●	●	●	●	●	●	●	●	●	●	●	●	●	●	●	●	●	●

●●

● ●

●	●	●	●

●

●	●	●	●	●	●	●	●	●	●	●	● ●	●	●	●	●	●

●	●	● ●	●	●	●	●	●	●	●●	●	●	●●	● ●

●	●

●	●	●

●	●	●

●	●	●	●	●	● ●	●

●	●	●	●	●	●	●	●	●	●	●	●	●	●	●	●	●	● ●	●	●	●	●	●	●	●	●	●●	●	●	●	●	● ●	●

●

●	●	● ●	●	●	●	●	●	●	●	●	●	●	●	●	●●	●	●	●	● ●	●	●	●	●	●	●	●	●	●	●

●

●	●	●	●	●	●	●	●	●	●	●	●	● ●	●	●	●	●	●	●	●	●	●	●	●	●	●	● ●	●	●	●

●	●	●	●	●

●	●	●	●	●	●	●	●	●	●	●

● ● ● ● ● ● ● ●●

●	●	●	●	●	●	●	●	● ●

●	●	●	●	●●	●●	●	● ●	●

●	●	●	●

●	●	●	●	●	●	●	●	●	●

●	●	●	●	●	●	● ●	●	●	● ●	●	●	●	●	● ●

●

●	●	●	●	●	● ● ●	●	●	●	●	●	●	●	●	●	● ●	●	●●	●	●	●	●	●	●	●	● ●	●	● ●	●	●	●

●	●

●

●	●

●	●

●	●●	●	●	●	●	●	●	●	●	●	●	●

●	● ●	● ● ●

●	●	●	●	●	●	●	●

●	●	●	●	●	●	●	●	●	●

●●	●	●	●	●	●●	●	●	●	●	●	●	●	●	●	●	●	●	●	●	●

●

●	●	●	●	●	●	●	●	●	●	●	●	●	●	●	●	●	●	● ●	●	●	●	●	●	●	●	●	●	●	● ●	●	●	●	●	●	●	●	●	●	●

●	●	●	●	●	●

●	●

●	●	● ●	●

●	●●	●	●	●	●	●	●	●	●	●	●	●	●	●	●

●	●	●	●	●	●	●	●	●●	●	●

●	●

●

●	●	●	●	●	●	●	●	●	●

●

●

●	●	●	●●	●	●	●	●	●	●	●	●	●	●	●

●	●	●	●	●	●	● ●	●	●	●	●	●	●	●	●	●	●	●●	●	●	●	●	● ●	●	●	●	●	●	●	●	●

●	●	●	●● ●	●	●●	●	●	●	●	●	●	●	●	● ●	●	●

●	●	●	●	●	● ●	●	●	●	●	●	●	●	●	●	●	●●	●	●	●	●	●	●	●	●	●

●	●	●	●

●

●

●	●

●	●

●	●

●	●

●	●	●

●

●	●	●

●	●	●	●

●	●	●	●	●	●	●	●	●	●	●	●	●	●	●●	●	●	●	●	●●	●	●	●	●	●	●	●	●	●	●	●	●	●	●	●	●

●	●	●

●	●

●

●

●	●	●	●

●	●	●

●	●

●	● ●●

●	●	●	●	● ●	●	●

●	●	●	●	●	●	●	●	●	●	●

●	●	●	●	●

●	●	●	●	●

●	●	●

●	●	●	●	●	●	●	●●	●	●	●	●	●	●	●	●	●	●

●	●	● ●	●	●	●	● ●	●	●	●	●	● ● ●	●	●	●

●	●	●	●	●	●	● ●	●

●

●	●	●	●	●	●	●

●

●	●	●	●	●	●	●	●

●	●	●	●	●

● ●●	●	●	● ●	●	●●	●

●	●

●	●

●	●	●	●	●	●	●	●	● ●	●	●	●	●	●	●

●	●	●	●	●	●	●	●	●	●	●	●	●	●	●	● ●	●	●	●●	●	●	● ●	●	●	●

●	●	●	●	●	●	●	●	●	●

●	●	●	●	●	●	●	●●	●	●	●	●	●	●	●	●	●● ●●	●	●	●	● ●	●	●	● ●	●	●	●	●	●	●	●

●	●	●	●	●	●	●	●	●	● ●	● ●	●	●	●	●	●	●	●	●	●	●

●	●

●	●	●	●

● ● ● ● ● ●●

●

●	●	●	●	●	●	●	●

● ●	●

●	●	●	●

●	●

●	●	●

●	●	●	●	●	●	● ●	● ●	●	●	● ●	●	●	●	●	●●	● ●	●	●	●	●

●

●	●	●	●	●	●	●	●	●	●	●	●	●	●	●	●	●	●

●	●

●●	●	●	●	●	●	●	●	●	●●	●	●	●	●●	●	●	●●	●	● ●	●	●	●	●	●	●	●	●	●

●	●	● ●	●	●	● ●

●	●	●	●●	●	●	●	●	●	●	● ●

●	●	●	●

●	●	●	●

●	●	●	●

●

●	●	●	●●	●	●	●	●	●	●	●

●	●

●	●	●	●	●	●	●	●

●	●	●	●	●	●	●	●	●	●	●	●	●●	●	●	●	●	●

●	●	●	●	●	●	●	●	●	●	●

● ● ● ● ● ● ● ● ●●

●	●	●	●

●

●	●	●●●	●	●	●	● ●	●	●	●	●	●	●	●	●	● ●	●	●

●	●	●

●	●	●	●	●	●

●	●

●

●	●	●	●	●	●	●	● ●	●	●	●	●	●●	●	●	●	●	●	●	●	●	●	●	●	● ●	●

●

● ●

●	●

●	●	●	●	●	●	●	●	●	●	●	● ●	●	●	●	●	●	●	●	●	●	●	●	●	●	●	●	●	● ●	●	●	●	●	●	●	●	●	●●	●	●	●	●	●

●	●

●	●

●	●	●	●	●

●	●	●	●	●	● ●	● ●	●	●	●	●	●	●	●	● ●	●	●	●

●

●	●	●

●	●	● ●	●	●●	●	●	●	●	●

●	●	●	●

●

●	●	●	●	●

●	●	●	●	●	● ●	●	●	●	●	●●	●	●	●	●

●	●	● ● ●	●	●	●	● ●	●	●	●

●	●	●	●	●

●

●	●	●	●	●	●	●	●	●	●	●	●	●	●	●	●	●	●

●	●	●	●	●	●	●	●	●	●	●	●	●	●

●	●	●	●	●	●	●	●	●	●	●	●	●	●	●	● ●●	●	●	●	●

●	●	●

●	●	●	●	●	●	●	●	●	●	●

●	●	● ●

●

●

●

●	●	●	●

●	●	●	●	●	●	● ●

●	●	●	●	●	●	●	●	●	● ●	●	●	●	●	●	●	●	●	● ● ●	●	●	●

●

●	●

●	●	●	●	●	●	●	●

●

● ●	●

●	●	●

●

●	●	●	●	●

●	●	●	●

●	●	●	●	●	●	●	●	●	●	●	●	●	●	●	●	●	●	● ●

●	●	●	●	●	●●	●	●	●● ●	●	●	●

●	●	●	●	●	●	●	●	●	●	●

●	●	●	●	●	●	●	●	●	● ●	● ●	●	●	●	●	●	●	●

●

●	●	●	●	●	●	●

●	●	●	●	●

●

●	●	●	●

● ●

●

●	●

●	●	●	●	●	●	●	●	●

●	●	●	●	●

●	●	●	●	●	●	●	●	●	●	●	●	●	●	●	●	●	●	● ●	●	●	●●	●	●	●	●	●	●	●	●	●●	●	●	●

●	●	●	●

●	●

●

●●	●	●	●	●	●	●	●	●	●	●	●	●	●●	●	●	●	●●	● ●	●	●	●	●	● ●	●	●	●

●

●	●	●	●	●	●	●	●	●●	●	●	●	●	●

●	●	●	●	●	●	●	●	●	●	●	●	●	●	●	●

●	●	●	●	●

●	●	●	●	●	●●	●	●	●	●	● ●	●	●	● ●	●	●	●	●	●	●

●	●	●	●	●

●	●	●	●

●	●	●	●

●

●	●

●	●	●

●	● ●

●	●

● ●	●●	●	●	● ●	●	●	●	●	●	●

●	●	●	●	●	● ●	● ●	●	●	●

● ● ●●

●	●	●	●	●	●	● ●	●	●●	●

●	●	●	● ●	●	●	●	●	●	●	●	●	●	●	●	●	●	●	●	●

●	●	●	●

●

●	●	●	●	●	●

●

●

●	● ●	●	●

●	●	●	●

●	●	●	●

●	●	●	●

●	●	●	●	●	●	●

●	●	●	●	●●	●	●	●	●	●	●	●	●	●	●	●

●	●	●	●	●

●	●	●

●	●

●	●	●	●	●

●	●	●	●

●	●

●	● ●

●	●	●

●	●	●	●	●

●	●	●	●	●

●	●	● ●	●

●	●	●	● ●	●	●	●	●	●	●	●	●	●	●	●	●

●	● ●	●

●	● ●	●	●	●	●

●	●	●●	●	●	●	●

●	●	●	●	●	●

●	●	●	●	●

●	●	●

●	●

●

●	●	●

●

●	●	●

●	●	●	●●	●	●	●	●	●	●	●	●	●	●	●	● ●	●

●	●	●

●	●	●	●	●	●

●	●

●

●	●	●	●	●

●	●	●	●

●	●	●	●	●

●	●	●	●	●	●	●	●

●	●	●	●	●

●	●

●	●	●	●	●	●	●	●	●	● ●	●	●

●●

●	●	●	●●	●	●	●	●	●

●	●	●	●

●	●	●	●	●	●	●	●	●	●

●	●	●	●	●	●	●	●	●	●

●●

●● ●	●	●	● ●	●	● ●

●	●	●	●	●	●

●	●	●

●

●

●	●	●

●

●	●	●

●	●	●

●	●	●	●	●	●	●	●	●	●	●	●	●	●	●	●

●	●	●	●	●	●	●	●	●	●	●	●	●	●

●	●

●	●	●	●

●	●

●	●	●	●	●

●	●	●	●	●●	●	●	●	●	●	●	●	●	●	●	●

●	●	●	●	●	●	●	●	●	●	●

●

●	●

●

●

●	●	●	● ●	●	●

●

●	●	●	●	●	●

●●	●

●	●	●

●	●	●	●	●	●	●	●

●	●

●	●

●	●	●	● ●	●	●	●	●

●

●

●	●

●	●

●	●

●	●	●

●	●	● ●	●	●	●	●

●	●	●

●	●	● ●	●	●	●	●	●

● ●	●	●	● ●	●	●	●	●	●

● ● ● ●●

● ●	●	●

●	●

● ●	●	●

●	●	●	●	●	●	●

●	●	●	● ●

●

●	●	●	●●	●	●	●	●

●	●

●	●	●	●

●	●	●	●	●	●	●	●

●	●	●

●	●	●	●	●	●	●

●	●	●	●	●	●	●	● ●●	●	●	●

●

●	●

●	●	●

●	●	●	●	●	●	●	●

●	●

●	●	●	●	●	●

●	●

●	●	● ●	●	●	●	●	●	●	●

●

●	●	●	●	●	●	●	●	●

●	●	●	●	●

●	●

●	●	●	●	●	●	●	●	●

●	●	●	●	●	●	●

●	●	●	●	●	●	●	● ●

●	●	●	● ●	●	●	●	●●	●	●	●	●	●	●	● ●	●	●	● ●	●	●	●

●	●	●	●

●	●

●

●	●	●	●	● ●	●	●	●	● ●	●

●	●	●	●	●	●

●

●

●	●	●

●

●	●	●	●	●

● ●	●	● ●	●	●	●

●	●	●	●	●

●

●	●	●	● ●

●	●	●	●	●

●	●	●	●	●	●	●	●●	●	●

●	● ●	●	●	●

●	●

●	●	● ●	●	●	●	●	●

●	●	●	●	●	●	●	●

●	●	●	●	●	●

●	●

●	●	●

●	●

●

●	●

●

●

●	●	●	●	●	●●	●	●

●	●	●	●	●	●	●	●	● ●	●	●	●	●	●

●	●	●	●●	●

●	●	●

●

●	●	●●	●	●

●	●	●	●	●	●

●

●	●	●	●	●

●

●	●	●

●	●	●	●	●	● ●●	●	●

● ●	●	●	●

●	●	●	●	●	●

●	● ●	●	●

●	●	●	●	●

●	●	●	●	●	●	●

●

●

●	●	●	●

●●	●	●	●	●

●	●	●	●	●	●	●	● ●	●	●	●	●	●	●

●	●	●	●	●	●	●	●	●	●	●	●	●	●	●

●

●	●	●	●	●	●	●	●	●	●	●	●	●

●

●	●	●	●	●

●	●	●	●●	●	●	●	●●	●

●	●

●

●	●	●

●

●	●

●	●	●	●	● ●	●	●	●	●	●

●	●	●	●	●	●	●	●	●	● ●	●	●	●

●

●	●

●	●	●

●	●	●	●	●

●	●●	●●	●	●	●●	●

●	●	●

●

●	●

●	●	●	●

●	●	●	●

●

●	●	●	●

● ●	●	●	●	●	● ●	●	●	●

●	●

●

●

●	●	●	●	●	●

●	●	●	● ●

●

●	●	●	●	●	●

●	●	●	●	●

●	●

●	●	●

●	●	●	●	●	●

●	●	●

●

●

●

●	●

●	●	●	●

●	●	●	●	●

●	●

●	●	●	●	● ●	● ●	●	●	●	●	●	●	●	●	●	●●	●	●

●	●	●	●	●	●	●

●

●	●	●	●	●	●	●	●

●

●

●

●	●

●

●	●

●	●	●

●	●	●	●	●	●

●	●	●	●	●	●	●	●

●	●	●	●	●	●	●	●	●	●

● ●	●	● ●

●	●	●	●	●	●	●

●	●	●	●	●	●	●	●

●	●

●

●	●

●	●	●	●	●	●

●

●

●

●	●	●	● ●●

●	●	●	●	●	●

●	●	●	●	●	●	●	●

●	●	●

●	●	●	●	●

●	●	● ●	●	●	●

●

●

●	●	●

●

●

●

●	●

●	●

●	●	●	●

●	●	●	●

●	●	●	●	●	●	●	●

●	●	●

●	●

●	●	●	●

●

●	●

●	●	●	●	●	●	●	●	●	●

●	●	●

●	●

●

●	●

●

●	●	●

● ●	●	●	●	●

●	●

●	●	●	● ●

●

●	●

●

●

●	●

●	●	●	●	●	●	●	●	●	●	●

●	●	●

●	●	●	●	●	●	●	●	●	●	●	●	●	●	●	●	●	●	●

●

●	●	●	●	●	● ●	●

●	●

●

●

●

●

●

●●	●	●	●	●	●

● ●●

●

●

●	●	●

●	●	●	●	●

●

●	●

●	●	●	●

●

●	●	●●	●	●	●	●	●	●

●	●	●	●	●	● ●	●

●	●	●	●	●	●	●

●	●	●

●

●	●	● ●	● ●	●

●	●	●	●	●	●	●

●	●●	●

●	●	●

●

●	●

●

●	●

●

●	●	●	●

●	●	●	●	●	●	●	●	●

●

● ●	●	●

●	●

●	●	●	●

●

●

●	●	●	●	●	●	●	●

●

●	●	●	●	●

●	● ●	●

●	●

●	●	●●	●	●

●	●	●	●	●

●

●	●

●	●	●	●	●	●	●	●

●	●	●	● ●	●	●

●	●	●	●	●	●

●	●	●

●

●	●

●	●	●	●	●

●

●

●

●

●	●

●

●	●	●

●	●	●	●	●

●	● ●	●

●

●●	●	●	●	●	●	●	●	●	●	●	●

●

●

●

●

●

●

●

●

●	●

●

●	●	●	●

●	●

●

●

●

●	●	●	●	●	●	●	●	●	●

● ●● ● ●● ●●

●	●

●

●

●	●

●

●

●	●

● ● ● ●●

●	●

●	●	●	●	●

●	●	●	●	●

●

●

●	●	●	●

●

●

●

●	●	●	●

●	●	●	●

●●

●	●	●	●

●	●	●	●

●

●	●

●

●	●

●	●	●	●	●	●	●	●

●	●	●	●	●	●

●	●	●

●	●

●

●

●

●	●	●

●	●	●	●	●	●	●	●	●	●

●	●	●	●	●

●	●	●	●	●	●

● ●

●	●	●	●

●	●	●	●

●	●	●	●

●

●	●	●

●	●	●	●	●	●

●

●	●

●	●

●

● ●	● ●	●	●

●	●	●

●	●	●	●

●	●

●●

●

●

●

●	●	●	●

●

●

●

●

●	●

●	●	●

●	●	●	●	●	●	●	●

●	●	●	●	●	●	●	●

● ●	●	●	●	●	●	●	●	●	● ●

●	●

● ●	●	●	●

●	●

●	●	●	●

●

●

●

● ●	●

●

●

●	●

●

●	●

●

●

●	●	●	●	●

●	●

●

●

●	●	●	●

●

●	●	●	●	●●	●	●	●	●

●

●	●	●	●●	●	●	●

●

●

●

●	●

●	●

●	●

●	●	●

●	●	●

●●	●	●	●

●	●	●

●	●	●

●	●	●	●	●	●

●	●

●

●

●	●	● ●

●	●

●

●

●	●

●	●	●	●

●	●	●	●

●	●

●

●	●	●	●●	●	●	●	●	●	●

●

●

●	●	●	●	●	●	●

●

●

●

●	●	●	●	●	●	●

●

●	●

●	●

●	●

●	●	●

●	●	●

●	●●	●	●

●

●	●●	●	●

●	●

●

●

●

●

●	●	●	●	●	●

●

●	●

●

●

●

●	●

●

●	●

●	●	●

●

●	●	●

●	●	● ●	● ●	●	●	●	●	●	●	●	●

●	●

●

●	●	●	●	●	●

●	●	●	●	●	●	●	●	●	●	●	●	●	●

●	●	●	●	●	●	●	●	●	●	●	●	●	●	●	●	●	●	●	●	●	● ●	●	●

●● ●

●	●

●	●	●	●	● ●	●

●	●	●

●

●

●	●

●	●

●

●	●	●	●	●

●	●	●	●	●	●	●	●	●	●	●	●

●

● ● ●	●

●	●	●	●	●	●	●

●	●	●

●	●	●	●

●	●	●	●	●	●	●●	●	●	●	●

●	●	●	●	●	●	●	●	●

●	●

●

●

●

●

●

●

● ●●

●	●	●	●

●	●●	●	●

●	●	●	●	●	●	●●	●	●

●	●	●	●	●	● ●	● ●

●

●

●	●●	●	●

●	●	● ●	●	●

●	●

●	●	●

●

●	●	●	●

●	●	●	●	●	●	●	●	●	●	●	●

●	●	●	●	●	●	●●	●	●

●

●	●●	●	●

●	●	●	●	●	●	●	●	●	●	●	●	●	●

●	●	●	●	●●	●	● ●	●	● ● ●	● ●	●	●	●	●

●	●

●	●	●	●	●	●	●

●	●	●	●	●●	●	●

●	●	●	●	●	●

●

●	●	●	●	●	●	●	●	●	●	●	●	●	●

●	●	●	●	●	●	●	●

●	●	●	●●	●	●	●	●

●	●

●	●	●	●	● ●	●

●	●	●	●

●

●

●

●	●	●

●●	●	●	●	●	●	●	●	●	●	●

●	●	●	●

●	●

●	●●	●	●	●	●●	●●	●	●	●●	● ●	●	●	● ●

●	●	●

●	●

●

●

●

●	●	●	●	●	●	●	●

●	● ●	●	●

●	●

●

●	●	●	●●	●	●	●	●	●	●	●●	●	●	● ●	●	●

●

●	●	●	●	●	●	●	●	●	●	●

●	●	●	●	● ●	●	●	● ●	●	●	●	●	●	●●	●

●

●

●	●

●	● ●

●	●	●	●

●	●

●	●

●

●

●	●	●	●

●	● ●	●

●	●	●

●	●	●	●

●

●	●	●	●	●	● ●	●	●	●	●	●

●

●	●

●

●	●	●	●	●	●	●	●	●	●	●

●

● ● ● ● ● ● ● ● ●●

●	●	●

●	●	●	●	●	●	●	●	●	● ●

●	● ●	●	●	●	●

●	●

●	● ●

●	●	●	● ●	●●	●

●

●	●●	●	●	●	●	●	●	●	●

●	●	●	●	●	●●	●	●	●	●	●	●	●	●	●	●	●	● ●	●	●	●	●	●	●	●	●	●

●	●	●

●

●	●

●

●

●	●

●	●

●

●

●

●	● ● ● ● ● ● ●●

●

●	●	●	●	●

●

●

●	●

●	●

● ●

●	●	●

●	●	● ●	●	●	● ●●	●

●	●	●	●	●	●	● ● ●	●	●

●	●	●

●	●	●	●

●

●	●

●

●

●

●

●	●	●	●	●	●	●	●

●	●	●	●	●	●	●	●	●	●	●	●

●

●	●	●	●	●	●	●	●	●	●	●	● ●	●	●	●	●	● ● ●	●	●	●	●	●

●	●

●	●	●	●	●	●	●	●	●	●	● ●	●	● ●	● ●	●	●

●	●

●	● ●	●	●	●	●

●	●	●	●

●	●	●	●	●

●	●	●	●

●

●

●

●	●	●	● ●

●	●	●	●	●

●	●	●	●	●	●

●	●	●	●

●	●

●	●	●	●	●	●	●	●	●

●	●

●	●	●	●	●	●	●	●	●	●	●	●	●	●

●	●	●	●	●	●	●	●

●	●	●	●	●	●	●	●

●

●

●	●	●	●	●

●	●	●

●	●	●	●	●	●	●	●	●

●	●	●

●	●	● ●	●	●	●	●	●	●	●	● ●	●	●●	●	●	●

●	●	●	●	●

●	●	● ●	●	● ●	●	● ●	●	●	●	●●	●

●	●	●	●	●

●	●

●	●

●

●

●

●	●	●	●	●	●	●	●	●

●	●	●	●	●	●	●	●

●	●

●	●	●

●	●	●

●

●

●	●

●	●	●	●	●	●	● ●	●	●	●

●

●

●

●	●

●	●	●	●	●	●	●	●●	●	●	● ●	●	●

●	●	●	●	●	●	●	●	●	●	●	●	●●	●

●	●	●	●	●	●	●

●	●	● ●	●	●	●	●	●	●

●	●	●	●	●	●

●	●	●	●	●

●	●	●	●	●

●	●	●	●	●●	●

●	●

●	●

●	●

●	●

●	●	●

●

●

●	●	●

●

●	●	●

●	●	●	●	●	●	●	●	●	●●	●	●	●	●	●	●	●	●	●	●	●	●	●

●

●	●

●

●	● ●

●	●

●

●	●

●	●	●

●

●	●

●	●

●	●	●	●	●	●	●	●	●	●

●	●	●

●

●	●	●	●	●	● ●	●●

●	●	●	●	●	● ●	●	●	●	●	●	●

● ●	●	●	●

●	●

●

●

●

●●	●

●	●	●	●	●	●	●	●	●	●	●	●	●

●	●

●

●

●

●

●	●	●	●	●	●	●	●	●●	●	●	●	●

●	●	●	●	●	●	●	●	●	● ● ●	●	●	●	●

●	●	●	●	●	●	●	●	●

●	●

●	●	●	●	●	●	●	●	● ●	●	●	●	●	●	●	●	●	●

●●	●	●	●	●	●	●	●	●	●

●	●

●	●	●	●	●	●	●	●	●	●	●

●	●	●	●	●	●	●	●	●	●●	●●	●	●	●	●

●	●●	●	● ●

●	●	●	●	●	●	●	●●	●	●

●	●

●	●	●

●	●	●

●

●	●

●	●	●

●	●	●

●	●	●	●	●	●	● ●	●	● ●	●	●

● ●

●	●

●●	●	●	●	●	●	●	●	●	●	●	●	●

●	●●	●	●	●	● ●	●

●

●	●	●	●	●	●	●	●	●	●	●	●	●	●	●	●	●	●	●	●

●	●	●

●

●	●	●	●

●

● ● ● ● ● ● ●●

●	●	●	●	●	●	●	●	●	●	●	●	●

●

●	●	●●	●	●	●	●

●	●	●

●

● ●	●

●	●	●	●

●

●

● ●	●	●	●	●	●	●	●

●

● ●	●	●	●	●	●	●	● ●	●	●	●	●	●

●	●	●	●	●	●	●	●

●	●	●	●	●	●	●

●

●	●	●	●	●	●	●

●

●	● ●

●

● ● ● ● ● ● ● ● ●●

●

●	●	●

●	●

●	●	●

●	●	●	●	●	●	●	●	● ●●	●	●	●	●	●	●

●	●	●	● ●	●	●	●

●

●

●	●	●	●	●	●	●	●	●	●	●	●	● ●	●	●	●

●	●	●	●

●	●	●

●

●	●

●	●

●

●	●	●	●	●	●	●

●

●	●●	●	●	●	●	●	●	●	●	●

●	●	●	●	●	●	●

●	●	●

●	●	●	●	●	●●	●	●	●	●	●	●	●	●	●	●	●	●	●	●

●	●	●	●	●	●	●	●●	●	●	●	●	●	●	●

●	●	●	●	●

●	●

●	●	●	●	●

● ● ● ● ●●

●	●	●	●	●	●	●	●	●	●	● ●	●	●

●	●	●	●

●	●	●	●	●	●	●	●	●	●	●	●	●

●

●	●	●	●	●

●

●	●	●	●	●	● ●	●	● ● ●	●	●	●	●	●	●	●	●	●	●●	●	●	●	●

●

●

●	●	●

●

●

●

●● ●	●	●	●	●

●	●	●	●

●	●	●	●	●	●	●	●	●	●	●	●	●	●

●	●	●	●

●	●

●

●	●●	●	●

●

●	●	●

●

● ●	●

●	●	●	●	●	●	●	●	●	●

●	●	●	●	●	●	●●	●	●	●	●●	●	●	●	● ●	●	●	●●

●	●	●	●

●	●	●	●	●	●	●	●

●	●	●	●	● ●	●	●	●	●	●	●	●	●	● ●	●	●

●	●	●	●

●

●	●

●●

●	●	●

● ●	●	●	●	●	●	●	●	●	●	●	● ●	●	●

●	●	●	●	●	●	●	●	●	●

●	●	●	●	●	●	●	●	● ●	●	●	●	●	●	●	●	●	●	●

● ●	●	●	●	●	●

●

● ●

●

●

●

● ●	●

●	● ● ●	●	●	●

●	●	●	●	●	● ●	●

●	●	●	●	●	●

●	●	●	●	●	●	●	●	●	●	●

●

●	●

●	●	●	●	●	●	●	●	●	●	●	●	●	●	●	● ●	●	●

●	●

●

●

●	●

●	●	●	●	●	●	●	●

●

●	●	●	●	●	●	●	●	●	●

●

●	●	●	●	●	●	●	●	●	●	●	●	●	●

●	●	●	●	●	●	●	●●	●	●	●	●	●	●	●	● ● ● ●	●	●	●	●	●	●	●	●	●	●	●	●	●

●	●

●

●	●

●

●	●	●	●	●	●	●	●	●	●

●

●	●

●	●	● ●	●	●	●	●	● ●	●	●	●	●	●

●	●	●	●	●	●	●	●	●	●	●	●	●	●	●	●	●	●	●	●	●

●	●	●	●

●

●

●	●	●	●

●	●

●	●	●	●	●	●	●	●

●

●

●	●

●	●	●

● ● ● ● ● ● ● ● ● ●● ● ● ● ● ● ● ● ● ● ● ● ● ● ● ●●

●	●	●	●●	●	●	●	●	●	●	●	●	● ●	●

●	●

●	●	●	●	●	●	●

●	●	●	●	●	●	●	●	●	●	●	●	●

●

●	●	●	●	●	●	●	●	●	●	●	●	●	●	●	●

●	●	●	●●	●	●	●	●	●

●	● ●	●	●	●	●	●●	●	●	●	●	●	●	●	●

●	●	●	●	●	●	●	●

●	●	● ●

●

●	● ●	●	●●	●	●	●

●	●	●	●	●	●	●

●	●●	●	●	●

●

●	●

●	●

●	●	● ●

●

●	●	●

●

●	●

●	●	●	●	●

●

●

●	●	●	●	●	●	●	●

●	●	●

●	●	●	●

●	●

●	●	●

●

●

●	●	● ●	●● ●	●

●	●	●	●	● ●	●	●	●	●	●	●	●	●	●	●

●	●

●	●	●	●	● ●	●	●	●	●	●	●

●	●

●	●

●

●	●

●	●	●	●	●	●	●	● ●

●	● ●	●	●	●	●

●	●	●

●

●

●	●

●	●	● ●	● ●	●	●	●	● ●	●	●	● ●	●

●	●	●	●	●	●	●	●	●	●

●	●	●	●	●	●	●	●	●

●	●	●	●	●	●	●

●	●	●	●	●	●

●	●	●	●	●	●	●	●	●	●	●	●	●

●	●

●

●

●	●	●

●	●	●	●

●	●	●	●	●	●	●	●	●

●	●	●	●	●	●	●	●	●	●

●	●	●	●	● ●	●	●	●	●	●	●	●	●

●	●	●	●

● ●	●

●	●	●

●

●	●	●	●

●

●

●

●	●	●

●	●	●	●	●	●	●

●	●	●	●	●	●	●	●	●

● ● ● ● ●	●	● ● ● ● ● ● ●●

●

●●	●	●	●	●	●	●	●	● ●	●	●	●	●	●

●

●

●

●	●

●

●	●	●	●	●	●	●	●

●	●	●	●	●	●	●	● ●	●	●	●	●	●	●	●

●	●	●	●

●	●	●	●	●	● ● ●

●

●	●	● ●	●

●	●

●	●	●	●	●	●	●

●	●	●	●	●	●	●

●	●	●	●	●	●	●	●	●	●	● ●	●	● ●	●	● ●	●

●	●

●

●

●	●	●	●	●

●

●	●	●

●	●	●

●

●	●	●	●

●	●	●

●	●	●	●	●	●

●●	●	●	●	●	●	●	● ●	●	●	●	●	●	●	●	●

●	●	●	●

●	●	●	●	●

●

●	●

●	●	●	●	●	●

●

●

●	●	● ●	●

●	●● ● ● ●	●	●	●	●	● ●●	●	●●	●	●	●	●	●	●

●	●●	●	●	●	●

●	●	●

●	●	●	●	● ●	●	● ●

●	●	●	●

●

●	●

●	●	●

●	●

●	●

●	●	●

●	●

●	●	●	●	●	●	●

●

●	● ●	●	●	●	●	● ●	●	●	●	●	● ● ●	●	●	●	●

●	●	●	●	●	●	●	●	●	●	●

●

●	●	● ●

●

●

●	●	●	●	●	●

●

●

●

●	●	●

●	●	●	●	●

●	●	●	●	●	●	● ●	●	●	●	●	●

●	●	●	●	●	●	●	●	●	●	●	●	●	●

●

●

●	●	●

●	●	●	●	●

●

●	●

●	●	●	●

●	●●	●	●	●

●	●	●	● ●	●	● ●	●	●	●	●	●

●

●

●	●

●	●

●	●	●	●	●	●	●	●	●	●	●	●

●	●	●

●	●	●●	●

●

●

●

●

●	●

●	●

●●	●	●	●	●	●	●	●	●	●	●	●	●	●	●	●	●

●	●	●	●	●	● ●	●	●	●	●	●	●

●	●	●	●	●	●	●	●	●

●	● ●	●	●	●	●●	●●	●	●	●	●	●	●

●

●	●	●	● ●

●	●	●

●

●	●

●	●	●	●	●	●	●	●	●	●

●	●	●	●	●	●

●	●	●	●	●

●	●	●

●	●	●	●	●

●	●	●	●	●	●	● ●	● ●	●	●	●	●	●	●	●	●	●

●

●	●	●	●	●	●	●	●●	●

●	●	●	● ●	●	●	● ●

●

●	● ●	●	●	●

●

●	●	●	●

●	●	●	●

●	●	●

● ● ● ● ● ●●	●

●	●	●	●

●

●

●	●	● ● ●	●

●

●

●	● ●	●

●	●

● ●	●●

● ●	●	●	●	●	●	●	●	●	●	●	●	● ●	●	●	●	●	●● ●	●	●●	●	●	●	●

●	●	●	●	●

●	●	●	●	●	●	●	●	●

●

●	●

●

●	●	●	●	●

●	●	●	●	●	●	●	●	●

● ●	●	●	●	●

●	●	●

●	●	●

●	●	●	●

●	●	●

●	●	●	●	●●	●	●	● ●	●	●	●	●	● ●	●	●	●	●	●●	●

● ●	●	●

●

●	●	●	●	●	●	●

●

●	●	●	●	●	● ●	●	●	●	●	●	●	●	●	●	●	●	●

●	●	●

●	●

●

●	●	●	●	●	●	●	●	●	●	●	● ●	●	●	●●	●	●	●	●	●

●	●	●

●

●

●

●	●

●	●	●	●	●	●

●	●	●	●	● ●	●	●	●	●	●	●	●	●	● ●	●	●	●	●

●	●	●

●	●	●	●

●	●	●	●	●	●	●	●	●	●	●

●	●	●	●	●	●	●	●	●●	●	●	● ●	●	●	●●	●	●

●	●

●

●	●

● ● ● ● ● ● ●●●

●	●	●

●	●	●

●

●	●	●	●	●	●	●	●	●	●	●	●	●	●	●●	●	●	● ●	●

●	●	●	●

●	●

●

●

●	●	●	●

●	●	●	●	●	●

●

●

●

●	●	●	●	●	●

●

● ●	●	●	●	● ●

●	●	●	●	●	●	●	●

●	● ●	●	●	● ●	●	●

●	●

●	●	●	●	●	●	●	●	●	●	●	●	●

●	●	●

●	●

●

●

●	●	●	●	●	● ●	●	●	●	●	●

●	● ●	●	●	●	●

●

●	●

●	●	● ●	●	●	●	●	●	●	●	●	●	●	●	●	●

●	●	●	●	●	●

●

●	●	●

●	●

●	●	●	●	●

●●

●	●	●	●	●

● ●

●	●	●	●	●	●	●	● ● ●	●	● ●	●	●	●	●	●	●	●

●

●

●	●	●	●	●	●	●	●	●	●	●

●	●	●	●	●	●

●

●	●	●	●	●

●	●	●	●	●	●	●

●	●

●	●	●	●

●	●	●	●	● ●	●	●	●	●	●	●	●	●	●	●	●

●	●	●	●	●	● ●	●	●	●	●

●	●	●	●

●	●	●	●	●	●	●	●	●	●	●	●	●	●	●

●	●	●	●	●	●	●

●	●	●

●	●

●	●

●

●

●●	●

●

●

●	●	●	●	●	●	●	●

●

●	●	●	●

●

●	●	●

●

●	●	●	●

●	●	●	●	● ●	●	●	●	●	●	●	●

●	●	●	●	●	●	●	●	●

●	●	●

●	●	●	● ●	●	●	●	●	●	●	●	●	●

●	●	● ●

●

●	●	●	●	●	●	●	●	●	●	●	●	●

●	●●	●	●	●	●	●	●	●

●	●	●	●	● ●	●

●	●	●

●

●	●	●

●	●	●	●

●	●	●

●	●

●

●

●

●

●	●

●

●

●

●

●

● ●	●	●	●	●

● ●	●	●	●	●	●

●	●●	●	●	●

●	●	●	●	●	●

●	●	●	●	●	●	●

●	●	●	●	●	●	● ●	●	●	●	●	●	●	●

●	●	●	●

●

●

●

●	●

●

●

●	●	●● ●	●	●	●	●	●	●	●

●	●	●	●

●	●	●

●

●

●

●	●	●

●	●	●	●

●	●	● ●	●●	●	●●	●	●	●

●	●	●	● ●

●	●

●	●	●

●	●

●●	●	●	● ●	●	●

●

● ● ● ● ● ● ● ● ●● ● ● ● ●● ●●

●	●	●	●

●

●	●

●	●	●

●

●	●	●

●

●

●

●	●	●	●	●	●	●	●	●	● ●	●	●	●	●	●

●	●	●	●	●	●	●	●	●	●	●	●	●	●	●	●	●

●	●

●	●	●

●	●	●	●	●	●	●

●

●	●	● ●	●	●	●	●	●	●

●	●	●	●

●	●	●●	●	●	● ●	●	●

●	●	●

●

●	●

●

●

●	●

●	●	●

●	●

●

●

●	●

●	●	● ●	●	●	●	●	●	●	●

●	●	●	●	●	●	● ●	●	●	●	●	●	●	●	●

●	●	●	●	●	●	●	●●	●●	●	●	●	●	● ●	●	●	●	●	●	●	●	●	●

●	●●	●

●

●	●	●

●

●

●

●	●	●	●

●

●

●	●

●

●	●	●	●

●

●	●	●	●	●	●

●	●	●	●	●	●	●	●	● ●

●	●	●	●

●	●	●	●	●

●	●	●	●	●	●	●	●

●

●

●

●	●

●	●

●

●	●	●

●	●	● ●	●	●	●	●	●	●	●	●	●

●

●	● ●	●

●	●	●	●	●	●

●	●	●	●	●	●	●

●	●

●

●	●	●	●	●

●

●	●	●	●	●

●	●	●	●	●

● ●●

●

●

●	●

● ●

●

●

●	●

●	●	●	●	●●	●	●	●	●	●

●	●	●	●	●	●

●	●	●

●	●

●

●	●

●	●	●	●	●	●	●	●	●	● ●	●	●	●

●

●	●	●	●	●	●	●	●	● ●	●	●	●	●	●	●

●	●	●	●	●	●

●	●	●	●	●

●	●

● ●

●	●

●

●	●

●	●	●	●	●	●	●	●	● ●	● ●	●	● ●	●	●	●	●	●	●

●	●	●	●	●

●	●	●	●

●	●	●	●

●	●	●	●	●	●	●	●

●	●	●●	●	●

●	●

●	●	●	●

●	● ●	●	●	●

●	●

●	●

●

●	●	●	●

●	●	●

●	●

●	●	●

●

●	●	●	●	●

●

●	●	●

●	●	●	●	●	●	●	● ●	● ●	●	●	●

●	●	●	●

●	●

●	●

●

●	●	●	●	●	●	●	●	●	●

●	●	●	●	●	●	●	●	●	●

●	●	●

●

●

●	●	●	●	●	●	●	●	●

●	●	●	●	●

●

● ●	●	●	●

●	● ●	●	●	●	● ●

●	●	●	●	●	●	●	●	●	●	●	●	●	●	●	●	●	●	●●	●	●	●	●	●

●●

●	●

●	●	●	●	●

●	●

●	●	●	●	●

●	●	●	●	●	●

●	●	●	●	●	●	●	●	●	●	●	● ●	●	●

●	●	●

●

●	●	●	●

●

●	●

●	●

●	●

●	●	●	●	●

●

●	●●	●	●	●	●	●	●	●

●

●

●	●	● ●

●	●	●

●	●

●	●

●

●	●	●	●

●	●	● ●	●	●

●

●	●

●	●	●

●

● ● ● ●●

●

●

●	●

●	●	● ●	●

●

●	●	●

●	●

●	●

●	●	●	●	●	●	●	●	●

●	●

●	●	●	●	●	●	●	●	●	●	●

●	●	●	●	●	●

●	●	●	●●	●	●	●	●	●

●	●	●

●	●	●	●	●	●	●	● ●	●	● ●	●

●	●	●	● ●	●	●	●

●	●

●	●	●	●	●	●	●	●	●	●	● ●

●

●

●	●	●

●●	●	● ● ●	●	●●	●	●	●	●	●

●	●	● ●	●	●

●	●	●	●	●

●	●	●	●

●	●	●	●	●	●	● ●	●	●	●	●●

●	●	●	●	●	●	●	●	●	●

●

●	●

●	● ●●

●

●

●●	●	●	●	●	●

●	●	●	●	●	●	●

●

●	●	●	●

●	●	●	●

●	●●	●	●	● ●	●	● ●	●

●

●	●	●	●	●	● ●	●	●

●

●

●	●	●	●	●	●

●	●	●

●	●	●

●

●

●

●

●	●	●

●	●	●	●

●

●	●	●

●

● ●	●

●	●

●	●

●	●

●

●

●	●

●	●	●	●	●	●	●	●	●	●	●	●	●	●	●

●	●

●●	●	●

●	●	●	●	●	●	●	●	●	●	●	●	●	●

●	●	●	●	●

●

●	●	●	●

●

●	●	●

●	● ●	●	●

●

●	●	●	●	●	●

● ●	●	●	●	● ●	●	●	●	●	●	●

●	●	●	●	●

●

●	●	●

●

●	●	● ●	●	●

●	●	●	●	●	●	●

●

●	●	●	●

●	●	●	●

●	●

●

●

●	● ●	●	● ●	●	●

●	●	●

●	●	●	●	●●	●	●	●	●

●	●	●

●	●	●

●

●

●

● ●	●	●	●	●	●

●	●	●	●	●

●	●	●	●	●	●

●	●	●	●

●	●

●

●	●	●	●	●	●

●

●

●	●

●	●

●	●

●	●	●	●

●	●

●	●	●●

●	●	●	●	●	●	●	●	●●	●

●	●	●

●

●

●	●	●	●	● ●	●	●	●

●	●	●	●	●	●

●	●	●	●	●

●

●	●

●	●	●	●	●

●

●

●	●

●

●	●	●	●	●

●	●	●

●

●	● ●	●	● ● ●	●	●	●	●	●●	●	● ●	●

●	●	●	●	●	●	●	●	●	●	●	●	●	●	●	●	●

●	●

●●	●	●	●	●

●	●	●

●	●	●	●	●

●	●

●

●	●	●	●	●	●	●

●	●	●

●	●	●	●	●

●	●	●	●

●

●

●	●	●

●

●	●

●	●

●

●	●	●	●	●	●	●	● ●	●	●	●	●	●	●

●

●	●

● ●	●	●

●

●

●	●	●	●

●	●	●

●

●	●

●	●

●	●	●

●	●	●	●

●	●	●	●	●●	●	●	●

●	●	●

●	●	●	●	●	●●	● ●	●	●●	●	●

●	● ●	●	●	●

●	●

●	●	●	●	●

●	●	●

●

●	●

●

●

●	●	●	●

●	●

●	●	●	●	●	●	●	●

●

●	●

●	●●	●	●	●	●	●	●	●

●

●	●

●

●	●	●

●	●	●	●	●	●	● ●

●

●	●	●	●	●	●

●	●	●

●	●

●	●	●

●	●	●	●	●

●	●

●

●

●	●	●

●	●	●

●	●

●

●	●

●	●

●	●	●

●	●	●

●	●

●	●

●

●	●	●	●

●	● ●	●

●	●	●	●	●

●	●

●	●

●

●

●

●	●

●	●	●

●	●

●	●	●	●

●	●

●

●	● ●	●	● ●	●	●	●

●	●	●

●	●	●	●

●	●	●

●	●	●

●	●	●	●	●

●	●	●	●

●	●	●	●	●

●

●

●

●	●

●	●	●	●	●	●	●	●	●	●

●	●	●	●	●	●

●

●

●	●

●	●	●	●	●	●	●	●

●	●	●	●	●

●	●	●	● ●	●	●

●	●	●	●

●

●	●	●

●

●

●

●	●	●

●	●	●	●	●	●

●	●	●

●	●	●

●	●

●

●	●	● ●	●

●

●

●

●	●	●

●	●	●	●

●

●	●

●	●	●

●

●	●●	●	●

●	●	●

●	●

●

●	●	●	●	●	●

●	●

●	●

●	●	●	●	●

●	●	●	●	●	●

●	●	●	●	●	●

●

●

●

●

●

●	●

●	●	●	●	●	●	●	●

●	●

●

●

●	●	●	●	●	●	●

●

●

●

●	●

●	●	●	●	●

●	● ●	●

●

●	●	●	●

●	●

●

●

●	●

●	●	●	●	●	●	●●	●	●	●	●

●

●

●	●

●	●

●

●

●	●	●	●	●

●	●

●

●	●

●

●

●

●

●

●	●	●	●

●

●

●	●

●	●	●

●

●

●

●	●	●	●	●	●

●	●●	●

●

●

●	●

●	●	●	●

●	●	●

●	●	●

●	●

●	●	●	●	●	●	●

●

●

●

●	●	●	●	●	●

●	●	●

●	●	●

●	●

●	●	●

●	●	●	●	●	●

●	●

●

●

●

●

●	●

● ●	●

●	●

●	●	●

●●	●

●	●	●

●	●	●	●

●

●

●

●

●

●	●

●

● ●

●

●	●

●	●	●

●	●

●

●●	●	●	●	●	●

●	● ●	●	●	●	●	●

●	●

●

●	●

●

●

●

●	●	●	●	●

●

●	●	●	●

● ● ●●

●

●	●	●	●	●

● ●	●

●

●

●	●	●

●

●	●

●	●

●

●

●

●

●

●

●	●	●	●

●

●	●	●	●	●	●	●

●

●

●	●

●	●

● ●	●	●	●

●

●

●

●

●	●

●

●	●	●

●

●

●

●

●	●	●

●

● ●●

●

●	●	●●	●

●

●	●	●

●	●	●

●	●

●

●	●

●	●	●

●	●

●

●

●

●

●

●

●

● ●

●

●	●	●●	●	●	●	●

●	●	●	●	●

●

●

●	●

●	●

●

●

●

●	●

●

●	●	●	● ●	●	●	●	●

●	●	●	●

●	●

●

●	●	●	●●	●	●	●	●

●

●

●	●	●

●	●

●

●	●	●

●

●	●	●	●

●	●	●	●	●	●

●

●

●

●

●

●

●

●

●	● ●

●

●	●

●	●	●	●	●	●	●	●	●

●

●	●	●

●

●	●	●●

●

●	●

●	●	●	●	● ●

●

●

●

●	●

●

●

●

●

●	●	●

●	●

●

●	●

●	●	●

●	●

●

●

●

●

●

●

●	●	●

●

●

●	●	●	●	●

●	●	●	●

●	●	●

●	●

●

●

●	●

●	●	●	●

●	●

●	●	●	●

●	●	●

●

●

●

●	●

●	●	●	●	●	● ●

●

●	●	●

●

●	●

●

●

●	●	●	●

●	●

●

●	●

●

●	●

●

●

●	●

●

●	●

●

●

●

●

●

●	●	●	●	●	●	●	●	●

●

●	●	●

●

●	●

●

●	●	●	●	●

●

●

●

●	●

●

●

●	●

●

●	●	●

●	●	●	●

●

●

●

●

●

●

●

●	●

●

●	●	●

●	●	●	●	●	●	●

●

●

●	●

●	●	●

●

●

●

●	●

●	●

●	●	●	●

●

●

●	●	●	●	●

●

●	●

●	●

●

●

●

●	●	●

●

●

●●

●

●

●

●

●

●

●

●

●

●

●

●	●	●

●	●	●	●

●	●	●	●	●	●	●	●

●	●	●	●	●	●	●

●	●

●	●	●	●	●	●	●

●

●

●

●

●	●	●	●

●

●

●	●

●

●

●

●

●

●	●

●

●

●

●

●	●	●

●

●

●	●

●

●

●

●	●	●

●	●

●

●

●

●

●	●	●

●

●	●

●	●	●

●	●

●	●	●

●

●

●	●

● ●	●

●

●	●	●

●	●

●	●

●	●	●	●

●

●	●	●	●

●

●

●

●

●	●	●	●	●

●

0.75	0.75

0.50	0.50

Relative value

Relative value

0.25	0.25

0.00

0.00	0.25	0.50	0.75	1.00

Relative genetic effects

0.00

0.00	0.25	0.50	0.75	1.00

Relative methylation level (CpG)

1.00	1.00

●

●

●	●	●	●	●

●

●	●	●	●	●	●	●

●	●

●

●

●

●

●	●	●	●	●	●	●

●	●	●

●	●

●

●	●	●	●	●

●	●	●

● ●	●	●	●	●	●

●	● ●

●	●	●	●	●	●	●

●	●

●

●

●	●

●	●	●	●

●

●

●

●

●●

●	●

●	●

●	●	●	●	●	●	●	●	●

●●	●	●	●	●	●	●	●	●	●	●	●	●	●	●	●	●●	●	●	●	●

●	●	●	●	●	●	●	●	●	●

●

●

●

●

●	●	●

●	●	●	●	●●●	●	●	●	●	●	●	●●	●

●	●

●

●

●

●

●	●	●	●	● ●

●

●

●

●

●

●	●	●

●	●	●	●	●	●

●	●	●	●	● ●	●	●	●	●	●	●	●	●	●	●	●	●

●	●	●	●	●	●	●

●

●

●	●

● ●	●	●

●	●	●	●	●

●

●	●

●	●	●	●

●	●	●	●	●

●	●	●	●	●	●	●	●	●

●	●

●	●	●	●

●	● ●	●	●	●	●	●	●

●

●

●	●	● ●

●	●	●	●

●	●	●	●

●	●	●	●	●

●

●	●	●	●	●	●	●	●

●

●	●	●	●	●	●

●

●

●	●	●

●	●●	●	●	● ●	●	●	●	●

●	●	●	●	●

●	●	●	●	●

●	●

●	●

●	●

●	●

●

●

●	●	●	●	●	●	●

●	●

●	●

●	●	●	●	●	●

●	●	●	●	●	●	●	●

●	●	●	●	●	●●	●	●	●	●

●	●	●

●

●	●

●	●

●

●

●

●	●	●

●

●

●

●	●	●	●	●

●	●	●	●	●	●	●	●	●	●

●

●

●

●

●	● ●	●	●	●	●

●	●

●	●	●	●

●	●	●	●	●	●	●	●

● ●

●	●	●	●	●

●	●	●

●●	●	●	●

●	●

●	●	●	●	●	●

●	●	●	●

●

●	●

●

●	●

●	●	●

●	● ●

●

●

●	●

●	●

●	●	●	●	●	●	●	●	●	●	●	●	●	●

●	●

●

●

●	●	●	●	●

●	● ●	●	●	●	●	●	●	●	●

●

●	●

●	●

●	●	●	●	●

●	●	●	●	●	●

●	●	● ●

●

●	●

●	●

●	●

●	●	●	●	●

●	●	●	●	●	●●	●	●	●	● ●	●	●

●	●	●	●	●	●	●	●	●	●	●	●	●	●

●

●	●	●	●

●

●	●	●

●

●	●	●	●

●	●	●	●●	●

●	●

●

●

●	●	● ●	●	●	●

●

●	●

●	●	●

●	●	●	●	●	●	●	●	●	●	●

●	● ●	●	● ●	●

●	●	●

●	● ●	●

●	●	●	●	●	●	●	●	●

●	●

●	●●	●	●

●

●

●	●	●	●

●	●	●	●	●	●

●

●	●	●	●	●

●	●

●

●

●	●	●	●	●	●	●	●

●	●	●	●

●	●

●

●	●

●

●

●	●	●

●	●

●

●	●

●	●

●	●	●	●	●	●	●	●	●	●

● ● ● ● ● ● ● ●●

●	●	●	●	●	●	●	●	●	●	● ●●	●	●	●

●

●

●

●

●

●	●

●	●	●	●

●	●	●	●	●

●	●●	●	●	●	●	●	●	●	● ●●	●

●	●

●	●	●	●	●

●	●	●	●

●	●	●

●

●	●

●

●

●

●	●	●	●

●

●	●	●	●	●

●

●

●	●

●	●	●	●	●	●	●	●	●	●	●

●	●	●

●	●	●	●	●

●

●	●

●	●

●

●	●	●

● ●

●	●	●	●

●

●●	●	●	●	●	●	●	●	●

●	●

●	●	●	●	●

●	●	●

●

●	●

●	●	●

●	●

●	●	●

●	●	●	●

●	●	●	●●	●	●	●	●	●	●●	● ●	●	●	●	●	●	●	● ●	●	●

●	●	●	●	●

●	●

●

●	●	●	●	●

●

●	●

●	●	●

●	●

●

●	●	●

●	●	●	●	●	●	●	●	●

●	●	●	●

●	●

●	●	●	●

●	●	●

●

●	●●	●

●	●

●

●

●	● ●	●	●	●	●	●

●	●	●	●	●

●	●

●	●

●

●

●●	●	●

●

●	● ●

●

●	●	●

●

●	●	●	●	●	●

●	●	●

●	●

●	● ●	● ●	●

●	●	●

●	●	●

●	●	●	●	●	●	●	●	●	●	●●	●	●	●

●	●	●	●	●

●	●	●	●	●	●	●

●

●	●	●	●	●	●

●	● ●	●

●

●

●	●	●	●	●

●	●

●	●	●	●	●●	●	●

●

●	●

●	●	●

●	●	●	●	●	● ●

●	●	●	●	●	●	●	●●	●	●	● ●	●	●

●●	●	●

●	●	●	●	●	●	●	●	●	● ●	● ●

●	●

●

●

●

●

●	● ●

●	●

●	●	●	● ●	●	●

●	●

●	●	●	●	●	●

●

●

●	●

●	●

●	●

●	●	●

●	●

●

●	●	●	●

●	●

●	●

●

●

●	●

●	●

●

●	●	●	●

●	●	●	●	●●	●	●	●	●	●	●

●	●	●	●

●

●	●	●	●	●	●

●	●	●	●	●	●	●

●	●

●	●	●	●

●	●	●	●●	●	●●	●

●	●	●	●

●

●

●	●	●	●

●	●	●

●	●	●	●

●

●	●

●	●	●

●	●	●	●	●	●	●	●	●	●

●	●	●	●

●	●	●	●	●	●	●	●	●	●	●	●	●	●

●

●

●	●	●	●	●	●	●	●	●	●	●	●	●

●	●	●	●	●	●

●	●	●	●

●

●

●

●	●

●	●	●	●	●	●

●	●	●	●	●	●	●

●	●	●	●●	●

●	●	●

●

●

●	●

●

●

●	●	●

●

●

●	●	●	●	●	●

●	●

●	●	●

●

●	●	●

●	●

●●	●

●

●

●	●	●	●●	●

●	●	●	●	●	●

●	●	●

●	●	●	● ●	●

●	●	●	●

●	●

●	●	● ●

●	●	●	●	●	●

●

●	●	●	●	●	●	●	●

● ●●

●

●	●	●	●	●

●	●

●	●	●

●	●

●	●	●	●	●	●	●	●	●	●

●	●	●	●

●	●

●

●	●	●	●	●

●

●	●

●

●	●	●	●	●

●	●	● ●	●	●	●	●	●	●

●

●

●

●	●	●

●	●	●	●	●

●	●	●	●	●	●	●	●	●	●	●	●

●	●	●

●	●	●	●	● ●	●

●

●

●	●	●	●	●	●

●

●	●	●	●	●	● ●

●	●	●	●	●	●

●	●	●

●	●

●	●

●	●	●	●	●	●	●	●	●	●	●

●

●	●

●

●	●●	●	●	●	●

●

●	●	●	●	●

●

●

●	●

●

●	●	●	●	●	●

●

●

●

●	● ●	●

●	●	● ●	●	●	●	●	●	●	●

●

●

●	●

●	●	●	●

●

●	●	●

●

●	●	●	●	●	●

●	●	●	●

●	●●	●

●

●

●

●

●

●●	●	●	●

●

●	●	●	●

●	●	● ●	●	●

●

●	●

●	●

●

●●	●	●	●	●	●	●	●	●	●

●	●	●●	●	●	●	●

●	●	●

●

●	●	●	● ●

●	●	●

●	●

●

●	●

●	● ●●

●	●	●	●

●	●	●

●	●	●

●

●	● ●

●	●	●	●	●	●	●	●

●	●

●	●	●	●

●

●	●	●	●	●	●

●

●

●	●

● ●	●

●	●

●

●

●	●	●

●	●	●	●

●	●	●	●	●	●	●

●

●	●	●

●	●

●	●	●	●	●	●	●	●	●	●	●

●	●	●

●

●	●	●

●	●	●

●

●	●	●

●	●	●

●

●	●	●

●	●

●	●	●	●	●	●	●	●

●

●	● ●	●	●

●	●

●

●

●	●	●	●	●

●	●	●	●

●

●	●	● ●	●	●	●	●

●	●	●	●	●	●	●

●	●	●	●	●	●	●	●	●	●	●	●

●	●	●	●	●	●	●	●

●

●	● ●

●

●

●	●

●	●

●

●	●	●	●

●	●	●

●	●	●	●	●	●	●

●	●	●	●	●	●	●	●	●	●	●

●●	●	●

●

●	●	●

●	●	●

●	●	●

●

●	● ●	●	●	●	●

●

●

●	●

●

●	●

●	●

●

●	●	●

●	●

●	●	●	●	●	●

●	●	●

●

●	●	●

●

●●	●	●	●	●	●

● ●	●●

●

●	●

●	●	●	●

●

●	●

●	●	●

●	●

●

●

●

●	●

●	●	●	●	●	●	●	●

●

●	●

●	●	●	●	●	●	●	●	●

●	●	●	●	●	●	●	●	●●	●	●

●	●

●	●

●	●	●	●

●	●

●

●	●

● ●

●	●	●	●

●

●	●	●	●	●	●	●

●

●	●	●

●	●

●	●

●

●	●

●

●

●

●	●

●

●

●	●

●	●

●	●

●

●	●	●

●	●	●

●	●	●

●	●

●	●

●	●

●	● ●	●

●	●

●

●

●

●	●	●

●	●

●	●	●	●●	●	●

●	●	●	●

●	●

●	●	●

●	●	●	●

●

●	●

●	● ●	●	●

●	●

●	●	●	●	●	●	●	●

●	●	●	●	●

●

●	●

●	●

●	●	●

●	●

●	●	●

●	●

●	●	●

●

●	●

●	●

●

●

●

●

●	●

●	●	●	●	●

●	●

●	●	●

●	●	●	●	●

●	●

●

●	●	●	●	●

●	●	●	●	●

●	●	●	●	●	●	●

●	● ●	●	●

●

●

●	●

●

●

●	●

●	●	●

●

●	● ●	●	●

●	●

●

●

●	●

●

●	●	●	●	●	●

●	●	●

●	●

●

●	●	●	●	●

●	●	●	●	●

●	●

●	●	●

●

●	●	●

●

●	●	●	●	●	●

●	●

●	●	●	●

●	●

●	●

●

●

● ●

●	●	●	●

●	●	●

●

●	●	●

●	● ●	●	●

●

●

●	●	●

●	●	●	●	●

●	●	●	●	●

●	●	●	●

●	●	●	●

●	●

●

●

●

● ●	●

●

●

●

●	●	●

●	●	●	●

●

●	●

●

●	●

●	●

●

●

●

●

●	●	●	●	●	●

●

●

●	●

●

●	●

●	● ●	●	●	●	●	●	●	●	●	●●	●	●

●	●	●	●

●	●	●	●	●	●	●	●	●

●

●	●

●

●

●	●	●

●

●

●

●

●

●

●	●	●

● ●	●

●	●	●	●	● ●	●	●	●	●	●	●	●	●

●	●

●	●	●	●

●	● ●

●	●

●

●

●	●

●

●	●	●

●

●	●

●

●

●

●

● ●

●	●

●	●	●	●	●

●	●	●	●	●	●

●	●	●	●	●	●	●	●

●

●

● ●	●

●	●	●	●	●	●	●

●

●	●	●

●	●	●	●	●	●	●	●	●	●	●	●	●

●	●

●	●	●	●	●	●	●	●

●	●	●	●

●

●

●

●	●	●	●

●

● ●	●	●	●	●	●	●

●

●	●	●

●	●	●

●	●	●	●

●

●

●	●

●

●	●

●

●

●

●

●	●

●

●

●

●

●

●

●	●

●

●	●	●

●

●	●	●	●	●	●	●	●

●	●	●	●	●	●

●	●	●

●	●

●

●	●

●	●

●

●

●	●	●	●	●

●

●	●	● ●	●

●	●	●	●	●	●

●	●

●	●

●

●	●	●

●	●

●

●

●

●	●

●	●

●

●

●	●	●	●

●

●	●	●	●

●

●

●

●	●

●

●

●	●

●

●

● ●	●

●

●	●	●	●

●	●

●	●

●	●	●	●	●	●

● ●

●

●	●	●	●

●	●

●	●	●

●

●

●

●	●	●	●

●	●

●	●

●

●

●

●	●	●	●

●

●

●

●

●

●

●	●	●

●	●

●

●	●	●

●	●	●	●	●	●	●	●

●	●	●	●	●

●

●	●	●	●

●	●	●	●	●	●	●	●	●	●	●	●

●	●	●

●

●	●	●

●	●

●

●	●

●

●	●	●

●	●	● ●	●

●	●	●

●	●	●	●

●

●

●	●

●	●

●	●	●	●	●	●	●	●	●	●

●	●	●

●	●

●

●

●	●	●

●	●

●	●

●	●

●

●	●	●	●	●	●	●	●	●	●

●	●

●	●

● ●

●

●

●	●

●

●	●

●	●	●	●	●	●	●

●	●	●	●	●	●	●

●

●	●	●●	●

●	●	●

●

●

●	●

●	●	●	●

●	●	●	●

●	●	●	●

●

●

●	●	●	●

●

●	●	●	●	● ●	●

●	●	●	●

●	●	●

●

●

●	●	● ●

●	●	●

●	●	●

●

●	●	●

●	●	●	●

●	●	●	●	●	●	●

●

●

●	●

●	●

●

●

●

●

●	●	●

●	●	●

●	●

●

●	●

●	●

●	●

●	●

●

●	●

●

●	●

●	●

●

●

●	●	● ●

●

●

●	●	●

●

●

●	●

●	●

●

●	●

●	●

●

●

●

●

●

●	●	●	●

●	●	●

●

●	●	●	●	●

●

●

●

●	●

●	●

●	●	●	● ●	●

●

●	●

●	●	●

●	●

●

●	●

●

●	●

●●

●	●	●	●	●

●	●	●

●	●	●	●	●

●

●

●

●

●	●	●

●

●

●	●

●	●

●	●	●	●

●	●	●

●	●	●

●	●	●

●

●	●	●

●

●

●	●

●

●

●

●

●

●	●	●

●	●	●	●	●	●

●

●

●

●	●

●	●	●	●

●

● ●

●

●

●

●	●

●	●

●	●	●	●

●

●	●	●	●	●	●	●●	●	●	●	●

●

●	●

●

●

●

●	●

●	●	●

●	●

●	●

●	●

● ●

●

●

●	●	●	●

●

●

●

●	●

●	●

●	●	●

●	●	●

●

●

●

●

●

●

●	●

●

●

●	●

●	●	●	●	●	●

●

●

●

●

●

●

●	●	●	●	●	●

●

●	●	●	●	●	●	●	●	●	●	●

●	●	●	●

●

●

●

●	●

●	●

●	●

●	●	●	●

●

●	●	●	●	●

●	●

●	●	●	●	●	●	●	●	●	●

●

●	●

●	●	●

●	●

●	●

●	●	●	●	●

●

●●	●	●

●	●

●

●	●

●

●

●

●

●

●	●	●

●

●	●	● ●	●	●

●

●

●	●

●

●	●

●

●

●

●

●

●

●	●

●	●

●

●	●	●	●

●

●	●

●

●

●

●

●

●

●	●	●

●	●	●

●

●

●

●	●

●

●	●

●

●	●

●	●	●

●	●	●

●

●

●	●	●

●

●

●

●

●	●

●	●

●	●

●	●	●

●

●

●

●

●

●	●

●

●

●

●

●

●

●

●	●	●

●	●	●

●

●	●

●

●	●

●	●	●

●	●	●	●	●	●	●	●	●	●

●

●

●	●

●

●	●	●

●	●

●

●

●

●	●

●

●

●	●

●

●	●

●

●

●

●

●	●	●

●

●

●

●

●	●

● ●	●

●	●	●

●	●

●

●

●

●●

●	●	●

●

●	●	●

●

●

●

●

●	●

●

●

●

●	●

●	●

●

●	● ●	●	●

●	●

●	●

●

●

●	●	●

●	●

●

●	●

●

●

●

●

●

●	●	●

●

●

●

●	●

●	●

●

●	●

●

●	●	●	●	●

●

●

●

●

●	●

●

●	●	●

●	●

●

●	●

●●

●

●

●	●	●	●	●	●	●	●●	●

●	●

●

●

●	●

●	●

●

●

●	●

●

●	●

●	●

●	●

●

●	●

●

●

●	●	●

●

●

●

●

●

●	●

●	●

●

●

●	●	●

●

●

●	●	●

●

●	●

●

●

●

●

●

●	● ●	●

●	●	●

●

●

●

●

●	●

●

●	●	●

●	●	●

●

●	●	●

●

●

●

●	●

●

●

●

●	●

●

●	●

●	●	●	●

●	●

●	●

●

●

●	●	●	●

●	●

●

●	●

●

●

●	●

●	●	●

●

●

●

●

●

●

●

●	●	●

●

●	●	●	●

●	●

●

●

●

●	●

●

●

●

●

●

●

●

●

●

●	●

●	●	●

●

●

●

●

●

●

●

●	●	●

●

●

●	●

●

●

●

●	●

●

●

●

●

●	●	●

●

●

●

●

●

●	●	●

●

●	●

●	●

●	●

●

●

●	●	●	●

●

●	●	●

●

●

●

●	●	●	●

●	●

●

●

●

●

●

●

●

●

●	●

●

●

●

●

●

●

●

●

●

●

●

●

●

●	●

●

●

●

●

●

●

●

●	●

●

●

●

●

●

●

●

●

●

●	●

●

●

●	●

●

●

●

●

●

●

●

●

●

●	●

●

●

●

●	●	●

●

●

●

●

●

●

●	●

●

●	●

●

●	●

●

●	●

●	●	●

●

●

●

●	●	●

●

●

●

●	●

●	●	●	●	●

●

●	●	●

●	●

●

●	●

●

●

●

●	●	●	●	●	●	●	●

●

●	●	●

● ●	●

●	●

●

●

●

●	●	●	●	●

●

●

●	●	●

●

●	●

●

●	●

●	●	●

●	●	●	●	●	●	●	●	●	●	●

●	●	●	●	●

●	●	●

●	●	●	●

●	●	●

●

●

●	●	●	●	●	●	●	●	●	●

●

●	●	●

●●

●	●

●	●

●	●

● ●	●

●	●	●

●

●	●	●

●	●

●	●	●	●	●

●	●	●	●	●	●	●	●	●

●	●	●

●	●

●

●	●

●	●	●	●	●

●

●	●	●	●	●	●●	●●	●

●	●	●	●	●	●	●	●	●	●	●

●	●	●	●	●	●●	●

●	●	●	●	●

●

● ●

●	●

●

●

●

●	●	● ●	●	●

●

●	●

●	●	●	●

●	●

●

●	●	●

●	●	●	●	●	●

●	●	●	●	●

●

●	●	●	●	●	●	●	●

●	●	●

●	● ●	●	●	●

●

●	●● ●	●	●●	●	●

●

●	●	●	●	●	●

●	●	●●	●	●	●	●

●	●	●	●	●	●	●	●	●	●

● ●

●

●

●	●

●

●	●	●	●

●

●	●	●	●	●	●	●	●	●	●

●●	●	●	●	●	●

●	●	●	●	●	●	●

●	●	●

●

●	●	●

●

●	●	●	●	●

●	●

●	●	●	●	●	●	●

●	●	●	●	●	●	●	●	●

●

●	●	●

●

●	●	●

●

●

●	●	●	●

●	●

●	●	● ●

●

●

●	●

● ●	●●	●	●

●	●	●	●	●	●	●	●	●	●	●	●

●	●

●	●	●	●	●	●	●

●	●	●

●	●	●

●	●	●	●	●

●	●	●	●

●

●●	●	●

●

●	●

●	●

●

●	●	●

●	●	●	●	●	●	●	●	●	●	●	●	●●	●	●	●

●	●	●	●

●	●	●	●	●	●	●	●	●	●	●

●

●

●	●

●

●	●	●	●	●	●	●

●	●	●

●	●	●

●	●

●	●	●	●

●

●	●

●	●

●

●	●

●

●

●

●

●	●

●	●	●

●	● ●	●	●	●	●●	●	●	●	●	●	●

●	●

●	●	●

●	●	●	●	●	●

●	●	●	●	●	●	●	●

●	●

●	●	●	●	●	●

●	●	●

●

●	●

●

●	●

●	●	●

●

●	●

●	●	●	●	● ●	●	●

●	●	●	●	●	●

●	●	●	●

●	●	●

●	●

●

●	●	●	●

●

●

●

●	●

●	●

●	●	●	●

●	●	●	●	●

●

●	●	●	●	●	●	●	●	●	●	●

●	●

●	●

●	●	●	●	●	●	●	●	●	●

●

●	●

●

●	●	●	●

●	●	●	●

●

●	●	●	●

●

●

●●

●

●

●	●	●	●	●	●	●	●	●	●	●	●	●	●	●	●

●	●	● ●	●	●	●	●	●	●	●	●

●	●

●

●	●	●

●	●	●	●

●	●	●

●

●

●	●●	●	●	●	●	●	●

●

●	●

●	●

●

●

●	●	●	● ●

●	●	●	●	●	●	●	●

●	●	● ●	●	●	●	●	●	●	●	●	●	●	●	●

●	●	●	●	●

●	●	●	●	●	●

●	●	●	●	●	●	●●

●	●	●	●	●	● ●	●	●	●	● ●	●

●	●	●

●

●

●	●	●	●	●	●

●	● ●	●	●	●	●	●	●	●	●

●	●	●

●	● ●	●	●

●	●

●

●	●	●	●	●	●	● ● ●

●	●	●	●

●

●	●	●	●	●	●	●	●	●	●

●

●

●

●

●	●	●

●	●	●	●	●	●

●

●●

●	●	●	●

●	●	●

●	●	●	●

●	●	●	●

●	●	●	●	●

●	●	●

●	●	●	●	●

●	●	●	●

●

●	●	●	●	●

●	●

●	●	●	●

●	●

●	●	●	●	●	●	●●	●	●	●	●

●

●	●	●

●	●	●

●

●	●	● ●	●	●	●	●	●	●	●	●

●	●	●	●	●	●	●	●	●	●	●	●	●	●	●	●	●	●	●

●

●	●

●

●	●	●	●	●

●	●	●

●	●

●	●	●	●

●

●	●

●	●

●	●	●

●	●	●

●

●	●	●	●	●	●

●	●	●

●

●

● ●	●	●	●	●	●	●	●	●	●	●

●	●	●	●

●	●	●

●	●

●	●	●

●

●	●	●

●	●	●	● ●	●	●

●	●

●

●

●	●	●	●

●

●

● ●	●	●

●	●	●	●	●	●	● ●

●●	●	●	●	●	●	●	●●	●	●	●	●	●

●

●●	●	●	●	●	●	●	●	●	●	●	●	●	●	●	●	●	●	●	●

●	●	●	●	●●

●	●	●	●	●	●	●	●	●	●	●	●	●	●	●	● ●	●	●	●	●	●	●	●	●	● ●	●	●	●	●

●

●

●	●	●	●

●

●	●	●	●	●	●	●

●	●	●	●	●

●	●	●	●	●

●	●	●	●	●	●	●	●	●	●

●	●	●	●	●	●

●	●	●	●	●

●

●	●	●

●	●●	●

●

●	●

●	●	●	●

●	●	●

●

●	●	●

●

●

●	●	●	●	●	●	●

●	●

●	●	●	●	●	●

●	●	●	● ●

●	●

●	●	●	●	●	●

●	●	●

●	●

●

●	●	●	●	●	●	●	●	●

●●	●	●	●	●	●	●	● ● ●	●	●

●

●	●	●	●	●	●

●

●

●	●

●	●	●	●	●	●	●	●

●

●

●

●

●	●	●

●	●	●	●

●	●

●	●	●	●● ●	●	●	●	●●	●	●	●	●

●	●

●

● ●	●	●	●	●	●

●	●	●	●	●	●

●	●	●	●

●	●	●	●	●	●	●	●	●	●	●

●	●

●

●	●

●

●

●	●	●	●

●

●	●

●

●

●	●

●	●●	●	●	●	●	●	●	●	●

●	●	●	●	●	●	●	●	●	●

●

●	●

●	●	● ●	●	●	●

●	●	●	●	● ●	●	●	●	●	●

●

●	●	●

●	●	●

●	●	● ●	●	●	●	●	●	●	●	●	●

●	●

●

●

●	●	●	●

●	●	●	●

●

● ● ● ● ●●

●

●

●	●	●	●

●	●

●

●	●	●

●	●

●	●	●	● ●	●	●	●	●	●	●	●

●	●	●

●	●

● ●	●	●	●

●	●

●	●	●	●	●	●

●●	●	●

●	●	●	●	●●	●	●	●	●	●	●	●	●	●	●	●	●	●

●	●	●	●●	●	●	● ●	●	●	●	●	●	●	●	●

●

●

●	●	●	●

●

●	●	●	●	● ●	●

●	●	●	● ●

●	●	●	●	●	●

●	●	●	●	●

●	●	●	●

●	●

●	●	●	●	●	●	●	●	●

●	●	●	●

●	●

●

●	●	●

●	●	●	●	●	●●	●	●

●	●	●	●	●	●	●	●	●	●	●	●

●	●

●

●	●	●	●	●	●	●

●	●	●	●

●	●

●	●	●	●	●	●	●	●	●

●

●

●	●	●	●

●	●	●	●	●	●

●	●	●

●	●	●	●

●	●

●	●

●

●	●

●	●	●	●	●	●

●	●	●	●	●	●	●	●	●	●	●	●	●	●	●	● ● ●	●

● ●	● ●	●	●●	●	●	●	●	●

●	●

●	●	●	●	●

●

●

●	●	●	●	●	●	● ●	●

●	●	●	●	●	●	●	●	●	●	●	●

●	●	●	●	●

●	●

●	●

●	●

●	●

●	●	●

●	●	●	● ●	●	●	●

●	●	●

●

●	●	●	●	●	●	●

●	● ●	●	●	●	●	●	●	●

●	●	●	●

●	●	●	●	●	●	●

●	●	●	●	●	●	●

●	●	●

●

●	●	●

●	●

●	●	●	●	●

●	●	●	●	●

●

●	●

●

●	●	●	●	●●	●	●	●	●

●	●	●

●	●	●	●	●	●

●	●	●	●	●

●

●	●	●	●	●

●●	●	●	●	●	●	●	●

●	●	●	●	●

● ●

●	●	●

●	●	●	●	●	●	●	●	●

●	●	●	●	●	●

●

●	●	● ●

●	●	●	●	●

●	● ●	●	●	●	● ●	●

●	●	●	●	●

● ● ● ● ● ● ● ● ● ● ●●

●	●	●	●	●	●	●	●	●	●	●

●	●	●

●

●	●

●	●	●	●	●	●

●	●	●	●

●	●	●	●

●	●

●

●	●	● ●	●

●	●	●

●

●	●	●	●	●	●	●

●

●	●	●	●	●	●	●	●

●	●	●	●	●

●

●

●

●	●

●	●

●	●

●	●

●

●

●

●	●

●	●	●

●

●	●

●	●	●	●	●	●

●	●	●	●	●

●	●●	●	●	●	●	●	●	●	●

●	●	●	●

●	●	●

●

●

●	●

●	●

●

●	●	●

●

●	●	●

●

●	●	●	●	●	●	●●	●	●	●

●	●	●	●

●	●	●

●

●●	●	●	●	●

●	●	●	●	●

●	●

●

●	●	●	●

●

●	●	●	●	● ●

●	●	●	●

●	●	●	●	●	●

●	●	●

●	●	●	●	●	●	●	●	●

●●	●	●	●

●	●	●	●	●	●

●	●

●	●	●	●	●	●

●	●	●	●	●

●	●	● ●	●	●

●	●	●	●	●	●	●	●

●

●	●	●

●

●

●	●	●	●

●	●

●	●	●	● ●	●

●	●	●

●	●	●	●	●	●	●	●	●	●	●	●	●	●

●

●

●

●	●	●	●	●	●	●	●	●	●	●

●	●	●	●

●

●	●	● ●	●	●	●	●	●	●	●

●	●

●

●	●	●	●	●	●	●	●	●	●

●

●	●

●	●	●

●	●

●	●	●

●	●	●	●	●	●	● ●	●	●	●	●	●	●	●	●	●	●

●	●	●	●	●	●	●

●	●

●

●	●

●	●

●	●

●	●	●	●	●	●	●

●	●	●	●	●	●	●

●	●	●	●	●	●	●	●	●	●	●	●	●	●

●	●	●	●	●	● ●●	●	●

●	●	●

●	●	●	●	●

●

●

●

●	●	●

●

●	●	●

●	●	●

●	●

●

●

●	●	●

●

●	●

●	●	●	●

●

●	●

●

●	●	●

●	●	● ●	●

●	●	●	●	●	●

●	●	●	●	●	●

●	●	●	●

●	●	●

●	●

●	●	●	●	●	●

●	●

●	●	●

●

●	●

●	●	●	●●	●

●	●	●

●	●

●	●	●

●	●

●

●

●

●	● ●	●	●	●	●	●	●	●

●

●	●	●

●

●

●	●	●	●

●	●	●	●●	●	●	●	●

●	●	●	●	●	●	●	●

●	●	●	●	●	●	●	●

●	●

●	●	●

●

●

●	●

●	●

●	●	●	●	●	●	●●	●	●

●	●	●

●	●	●

●	●	●	●	●

●	●

●	●	●

●	● ●	●	●	●

●

●	●	●	●	●	●	● ●	●

●●

●	●	●	●	●	●	●

●	●	●

●	●	●●	●	●●	●	●

●

●

●	●

●	●	●	●	●

●	●	●	●	●

●	●	●	●

●	●

●	●	●	●	●	●

●

●	●	● ●	●

●	●	●

●

●	●	●	●	●

●●

●	●	●

●	●

●

●	●

●	●	●	●

●	●	●

●	●

●	●	●	●	●	●

●

●	●	●	●

●

●	●	●	●	● ●	●	●	●	●	●	●	●

●	●	● ●	●

●	●

●	●	●	●	●

●	●

●

●	●

●	●	●

●	●	●

●	●	●

●	●	●	●	●	●

●	●

●	●	●	● ●	●

●

●	●	●	●	●	●

●	●	●	●	●	●	●

●	●	●	●	●	●	●

●	●

●	●

●	●	●	●	●	●

●

●	●	●	●

●	●

●	●	●	●	●	●	●

●

●	●	●	● ● ●	●

●	●	●	●	●

●	●	●	●	●	●	●	●	●	● ● ●	●	●

●	●	●	●	● ●

●

●

● ●	●

●

●

●	●	●	●	●	●	●	●	●	●	●

●

●	●

●	●	●

●	●	●

●	●

●	●	●	●	●	●	●	●

●	●	● ●	●

●

●

●	●●	●	●	●	●	●	●●	●	●	●	●	●	●

●	●	●	●

●	●	●	●	●	●	●	●	●	●	●	●	●

●	●	●

●

●	●	●

●	●	●

●	●	●	●

●	●	●	●

●

●	●	●

●

●	●

●

●	●

●	●	●	●	●

●	●	●	●	●	●

●	●	●	●	●

●	●	●	●	●

●	●

●

●

●	●

●	●

●

●

●

●	●	●	●

●

●	●	●	●	●	●	●	●	●	●	●	●

●

●	●	●	●

●	●	●

● ●

●	●	●	●	●	●	●	●	●

●

●	●	●	●	●	●	●	●	●

●	●	●

●

●

●

●	●	●	●	●

●

●

●	●

●

●	●

●	●	●	●

●	●	●	●	●	●	●	●	●	●	●

●●	●	●

●	●

●	●

●

●	●

●

●	●	●

●	●	●

●	●	●

●	●	●	●

●	●

●	●	●	● ●	●	●	●

●	●

●

●

●	●

●	●	●	●

●

●	●	●

●

●	●

●	●

●	●	●

●	●	●	●	●	●	●	●	●	●	●

●●	●

●	●	●	●

●	●	●	●

●	●	●	●	●

●	● ●

●	●	●	●

●	●	●	●	●	●	●	●	●

●	●	●	●	●	●

●	●

●	●	●

●

●

● ●	●	●

●	●	●	●

●	●	●	●	●	●

●

●	●	●	●

●

●	●	●

●	●	●	●	●	●	●	●	●	●	●	●	●	●	●	●	●	●	●

●	●

●

●

●	●	●

●

●	●	●	●

●	●	●	●	●	●	●●	●	●

●	●

●	●

●	●	●	●

●	●	●

●	●

●

●	●

●	●	●	●	●	●	●	●

●	●

●

●	●

●	●

●	●

●

●

●

●

● ●	●	●	●	●	●	●

●	●	●	●

●	●

●	●

●	●	●	●

●

●

●

●

●	●	●

●	●

●	●

●	● ●

●	●

●

●	●	●	●	●	●	●

●	●	●	●

●	●	●

●	●	●	● ●	●

●	●	●

●

●	●	●	●	●	●

●●	●

●

●	●

●	●	●	●	●	●

●	●	●	●	●

●

●	●	● ●	●

●

●	●	●	●	●	●

●

●	●	●●	●	●	●

●

●

●	●

●	●	●	●	●	●	●	●

●	●	●

●	●	●	●	●	●

●	●

●	●	●	●	●

●	●

●

●

●

●	●	●	●	●

●	●	●	●

●	●	●	●	●	●

●	●	●	●	●

●

●	●	●	●

●	●	●	●

●	●

●

●	●

●

●	●	●	●	●

●	●

●	●	●	●	●	●	●	●	●	●	●	●

●

●

●	●	●	●	●	●	●

●	●	● ●	●	●	●

●

●	●	●	●	●	●	●	●

●●	●

● ●	●	●	● ●

●	●	●

●	●	●

●	●	●	●	●	●	●	●

●	●	●	●

●	●

●	●	●	●	●	●

●	●	●	●	●	●	●

●	●	●	●	●

●	●	●	●

●	●	● ●	●	●	●	●●	●	●	●	● ●	●

●	●	●

●	●	●

●	●

●	●	●	●

●	●

●	●

●	●

●

●	●

●	●	●

●	●

●	●	●	●●	●

●

● ●

●

●

●	●	●	●

●

●	●	●

●	●

●

●

●	●	●	●

●

●	●	● ●	●

●	●

●

●

●	●	●	●	●

●	●	●	●

●	●	●	●	●	●

●	●	●	●	●	●	●	●

●	●	●	●	●	●	●

●

●	●	●	●	●

● ●	●

●	●

●

●	●

●

●

●

●	●	●	●	●

●	●	●

●	●	●

●	●	●

●	●	●

●

●

●

●	●

● ●	●

●	●

●	●	●	●	●	●

●	●	●	●	●

●	●	●	●	●	●

●	●	●	●	●	●	●

●	●	●

●	●

●	●

●

●	●	●	●	●	●	●

●	●

●

●

●	●

● ●

●	●	●	●	●	●	●	●	●	●	●	●	●	●	●	●	●	●	●	●

●

●	●

●	●

●

●	●	●	●

●	●	●

●	●	●	●	●

●	●	●	●	●

●	●	●

●

●	●

●	●	●	●

●

●

●	●

●

●

●

●	●	●	●	●	●

●	●	●	●

●	●

●

●	●

●

●

●	●	●

●	●

●	●	●

●	●	●	●

●	●

●	●	●	●	●	●

●	●	●

●

●	●

●

●	●

●	●

●

●	●	●	●

●	●	●

●	●	●	●	●

●	● ●	●	●	●	●	●

●

●	●	●	●

●	●

●

●	●	●

●	●

●

●	●	●	●

●	●

●

●	●

●	●	●

●

●	●

●	●

●	●	●	●

●

●	●

●

●

●

●	●

●

●	●	●	●	●

●	●

●

●	●	●

●	●

●

●	●	●

●	●	●	●	●	●	●	●

●	●

●	●	●

●

●	●

●	● ●

●	●	●	●

●

●

● ●	●

●	●

●

● ●	●	●	●	●

●	●	●	●

●	●	●	●	●

●	●

●

●	●

●	●

●

●

●

●

●

●	●

●

●

●

●	●	●

●	●	●	●

●	●

●

●

●	●

●	●

●	●

●	●	●	●

●

●

●

●	●

● ●	●	●	●

●	●

●	●	●	●

●

●	●

●	●

●

●	●	●	●

●	●	●	●	●

●

●

●●	●	●

●	●	●	●

●

●	●

●

●	●

●

●

●	●

●

●

●	●	●

●	●

●	●	●	●

●	●

●	●

●

●	●	●

●	●	●	●	●	●

●

●	●

●	●	●

●

●

●	●

●

●	●	●	●

●	●	●	●

●	●	●

●	●

●	●	●

●	●

●

●	●	●	●

●	●	●	●	●	●●	●	●	●

●	●	●	●

●	●	●

●

●	●	●	●	●	●	●	●	●

●	●	●

●	●	●	●

●

●	●	●	● ●	●	●	●	●	●

●	●

●	●	●	●

●	●

●	●	●	●	●	●	●	●	●	●

●	●	●	●	●	●

●	●	●	●	●	●

●

●	●

●

●

●

●	●

●	●	●	●	●	●

●	●	●

●

●	●

●	●	●

●

●	●

●

●	●	●	●	●	●	●

●	●	●

●

●	●	●

●

●	●	●	●	● ●

●	●	●

●

●

●	●	●

●	●	●	●	●

●

●

●

●

●	●	●	●

●

●

●

●	●

●

●	●	●	●	●	●	●	●	●	●	●	●	●	●	●	●

●	●

●	●

●

●

●

●	●	●	●	●

●

●

●

●

●

●	●	●	●

●

●	●	●

●	●

●

●

●	●

●

●	●●	●

●

●	●	●	●	●

●

●

●

●

●

●

●

●

●	●

●

●

●

●	●	●	●	●

●

●	●	●	●	●	●	●

●

●

●	●

●

●●

●	●	●

●

●	●

●

●	●

●	●	●

●

●

●

●

●	●

●

●

●

●

●

●	●	●

●

●	●

●

●	●	●

●

●

●

●

●

●	●	●

●	●	●

●	●

●	●

●	●	●

●

●	●

●

●

●

●	●	● ●	●

●

●

●

●

●	●	●	●	●

●	●

●

●	●

●

●	●

●

●

●	●

●	●

●	●

●	●

●

● ●

●	●	●

●	●

●

●	●

●	●	●	●	●	●	●

●

●

●

● ●	●	●	●

●

●	●

●

●

●

●	●

●	●	●

●

●

●

●

●

●	●

●

●	●	●

●

●

●	●

●

●●	● ●

●

●	●

●	●

●

●	●

●

●

●

●

●	●

●	●	●

●	●

●	●	●

●

●

●	●	●

●

●	●	●

●	●

●

●

●

●

●

●	●

●

●	●

●	●	●

●	●	●	●	●	●

●

●

●	●

●

●	●

●

●	●	●	●

●	●

●

●

●

●

●

●	●

●	●

●

●

●

●	●	●	●

●

●

●

●	●

●

●	●

●

●	●

●

●

●

●

●	●	● ●

●	●	●

●

●

●

●

●

●

●	●

●

●

●

●	●

●

●

●

●	●

●	●

●

●

●

●	●

●

●	●

●	●

●

●

●	●

●

●

●

●

●

●

●

●	●

●

●

●	●

●

●	●

●

●	●

●	●	●	●

●

●

●

●

●

●	●	●	●

●	●	●	●

●

●	●	●

●

●

●

●

●	●

●	●

●	●

●

●

●

●	●	●	●

●

●

●

●

●

●

●

●

●	●

●	●

●

●

●

●

●

●

●

●	●	●

●

●

●

●

●

●	●	●	●	●	●	●

●	●

●

●

●

●	●	●

●

0.75	0.75

0.50	0.50

Relative value

Relative value

0.25	0.25

0.00

0.00	0.25	0.50	0.75	1.00

Relative methylation level (CHG)

0.00

0.00	0.25	0.50	0.75	1.00

Relative methylation level (CHH)

Recombination rate

Sequence parental divergence

Total SVs load

Genetic effects

Methylation level: CpG	Methylation level: CHG	Methylation level: CHH

Fig. S4: Relative value of genomic features in the 1 Mbp genomic windows in which the explanatory feature was found significantly correlated with the variation in the recombination rate among the 45 double round-robin (DRR) populations. The significance of the correlation was determined by a stepwise regression procedure. The studied genomic features included the recombination rate (lightblue), sequence divergence among parental inbred lines (orange), total structural variants (SV) load (yellow), genetic effects (green), and methylation level in the sequence contexts CpG (red), CHG (maroon), and CHH (purple).The explanatory features are (A) sequence parental divergence, (B) total SV load, (C) genetic effects, (D, E, and F) methylation level in CpG, CHG, and CHH sequence contexts, respectively. The dots correspond to the relative value of the recombination rate.

Insertions Deletions Inversions Duplications

Parental sequence divergence

Genetic effects Methylation level: CpG Methylation level: CHG Methylation level: CHH

Insertions Deletions Inversions Duplications

Parental sequence divergence

Genetic effects Methylation level: CpG Methylation level: CHG Methylation level: CHH

Insertions Deletions Inversions Duplications

Parental sequence divergence

Genetic effects Methylation level: CpG Methylation level: CHG Methylation level: CHH

Insertions Deletions Inversions Duplications

Parental sequence divergence

Genetic effects Methylation level: CpG Methylation level: CHG Methylation level: CHH

Insertions Deletions Inversions Duplications

Parental sequence divergence

Genetic effects Methylation level: CpG Methylation level: CHG Methylation level: CHH

Insertions Deletions Inversions Duplications

Parental sequence divergence

Genetic effects Methylation level: CpG Methylation level: CHG Methylation level: CHH

Insertions Deletions Inversions Duplications

Parental sequence divergence

Genetic effects Methylation level: CpG Methylation level: CHG Methylation level: CHH

0

200

7H

Physical position (Mbp)

400

600

1.0

2H

3H

0.5

4H

0.0

−0.5

−1.0

5H

6H

1H

Fig. S5: Summary genomic features explaining the variation in the recombination rate among the 45 double round-robin (DRR) populations in 1 Mbp windows across the seven barley chromosomes. The significance of the correlation was determined by a stepwise regression procedure. The standardized regression coefficients are indicated by a color gradient from -1 (purple) to 1 (red). The studied genomic features included sequence divergence among parental inbred lines, genetic effects, span fraction of structural variants (SV) -such as insertions, deletions, inversions, and duplications-, and methylation level in the sequence contexts CpG, CHG, and CHH. The vertical solid line indicates the position of the centromere in the reference genome, and the vertical dashed lines indicate the pericentromeric region calculated across the 45 DRR populations.

HvDRR13	HvDRR27

38251

(9.0%)

92226

(21.6%)

51578

(12.1%)

83379

(19.6%)

256

(0.1%)

115480

(27.1%)

45238

(10.6%)

HvDRR28

Fig. S6: Venn diagram for the number of polymorphic single nucleotide polymorphism (SNP) markers detected by RNAseq in the analyzed double round-robin (DRR) populations HvDRR13, HvDRR27, and HvDRR28.

2019S−FA−02889−0000

2019S−FA−02896−0000

2019S−FA−02944−0000

2019S−FA−02951−0000

2019S−FA−02901−0000

2019S−FA−02915−0000

2019S−FA−02899−0000

2019S−FA−02950−0000

2019S−FA−02933−0000

2019S−FA−02912−0000

2019S−FA−02937−0000

2019S−FA−02946−0000

2019S−FA−02925−0000

2019S−FA−02917−0000

2019S−FA−02895−0000

2019S−FA−02907−0000

2019S−FA−02941−0000

2019S−FA−02914−0000

2019S−FA−02918−0000

2019S−FA−02908−0000

2019S−FA−02947−0000

2019S−FA−02886−0000

2019S−FA−02919−0000

2019S−FA−02923−0000

2019S−FA−02939−0000

2019S−FA−02880−0000

2019S−FA−02904−0000

2019S−FA−02898−0000

2019S−FA−02887−0000

2019S−FA−02948−0000

2019S−FA−02891−0000

2019S−FA−02930−0000

2019S−FA−02924−0000

2019S−FA−02879−0000

2019S−FA−02934−0000

2019S−FA−02926−0000

2019S−FA−02888−0000

2019S−FA−02949−0000

2019S−FA−02906−0000

2019S−FA−02942−0000

2019S−FA−02920−0000

2019S−FA−02877−0000

2019S−FA−02905−0000

2019S−FA−02883−0000

2019S−FA−02945−0000

2019S−FA−02878−0000

2019S−FA−02921−0000

2019S−FA−02932−0000

2019S−FA−02885−0000

2019S−FA−02881−0000

2019S−FA−02938−0000

2019S−FA−02890−0000

2019S−FA−02913−0000

2019S−FA−02935−0000

2019S−FA−02910−0000

2019S−FA−02916−0000

2019S−FA−02911−0000

2019S−FA−02903−0000

2018S−FA−02189−0000

2019S−FA−02893−0000

2019S−FA−02884−0000

2019S−FA−02892−0000

2019S−FA−02897−0000

0

100	200

300

Physical position (Mbp)

400

500

Chromosome 6H

Fig. S7: Stacked graphical genotypes of the recombinant inbred lines (RILs) for chromosome 6H in the HvDRR13 population. Different colors indicate parental allelic blocks inherited from different parents. The RILs are ordered according to their hierarchical complete clustering based on Euclidean distances of recombination rates for this chromosome. The vertical solid line in the background indicates the position of the centromere in the Morex v3 reference genome and the vertical dashed lines indicate the pericentromeric region calculated across the 45 DRR populations.

CO count

30

40

50

60

HvDRR13	HvDRR27	HvDRR28

●

●

20

Population

Fig. S8: Box-plot of the number of counted genome-wide crossovers (CO) with >3 Mbp marker blocks in the analyzed double round-robin (DRR) populations HvDRR13, HvDRR27, and Hv- DRR28.

HvDRR13	HvDRR27	HvDRR28

1.00

●

●●

●

●

●

●

●

● ●

● ●

● ●

●●

●

●

●

●

●● ● ● ●● ●●

●

●

● ● ●● ● ●

●●

●

● ●● ●● ●●

●

●

●

●●

●

●

●

●●

●

●

● ● ●

●

● ●

● ●

●

●

●

●

●

● ●●

●

● ●

● ●● ● ●

● ●

●

● ● ●

●

● ●

●

●

●

●

● ●●

●● ●

●

● ● ●

●

●●

●

●

●● ●● ●

●

● ● ●●● ●

●

0.75

0.50

1H

0.25

0.00

1.00

●

●

●

●

●

●

● ●

●

● ●

●

●

●

● ● ●

● ●

●

●

●

● ●●

●

● ●● ●

● ●

●●

●● ●●

●●●●● ● ●

●

●

● ●

●

●

● ● ● ●

● ● ●

●

● ● ● ● ●●● ●●

●

●

●

●

●

●

●

●

●

● ●

●

● ●

●

●●

●

●

●

●

● ●

●●

●

●

●

● ●

●●●●●● ●●●

●● ● ●

●

●

●

●

●●

● ● ●● ●

●

● ● ●

● ● ●●● ●

● ●● ● ●

●

●

●

●	●

●	●

●

●

●

● ●

●●

●

●

●

●

●

● ● ●

● ●●●● ●

●

●

●● ● ●●

●

●

● ● ●

●●●●● ●	● ● ●

●●●●●

●

●●

●

● ●

● ● ● ●● ● ● ●● ●●

●●	● ●● ●●

●●●●

0.75

0.50

2H

0.25

0.00

1.00

●

●

●

●

●

●

●

●

●

●

●

●

●

●

● ●

●

●

●

●

●

●

●●

●

●

● ● ● ● ●

●

●

● ●

●

●

●

● ●

● ●

● ● ●

●

●

● ●●●

● ● ●●

● ●●

●

●● ● ●●●●● ●●

●●

●

●

●

●

●

●

●

●

●

●

●

●

●

●

● ●

●

●

●

●

●

●

●●

●

●

●

●

●●

●●

●

● ●●●	● ●●

● ● ●

●

●

●

●

● ●

●

●

● ●

●●●● ●●

●

●●

●

●

●	● ● ● ● ●● ●

●●

●

●

●

●

●

●

●

● ● ●

●

●

●

●●

● ●

● ●

●

● ● ●

●●●●●● ●

●

●

●

●

●

● ● ●● ● ●

●

●

● ● ● ● ●

● ●

● ● ●● ● ●

●

●

●●●● ●● ● ● ●

●

0.75

0.50

3H

0.25

0.00

Normalized CO probability

1.00

●

●

●

●

●●

●

●

●

●

●

●

● ●

●

● ●

●

●

● ●

●

● ●

● ●

●●

●

●

●

●

●

●

●

● ● ● ●

●●

●

●

● ●

●	● ●●●

●

●●

●

●

● ●

●●

●

●

●

● ●

●

●

●

●

●

●

●

●

●

● ●

● ●

●●

●●●●

●

●

●

●

● ●●

●

● ●	● ●●●

●

●

●

●●● ●

●

●

●

●

●

●

●

●

●

●

●

●

●

●

●

●

●●●● ●

●

●

●

●

●

● ● ●●

● ●	●●● ●●●

●●● ●

●

●

●●

●

●

● ● ● ● ● ●●● ● ●●

● ●

●●

0.75

0.50

4H

0.25

0.00

1.00

●

●

●

●

●

●

●

●

● ●

●●

●

● ●

●

●

●

●

●

●● ● ● ●

●

●

●● ●

● ● ● ●● ●

●●● ●●●

●● ●●● ●●●

●

●

●

●

●

●

●

●

●

●

●

●

●

●

●

● ●

●

●

●

●

●

●

●

●

●

●

●

●

●

●

● ●

● ●

● ● ● ●

● ●●●

●● ●

● ●

● ●

● ●

● ● ● ● ●

●

● ● ●

● ●

● ●

● ●

● ● ●● ●

● ● ●

●

●

●

●

●

●

●

●

●

●

●

●

●

●●

●

●

●

● ●

●

●

●

●●

●● ●

●

●

●● ● ● ● ●● ●●

●●

●● ●	● ● ● ●

●

●

●	●●

● ●●●● ● ●●●●●●●●

● ●

●●●

0.75

0.50

5H

0.25

0.00

1.00

●

●

●

●

●

● ●

● ●

●

● ●●●

● ●●

●

●

●

●

●

●● ● ● ●

● ●● ● ●

●● ●

●

●

● ●

●

●

●

●

●

●

●

●

●

●

●

●

●

●

●

● ●

●

●

●

●

●

● ●●

●

●

●

●

●

● ● ●

●●● ●

● ●

●	●

● ●	●

● ●

●

●

●

●

●

●

●

●

●

●

●

●

●

●

●

●

● ● ● ●

●

●● ● ● ●●●

●

●

● ●

0.75

0.50

6H

0.25

0.00

1.00

●

●

●

●

●

●

●

●

● ●

●

●

●

● ●

●

● ● ● ●●	● ● ●	● ●

● ● ●

●

●

●

●

●	● ●

●● ● ● ●

●

● ● ●●●●

●

●

●

●

●

●

●

●

● ●

●

●

●

●

●

●

●

●

●●

●

●

● ● ●

●

●

●

●

● ●● ●●

● ● ●

●●

● ● ●

●

●

●

●

●

● ●

●

●

●

●

●

●

●

●

●

●

●

●

●

●	● ●

●●

●

●

● ●

●

●	●

●

● ● ● ●

●● ● ● ●

●

● ●● ● ● ●

●

●

●●● ● ●● ● ● ●

●

●

● ●

● ● ●●●●●●●●

● ●● ●

● ● ●

●

0.75

0.50

7H

0.25

0.00

0	200	400	600	0	200	400	600	0	200	400	600

Physical distance (Mbp)

Fig. S9: Normalized accumulated crossover (CO) probability (grey line), hotspot (red dots), and coldspot (turquoise triangles) 10 kb genomic windows across the seven barley chromosomes in the analyzed double round-robin (DRR) populations HvDRR13, HvDRR27, and HvDRR28. The vertical solid line in the back- ground indicates the position of the centromere in the v3 Morex reference genome and the vertical dashed lines indicate the pericentromeric region calculated for each chromosome and population.

HvDRR13	HvDRR27

454

(29.5%)

30

(1.9%)

408

(26.5%)

9

44 (0.6%) 44

(2.9%)	(2.9%)

551

(35.8%)

HvDRR28

A

HvDRR13	HvDRR27

15456

(11.9%)

12849

(9.9%)

26219

(20.1%)

21738

19670 (16.7%) 12961

(15.1%)	(10.0%)

21315

(16.4%)

HvDRR28

B

Fig. S10: Venn diagrams for the number of crossover (CO) hotspot (A) and coldspot

(B) 10 kb windows in the analyzed double round-robin (DRR) populations Hv- DRR13, HvDRR27, and HvDRR28.

HvDRR13	HvDRR27

379

(23.3%)

112

(6.9%)

360

(22.1%)

100

(6.1%)

139

(8.5%)

126

(7.7%)

413

(25.4%)

HvDRR28

Fig. S11: Venn diagrams for the number of gene conversion (GC) hotspot 10 kb windows in the analyzed double round-robin (DRR) populations HvDRR13, Hv- DRR27, and HvDRR28.

Fig. S12: Motif abundance in CO hotspot and background genomic windows. Mean counts of four previously reported crossover-associated DNA motifs within 10-kb CO hotspot windows and matched 10-kb background windows in distal and pericentromeric genomic regions of the barley genome. CO hotspots were identified independently in each population (HvDRR13, HvDRR27, and HvDRR28), and 10- kb windows overlapping hotspots in at least two populations were selected as CO hotspot windows for subsequent analyses. Background windows were sampled from the same chromosome and genomic region as the corresponding hotspot windows.

Fig. S13: Enrichment of DNA motifs in CO hotspot windows relative to matched genomic background. Fold enrichment of four previously reported crossover-associated DNA motifs in 10-kb CO hotspot windows relative to matched 10-kb background windows in distal and pericentromeric genomic regions of the barley genome. CO hotspots were identified independently in each population (HvDRR13, HvDRR27, and HvDRR28), and 10-kb windows overlapping hotspots in at least two populations were selected as CO hotspot windows for subsequent analyses. Enrichment was calculated as the ratio of mean motif counts in hotspot windows to mean motif counts in background windows. The dashed line at 1 indicates no enrichment or depletion. Motif classes comprise PolyA/T, CTT/GAA, CT/GA, and CCN/GGN.

Fig. S14: Chromosome-wide distribution of RNA-seq-derived SNPs across the seven barley chromosomes and three analyzed populations HvDRR13, HvDRR27, and HvDRR28. The number of SNPs was calculated in non-overlapping 10-kb windows along each chromosome for each population. SNP density is shown as the number of SNPs per 10-kb window, with chromosomes arranged as rows and populations as columns. The y-axis is constrained to 2,500 SNPs per window for visualization.
